# Nicotinamide and Sodium Butyrate Double the Pool of Treatment‐Exclusive Metabolites in the Endophytic Fungus *Curvularia* sp. G6‐32

**DOI:** 10.1002/cbdv.71774

**Published:** 2026-09-29

**Authors:** Guilherme Edison Almeida Pega, Laís Stella Perassolli Nicchio, Andressa Domingos Polli, Francielli Alana Pereira Valeze, Évelin Lemos de Oliveira, Eduardo Cesar Meurer, Débora Cristina Baldoqui, João Lúcio Azevedo, Julio Cesar Polonio

**Affiliations:** ^1^ Department of Biotechnology Genetics and Cellular Biology State University of Maringá Maringá Paraná Brazil; ^2^ Department of Chemistry State University of Maringá Maringá Paraná Brazil; ^3^ Jandaia do Sul Campus Federal University of Parana Jandaia do Sul Paraná Brazil

**Keywords:** epigenetic modulators, endophytes, mass spectrometry, metabolomics, molecular network, natural products, OSMAC

## Abstract

Endophytic fungi are prolific sources of bioactive natural products, but many biosynthetic gene clusters remain silent under laboratory conditions. Following the OSMAC approach, the epigenetic modifiers nicotinamide and sodium butyrate (10 µM) were added to triplicate liquid cultures of *Curvularia* sp. G6‐32, an endophyte of *Sapindus saponaria*, and the crude extracts were analyzed by UHPLC‐MS/MS with Mzmine 3 processing and GNPS molecular networking. In total, 712 features were detected: 474 common to all treatments, 65 exclusive to the control, and 154 occurring only in the presence of the modifiers (7 nicotinamide, 9 sodium butyrate, 138 both). PCA, ANOVA (*p* < 0.05) and volcano analysis (Student's *t*‐test, *p* < 0.05, *n* = 3) separated the treated cultures from the control. Among 34 putatively identified compounds, diketopiperazines were the most abundant family (15 ionic species), whereas loliolide, γ‐linolenic acid and N‐(3‐hydroxytetradecanoyl)‐DL‐homoserine lactone illustrate the diversification elicited by both modifiers.

## Graphical abstract

Epigenetic modulators nicotinamide and sodium butyrate reshape the chemical landscape of the endophytic fungus Curvularia sp. G6‐32. Combining the OSMAC strategy with UHPLC‐MS/MS metabolomics and molecular networking reveals a more than twofold rise in treatment‐exclusive metabolites, including diketopiperazines and potentially bioactive natural products, demonstrating that chemical elicitors awaken cryptic biosynthetic pathways for natural‐product discovery.

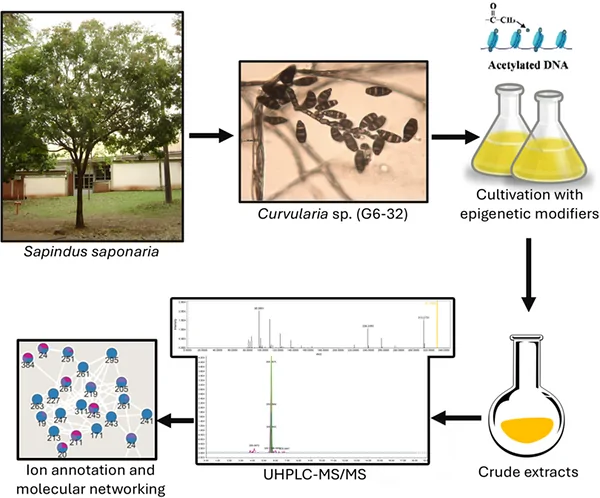

## 1 Introduction

Our planet hosts immense biodiversity within ecosystems defined by complex interactions. Among these, symbiotic relationships established between plants and endophytic microorganisms stand out. These fungi and bacteria inhabit the interior of plant tissues, colonizing the endosphere and establishing a mutually beneficial relationship [1]. While microorganisms find an ideal refuge, rich in nutrients and protected from external stresses, the host plant gains numerous advantages, including growth promotion, greater drought resistance, and protection against pathogens and abiotic stresses [2]. This ancestral symbiosis has shaped the evolution of many species, making it a promising research field in the search for sustainable agriculture and human health solutions.

Recent studies, such as those by Faria et al. [3], show that endophytes can modulate the plant defense system, not only protecting it directly but also strengthening its natural immune response. Furthermore, the capacity of these microorganisms to solubilize essential nutrients, such as phosphorus, and to produce phytohormones demonstrates their fundamental role in plant nutrition and development [4]. Within this vast universe of endophytes, the genus *Curvularia* emerges as a group of fungi of particular biotechnological interest. Belonging to the phylum Ascomycota, these filamentous fungi are recognized for their ecological versatility, existing as saprobes, epiphytes, and endophytes, while also exhibiting pathogenic behavior under certain conditions [5].

Their presence is widely reported in tropical and subtropical regions, with diverse species of *Curvularia* isolated from a variety of plants, including medicinal species. This endophytic lifestyle implies a unique capacity to produce a diverse range of secondary metabolites, such as alkaloids, polyketides, and terpenes, which are notable for their bioactive properties, such as antibacterial, antifungal, antioxidant, and anti‐inflammatory activities [6]. The discovery of compounds with activities of biological value, such as (‐)‐asperpentyn, highlights the importance of exploring the chemical potential of this genus [6]. Furthermore, the ability of some species of *Curvularia* to produce industrially relevant enzymes, such as cellulases and xylanases, expands their biotechnological potential beyond metabolite production [7].

Despite the biosynthetic potential of endophytic microorganisms, their exploitation on an industrial scale is limited by considerable challenges. Under laboratory culture conditions, far from their natural environment, fungi frequently lose the capacity to produce compounds of interest over time [8]. This phenomenon is directly related to the fact that most genes responsible for secondary metabolites production are “silenced” or “cryptic” and are not expressed under standard culture conditions [9]. Thus, this limitation suggests that the metabolic profile observed in the laboratory represents only a small fraction of the organism's actual genetic potential [8].

The recent understanding of biosynthetic gene clusters (BGCs), which are often found in regions of condensed chromatin, highlights that activating these genes is crucial for success in the discovery of new natural products [10]. The specific environment of the host plant, characterized by unique metabolites and stress conditions, acts as a potent inducer for the endophytic compounds biosynthesis, a stimulus that is lost in axenic culture and which explains the discrepancy between the potential and the observed metabolic profile [11].

To overcome these barriers, new culture and analysis strategies are necessary. A promising approach is epigenetic manipulation, which uses small molecules known as epigenetic modifiers, to activate silent genes. These substances modulate the chromatin structure and regulate gene expression without altering the DNA sequence [12]. One of the most explored epigenetic mechanisms involves histone acetylation. Histone deacetylases are grouped into distinct classes according to their catalytic requirements, and the available inhibitors are not equivalent in their coverage. Nicotinamide acts on the NAD‐dependent sirtuins, promoting histone hyperacetylation and, consequently, making genes more accessible to transcription [13]. Sodium butyrate, in turn, inhibits the zinc‐dependent deacetylases and has shown to be effective in modulating the metabolic profile, leading to the production of compounds not synthesized under standard culture conditions and significantly increasing the amount of specific metabolites [14, 15]. Since the two compounds therefore address different sets of enzymes, testing them in parallel allows the response of the strain to each class to be compared.

Recently, through the DNA methyltransfase modification mechanism, the chemical elicitor 5‐azacytidine led to the production of unique metabolites with antifungal activity and promoted the production of industrial relevant compounds [16, 17]. This approach, known as “One Strain Many Compounds” (OSMAC), enabling the induction of metabolites production that would not be synthesized otherwise [8]. Complementing this strategy, the use of metabolomics tools and molecular networks allows for a comprehensive analysis of the sample's chemical profile, facilitating the identification and characterization of new compounds. Platforms such as the Global Natural Product Social Molecular Networking (GNPS) have revolutionized the field by automating data analysis and enabling the discovery of structurally related molecular families [18]. The use of liquid chromatography coupled to high‐resolution mass spectrometry (LC‐MS/MS) is fundamental for this analysis, as it provides precise mass and fragmentation data that enable the identification of known compounds and the characterization of novel molecules [19]. Previous studies have already demonstrated the efficacy of this combination, such as the induction of coumarin production in *Pestalotiopsis crassiuscula* after treatment with the epigenetic modifier 5‐azacytidine [20].

Given the biotechnological relevance of the genus *Curvularia* and the challenges inherent to the production of its metabolites, the application of epigenetic modifiers combined with metabolomic analysis represents a powerful and innovative approach to unlock the unexplored biosynthetic potential of these fungi. Therefore, this study aimed to investigate the influence of the epigenetic modifiers nicotinamide and sodium butyrate on the modulation of the metabolic profile of a strain of *Curvularia* sp. (G6‐32). The metabolites were analyzed by ultra‐high‐performance liquid chromatography coupled to tandem mass spectrometry (UHPLC‐MS/MS) and, subsequently, submitted to metabolomic identification and molecular network analysis for the discovery of new compounds.

## 2 Results and Discussion

After drying the metabolic extracts, the yields of each treatment were measured, obtaining 47.20 mg for the control (PD); 150.70 mg for the nicotinamide treatment; and 73.90 mg for the sodium butyrate treatment. Data processing was performed in Mzmine revealed a total of 712 features, or chemical entities, produced in all treatments. Among them, 154 ions are present exclusively in the treatments with epigenetic modifiers, where 7 are exclusive to nicotinamide and 9 are exclusive to sodium butyrate. For the PD treatment, 65 exclusive ions were denoted. The observed ions and their relationships among treatments are illustrated in a Venn diagram (Figure 1) and in the heatmap (Figure 2), while the statistical variation can be observed on the PCA analysis (Figure 4). The heatmap analysis (Figure 2) reveals a greater spectral similarity between the samples treated with the epigenetic modifiers. Some ions formed groups by Ward clustering in which it was possible to highlight blocks with similar patterns. However, some divergences between the formed groups were also observed. A refinement of data interpretation and the identification of representative molecules for these groups are currently performed. Through comparison with spectral libraries imported into Mzmine, it was possible to obtain 51 match annotations for the detected ions. After filtering signals from known contaminants (e.g., plasticizers, cosmetic components such as sunscreens and repellents), 34 putatively identified compounds were obtained (Table 1) [21]. The chromatograms and fragmentation spectra related to all compounds presented on Table 1 are available on the Supporting Information (Figures S1–S34). The intensity of all detected features is available on Table S1.

**FIGURE 1:**
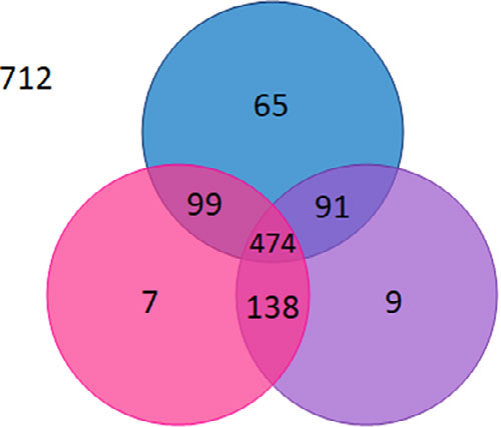
Venn diagram illustratively representing the distribution of ions observed after processing the raw UHPLC‐MS/MS data among the treatments PD (blue), nicotinamide (magenta), and sodium butyrate (purple) crude extracts. The value outside the circles represents the number of total ions in all treatments.

**FIGURE 2:**
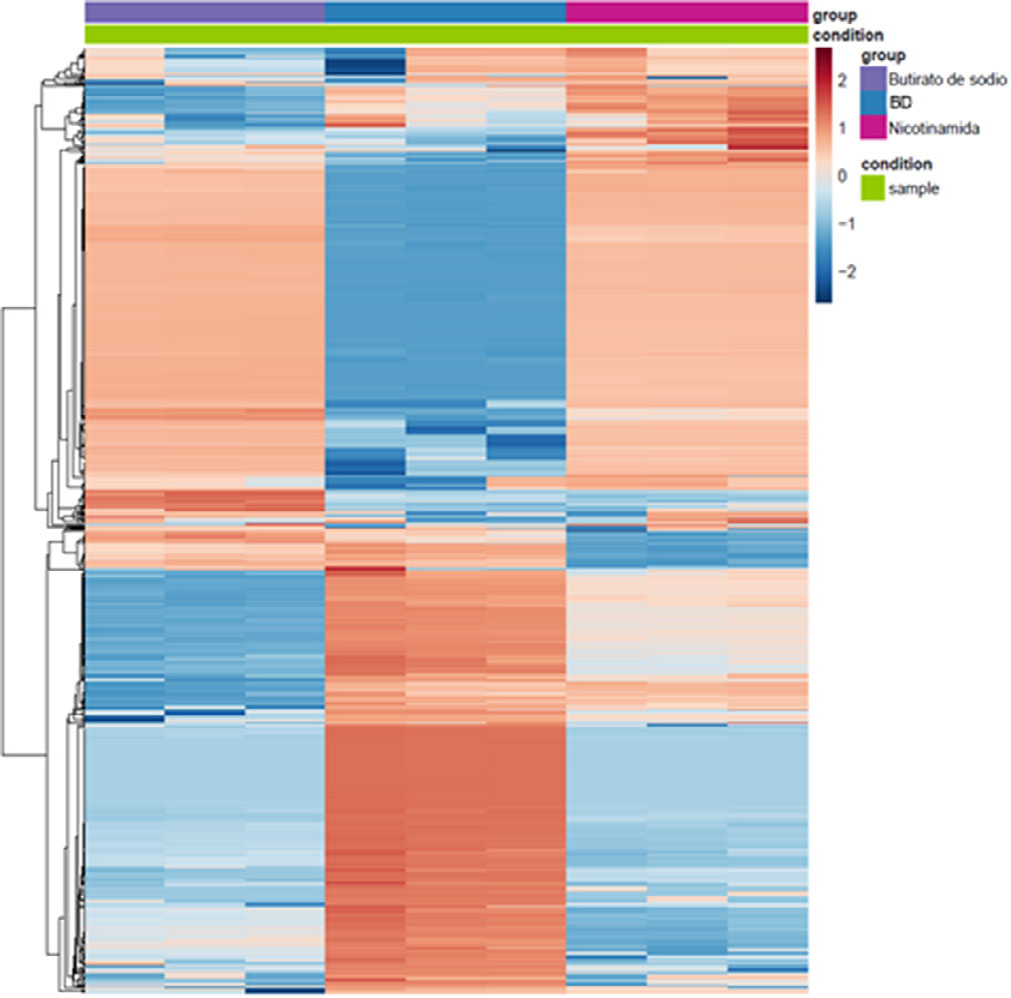
Heatmap of the raw UPLC‐MS/MS data among the treatments PD (blue), nicotinamide (magenta), and sodium butyrate (purple) crude extracts (*n* = 3).

**TABLE 1:** Compounds putatively identified in Mzmine from the UPLC‐MS/MS analysis of the crude extracts of the treatments PD (control), nicotinamide (A), and sodium butyrate (B), with the variation of their relative signal between each modifier treatment and the control.

							Treatment			Relative signal variation vs. control***			
ID	Annotation	Molecular formula	CAS*	RT** (min)	Experimental m/z	Error (‐/ppm)	PD	A	B	log2FC (A/PD)	p (A vs. PD)	log2FC (B/PD)	p (B vs. PD)
3	Pyridoxine	C8H11NO3	65‐23‐6	0.7319	170.0810	0.77	X	X	X	−1.52	0.260	−0.31	0.806
6	Glutamate	C5H9NO4	6106‐04‐3	0.7715	148.0601	2.78	X	X	X	−1.36	0.227	−1.75	0.195
11	Xanthine	C5H4N4O2	69‐89‐6	1.5776	153.0405	3.52	X	X	X	−0.80	0.534	−1.35	0.239
12	Cyclo‐(Ala‐4‐hydroxy‐Pro)	C8H12N2O2	—	1.7308	185.0920	5.20	X	X	X	−0.90	0.626	−0.44	0.808
13	Thymine	C5H6N2O2	65‐71‐4	1.8170	127.0496	4.59	X	X	X	+0.68	0.490	+0.87	0.573
14	Cyclo‐(Pro‐Gly)	C21H30N6O6	3705‐27‐9	2.0184	155.0814	5.39	X	X	X	+0.00	0.997	−1.27	0.033
15	Cyclo‐(Th‐Pro)	C9H14N2O3	227777‐31‐3	1.8624	199.1076	5.17	X	X	X	+1.40	<0.001	−0.71	0.002
18	Paeonol	C9H10O3	552‐41‐0	4.9256	167.0706	0.50	X	X	X	−1.25	0.033	−1.09	0.203
23	Cyclo‐(Gly‐Leu)	C8H14N2O2	5845‐67‐0	4.9917	171.1132	0.87	X	X	X	+0.07	0.909	−0.13	0.918
33	Cyclo‐(glycinyl‐L‐phenylalanine)	C11H14N2O3	—	5.6242	205.0945	6.59	X	X	X	+0.82	0.438	+2.17	<0.001
35	Cyclo(L‐Val‐L‐Pro)	C10H16N2O2	2854‐40‐2	5.7715	197.1288	3.70	X	X	X	−1.20	0.321	−0.09	0.905
46	3‐Isobutylidene‐hexahydro‐pyrrolo[1,2‐a]pyrazine‐1,4‐dione	C11H18N2O2	—	6.1691	209.1284	1.42	X	X	X	+0.48	0.032	+0.66	0.502
49	Cyclo‐(Ala‐Leu)	C9H16N2O2	24676‐83‐3	6.2623	185.1288	1.97	X	X	X	+0.31	0.810	−1.94	0.386
57	Isoquinolone	C9H7NO	491‐30‐5	6.4903	146.0602	2.34	X	X	X	−0.43	0.668	−1.27	<0.001
69	N‐acetyltyramine	C10H13NO2	1202‐66‐0	6.7858	180.1022	3.78	X	X	X	−0.99	0.118	+0.88	<0.001
81	Cyclo‐(L‐Leu‐L‐Pro)	C11H18N2O2	2873‐36‐1	7.0369	211.1447	4.30	X	X	X	−0.88	0.412	−0.35	0.751
103	Cyclo‐(D‐Trp‐L‐Pro)	C16H17N3O2	41222‐71‐3	7.3630	284.1400	4.79	X	X	X	+2.63	0.344	+0.35	0.834
108	Cyclo‐(L‐Phe‐D‐Pro)	C14H16N2O2	3705‐26‐8	7.4550	245.1291	3.80	X	X	X	−2.01	0.004	−0.90	0.160
117	Indole‐3‐acetic acid	C10H9NO2	87‐51‐4	7.6571	176.0704	4.46	X		X	n.d.	n.d.	−2.52	0.129
145	Cyclo‐(Val‐Leu)	C11H20N2O2	15136‐24‐0	8.0919	213.1601	3.97	X	X	X	+1.86	0.171	−0.03	0.421
152	Loliolide	C11H16O3	5989‐02‐6	8.2743	197.1178	4.40		X	X	n.d.	n.d.	n.d.	n.d.
154	Cyclo‐(Val‐Phe)	C26H38N8O7	14474‐71‐6	8.2965	247.1446	3.86	X	X	X	−1.85	0.185	−0.54	0.508
169	(2‐(4,4‐dimethyl‐4,5‐dihydrooxazol‐2‐yl)‐5‐fluorophenyl)(phenyl)methanol	C18H18FNO2	13447‐71‐5	8.5058	300.1278	4.07	X			n.d.	n.d.	n.d.	n.d.
194	Cyclo‐(Leu‐Leu)	C12H22N2O2	952‐45‐4	8.7795	227.1764	4.35	X		X	n.d.	n.d.	+0.13	0.938
200	Cyclo‐(Leu‐Phe)	C15H20N2O2	7280‐77‐5	8.8399	261.1605	4.07	X	X	X	−0.29	0.737	−0.54	0.690
217	N‐(3‐Hydroxytetradecanoyl)‐DL‐homoserine lactone	C18H33NO4	172670‐99‐4	9.9413	328.2494	2.37		X	X	n.d.	n.d.	n.d.	n.d.
378	(3S)‐5‐[(1S,8aR)‐2,5,5,8a‐tetramethyl‐4‐oxo‐4a,6,7,8‐tetrahydro‐1H‐naphthalen‐1‐yl]‐3‐methylpentanoic acid	C20H32O3	—	10.774	321.2427	7.53	X	X	X	−0.05	0.961	−1.40	0.249
381	Fa 18:3+1O	C18H30O3	—	10.841	295.2281	1.09	X			n.d.	n.d.	n.d.	n.d.
400	(10E,15E)‐9,12,13‐trihydroxy‐10,15‐octadecadienoic acid	C18H32O5	—	11.026	293.2112	1.45	X	X	X	+1.05	0.072	+1.39	0.248
411	5‐[(8aS)‐2,5,5,8a‐tetramethyl‐3‐oxo‐4a,6,7,8‐tetrahydro‐4H‐naphthalen‐1‐yl]‐3‐methylpentanoic acid	C20H32O3	—	11.139	321.2427	6.72	X	X	X	+0.16	0.004	−1.36	<0.001
418	Linoleic acid	C18H32O2	60‐33‐3	11.233	281.2486	1.03	X			n.d.	n.d.	n.d.	n.d.
512	Gamma‐Linolenic acid	C18H30O2	506‐26‐3	12.740	279.2315	0.31		X	X	n.d.	n.d.	n.d.	n.d.
596	D‐sphingosine	C18H37NO2	123‐78‐4	14.480	300.2883	2.41	X	X	X	−0.88	0.142	−0.13	0.921
619	Ceratodictyol	C19H38O4	—	14.807	331.2827	3.56	X	X	X	+2.59	0.343	+3.13	0.100

Presence in each treatment is indicated by X. The relative signal variation is expressed as the base‐2 logarithm of the ratio between the mean peak height of the treatment and that of the control, with the corresponding *p*‐value obtained by Student's *t*‐test on the biological triplicates (*n* = 3).

CAS*: https://commonchemistry.cas.org/; RT**: retention time; m/z***: mass‐to‐charge ratio; log2FC****: base‐2 logarithm of the ratio between the mean peak height of the treatment and the mean peak height of the control (*n* = 3), with the *p*‐value obtained by Student's *t*‐test; n.d.: not determined, corresponding to features detected in fewer than two replicates of one of the compared groups, for which a ratio is not defined, these cases being described by the presence markers in the Treatment columns. Peak height values are not directly proportional to concentration and comparisons are therefore restricted to the same ionic species across treatments. No correction for multiple comparisons was applied and the values are reported as exploratory.

The data analysis by volcano plot (Figure 3) demonstrated a significant variation in the total effects of the addition of nicotinamide (A) and sodium butyrate (B) on the metabolic profile, evidencing a greater or lesser quantity of compounds produced when compared to the PD treatment.

**FIGURE 3:**
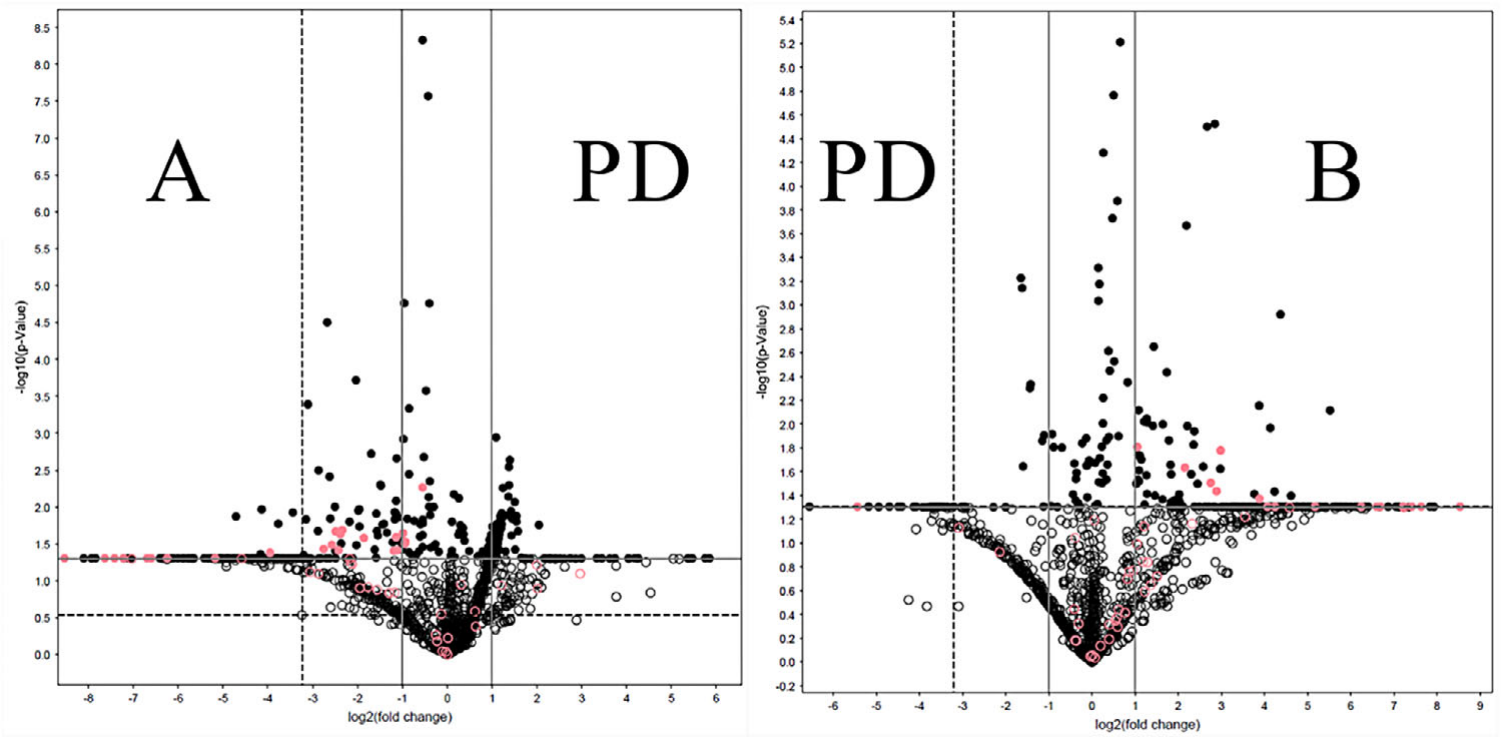
Volcano plot representing the variation of the metabolic profile of the extracts of the endophytic fungus G6‐32 *Curvularia* sp. between the treatments PD and nicotinamide (A) and PD and sodium butyrate (B) crude extracts. Each circle indicates a metabolite, where the black color indicates those not identified and those in pink indicate those identified in the spectral libraries (*n* = 3) (−log10 of the statistical *t*‐test, with *p*‐value on the y‐axis indicating the significance of the fold change versus log2 of the fold change between two groups on the x‐axis; the horizontal line drawn indicates the position of the *p*‐value = 0.05).

The principal component analysis (PCA) demonstrated the separation between nicotinamide, sodium butyrate and PD treatments (Figure 4). The PC1 axis explained 73.4% of total variance, while the PC2 explained 16.6%. In total, both components represented 90% of variability among treatments.

**FIGURE 4:**
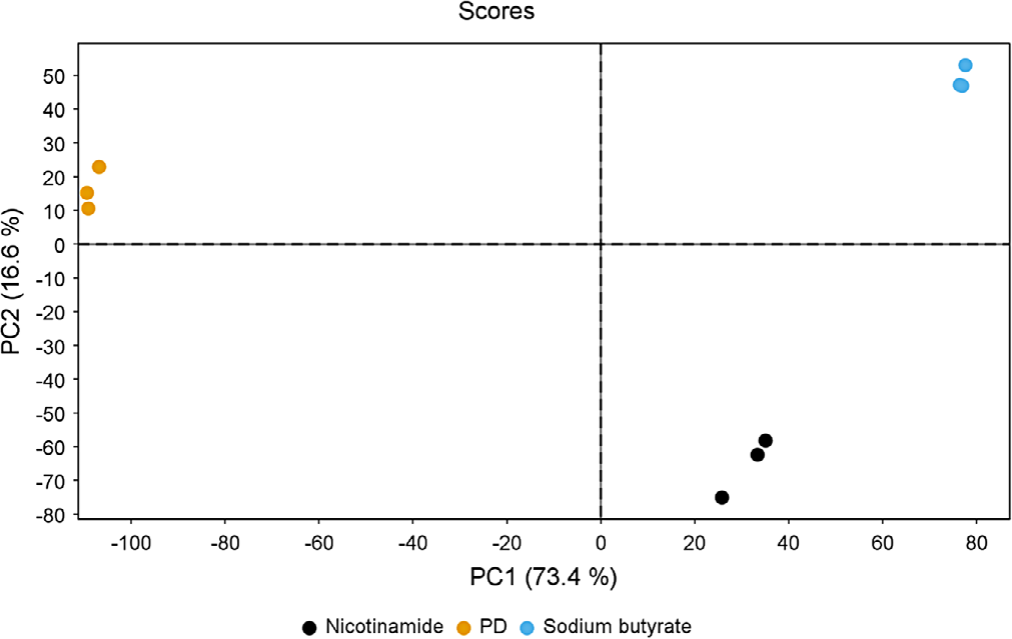
Principal component analysis (PCA) of the metabolic profile of the crude extracts of the endophytic fungus *Curvularia* sp. G6‐32 across the three treatments (PD, nicotinamide, and sodium butyrate). Each point represents a replicate (*n* = 3); PC1 and PC2 accounted for 73.4% and 16.6% of the total variance, respectively.

Additionally, for a better understanding and visualization of the molecular characteristics of the detected ions, the molecular network referring to the treatments was generated (Figure 5).

**FIGURE 5:**
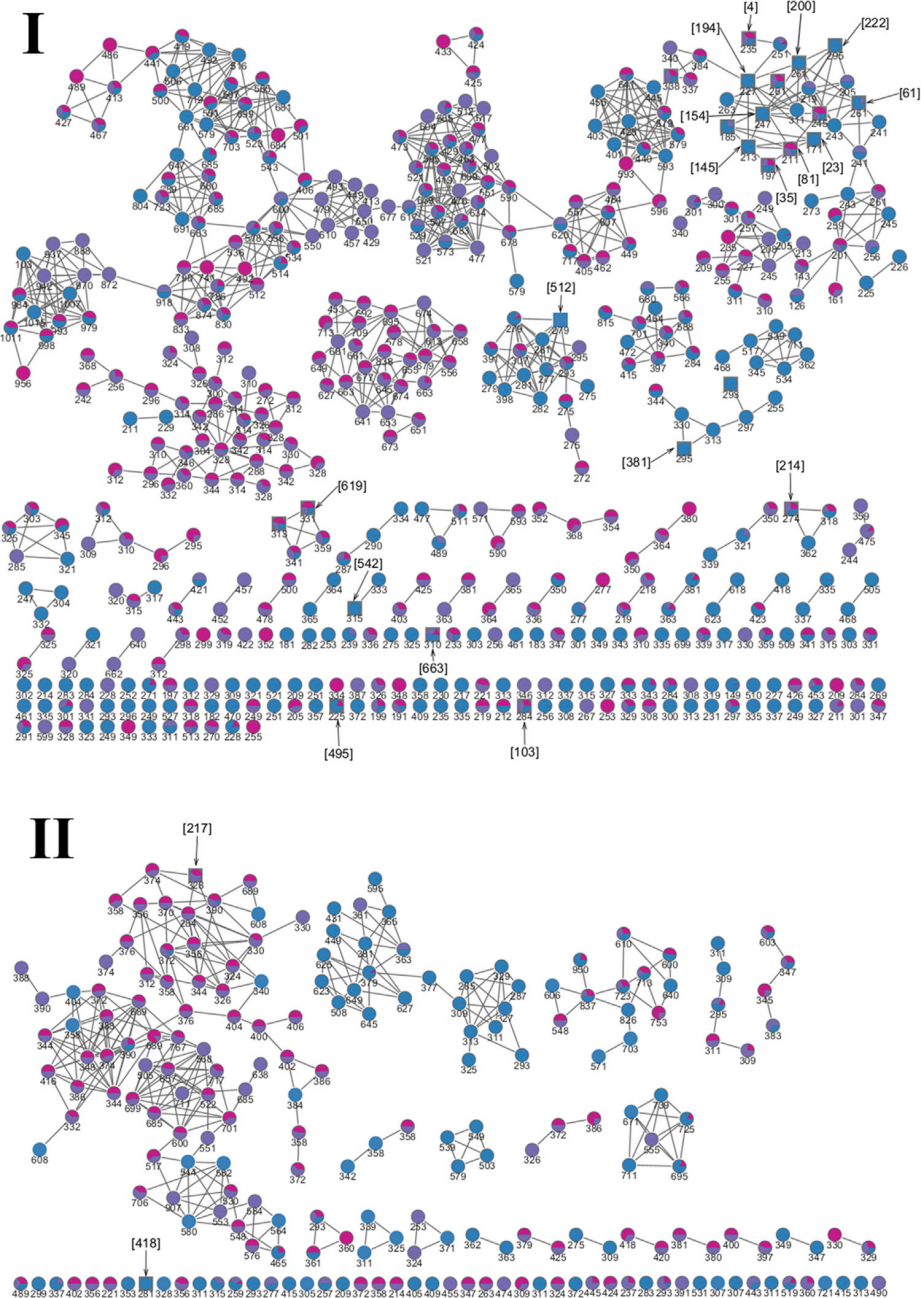
Molecular network generated through the GNPS platform from UPLC‐MS/MS analysis data among the treatments PD (blue), nicotinamide (magenta), and sodium butyrate (purple) crude extracts in positive (I) and negative (II) ionization modes. Numbers in brackets refer to the ID (Table 1) of putatively identified compounds in both GNPS and Mzmine spectral libraries. Numbers displayed under the nodes represent the experimental m/z value.

Analysis of the Venn diagram and the molecular network nodes reveals that the PD treatment was the one that presented the highest quantity of unique ionic entities (65). A possible hypothesis for this is that the addition of chemical elicitors activates a broader range of fungal metabolic pathways. Consequently, a greater number of compounds are biotransformed when compared to the standard treatment, as evidenced by the number of chemical entities shared between the treatments with the epigenetic modifiers (138).

Molecular network analysis showed that the cluster of molecules with the highest quantity of associated ionic species was “Cluster I” (identified by Mzmine), with a total of 517, and also the highest number of molecules matching with spectral databases. Within this set, the main common characteristic among the putatively identified molecules is the presence of at least one oxygen atom bonded to a carbon, including compounds from the classes of carboxylic acids, alcohols, aldehydes, amides, esters, and ketones.

The most abundant chemical family with identified molecules in Cluster I was the diketopiperazines (DKP), with a total of 15 ionic species. This family of molecules is characterized by cyclopeptides derived from the oxidative condensation of cyclic amino acids, such as alanine, phenylalanine, leucine, proline, tryptophan, among others. In general, it is an abundant family of cyclic alkaloids and diverse organisms are known to produce these natural compounds, where fungi stand out as the main source of acquisition. In fungi, diketopiperazines are associated with chemical defence and with the mediation of interactions with other microorganisms, and several members of the family act as signaling molecules that modulate microbial community behavior, including interference with bacterial quorum sensing [22, 23, 24].

Among the three culture conditions employed, the PD treatment presented the highest number of compounds in Cluster I and, consequently, the larger number of identified ions of the DKP family. It should be noted that this corresponds to a redistribution of the relative signal within the family rather than to the absence of these compounds, since diketopiperazines were detected under all three conditions. The direction of the variation differed between the two modifiers, most diketopiperazines showing a reduced relative signal under sodium butyrate while under nicotinamide the family showed no consistent displacement. A plausible interpretation is that the DKP pool was redirected toward enzymatic modification rather than suppressed at the level of synthesis. Diketopiperazines are highly stable against proteolysis and act as scaffolds for a variety of derived secondary metabolites, and the concurrent derepression of tailoring enzymes such as cytochrome P450s would consume these scaffolds and shift the detected signal toward their modified products [25, 26].

An example of DKP production by the metabolism of endophytic fungus was the discovery of ochrolines A, B, and C, produced by *Bionectria ochroleuca* SLJB‐2 (host plant *Selaginella doederleinii*) [27]. In 2018, Liu et al. isolated a new modified diketopiperazine, 3‐[6‐(2‐methylpropyl)‐2‐oxo‐1H‐pyrazin‐3‐yl]propanamide, from cultures of *Aspergillus versicolor* OUCMDZ‐2738 treated with the presence of SAHA.

Other chemical families present in Cluster I are fatty acids and lactones. Several studies describe and explore the capacity of endophytic fungi to produce fatty acids of industrial interest with the possibility of application in food processing and development, offering a faster, cheaper, and more ecological alternative compared to obtaining them through their host plant (mostly from seeds) [28].

The molecular network generated from the negative ionization mode data (Figure 5, network II) complemented the positive mode analysis by improving the coverage of metabolite classes that are preferentially ionized under negative conditions, such as fatty acids and N‐acyl homoserine lactones. Within this network, the annotation of N‐(3‐hydroxytetradecanoyl)‐DL‐homoserine lactone (ID 217) and linoleic acid (ID 418) reflected the differential effect of the cultivation conditions: while the homoserine lactone was detected exclusively in the cultures supplemented with the epigenetic modulators, linoleic acid was observed only in the control PD broth. The negative mode network also revealed condition‐specific clusters, such as the group of features at *m/z* 539, 548, 579, and 503, which were exclusive to the control condition (PD broth). These observations reinforce that data acquisition in both polarities broadened the surveyed chemical space and provided additional evidence of the metabolic diversification promoted by the epigenetic modulators.

γ‐Linolenic acid (GLA) (Figure 6) is a long‐chain polyunsaturated fatty acid (PUFA) that was observed only in fungal cultures treated with the presence of epigenetic modifiers, with a relative signal of higher intensity for the treatment with nicotinamide (A). This compound is characterized by the presence of an 18‐carbon chain, 3 double bonds (C‐6, C‐9, and C‐12), and a carboxyl group; factors that classify it as an ω ‐6 type PUFA, and its production is predominantly observed in eukaryotic organisms, mainly in algae, plants, and fish [29]. Although, GLA is reported as a fungal metabolite, being associated with the structure and function of cellular membranes, which can correspond up to 13% of total fatty acid content in some species [30, 31].

**FIGURE 6:**
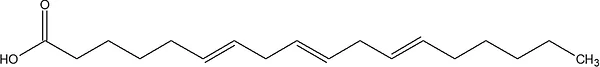
Chemical structure of γ‐linolenic acid.

The absence of GLA in the control broth and its detection in both modifier‐treated cultures indicate that the Δ6‐desaturation step, which converts linoleic acid into GLA, became operative only under the altered chromatin state. This interpretation is consistent with the reciprocal pattern observed in the negative‐mode network, in which linoleic acid (ID 418) was restricted to the control while its desaturation product was restricted to the treatments, and it reinforces the view of fungi as versatile platforms for the production of metabolites of applied interest [32].

The capacity of endophytic fungi to produce unsaturated fatty acids has been documented in other systems, and microbial sources are increasingly regarded as viable platforms for their supply [33], as in Xylariaceae sp. QGS 01, isolated from *Quercus gilva*, which accumulates a mixture of saturated and unsaturated fatty acids [34]. In fungi, GLA production has been addressed mainly through metabolic engineering, by overexpressing the desaturase genes of the biosynthetic route in *Mucor circinelloides* and *Aspergillus oryzae* [31, 35, 36]. The present result points to an alternative route to the same end. Rather than acting on the biosynthetic genes themselves, epigenetic modulation may relieve the transcriptional repression that keeps the pathway silent under standard cultivation. GLA of fungal origin has not, to our knowledge, been obtained previously by cultivation with epigenetic modulators, and confirmation by isolation and structural characterization would establish this as a route worth investigating.

Among the families of lactones, two of the putatively identified compounds are particularly notable (Table 1): N‐(3‐Hydroxytetradecanoyl)‐DL‐homoserine lactone (Figure 7A) and loliolide (Figures 7B), respectively.

**FIGURE 7:**
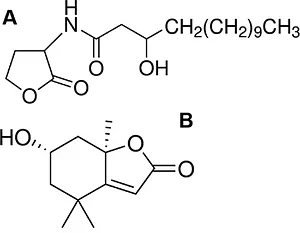
Chemical structure of the compounds N‐(3‐hydroxytetradecanoyl)‐DL‐homoserine lactone (A) and loliolide (B).

N‐(3‐hydroxytetradecanoyl)‐DL‐homoserine lactone belongs to the class of N‐Acyl homoserine lactones (AHLs). These molecules are composed of an acyl chain, which can range from 4 to 18 carbons, and a homoserine lactone ring (Figure 7A). It is mainly found in Gram‐negative bacteria as important molecules in cell communication, performing quorum‐sensing, being crucial in the regulation of cell density and coordination of collective bacterial behavior, but are also associated with various other biological functions, including the production of virulence factors, antibiotics, motility, and biofilm formation [37, 38]. The role of AHLs in the relationship between bacteria and plants is reported in the literature, which can positively affect the plant immune system by inducing the expression of proteins involved in the defense mechanism, resulting in resistance to pathogens [39, 40]. Furthermore, the activity of root growth promotion is also recognized as a main physiological response observed in the presence of AHL molecules [41]. In natural environments, plants are exposed to a wide variety of AHLs originating from their microbiota; however, the beneficial effects related to this class of compounds were also observed in plant crops with the addition of commercial AHLs [40].

Plants exhibit metabolic and physiological responses to AHLs due to their ability to detect molecules that are part of microbiological cell communication. However, the plant capacity to mimetize these molecules is known, interfering directly in processes regulated by quorum‐sensing in pathogenic bacteria [42, 43]. Similarly, Aiswarya [44] induced the production of these molecules in *Capsicum annuum L*. by inserting rhizobacterial genes via CRISPR‐Cas9, resulting in enhanced resistance to the phytopathogenic fungus *Fusarium oxysporum* f. sp. *capsici*. Thus, the putative annotation of N‐(3‐hydroxytetradecanoyl)‐DL‐homoserine lactone in the metabolic profile of G6‐32 suggests a potential application of the endophyte for obtaining improvements in plant defense and development, which can be explored in future investigation. Notably, N‐(3‐hydroxytetradecanoyl)‐DL‐homoserine lactone was detected in the modifier‐treated cultures, and was not recovered from the control broth.

Loliolide, a compound of the lactone family, is characterized by a monoterpenoid composed of 11 carbon atoms and widely found in diverse organisms, such as plants and animals in terrestrial and marine ecosystems, with emphasis on algae [45, 46]. Until now, there have been no reports of loliolide production by fungi and bacteria; its identity should be confirmed, as its putative production by the endophyte G6‐32 would be of particular interest. However, the compound (‐)‐loliolide is known as an important cell signaler in diverse plant species, associated with plant‐plant and also plant‐microbiota interactions, playing an important ecological role in response mechanisms to biotic and abiotic stresses of the plant system [47]. Thus, this suggests that the putative production of loliolide by the endophyte G6‐32 may be associated with the relationship of these microorganisms with the plant defense system and their capacity to mimic compounds produced by their host.

The discovery of compounds of the lactone class through fungal cultivation with the implementation of epigenetic modifiers has already been described in previous studies, such as in the unprecedented discovery of the molecules 5‐bromozeaenol and 3,5‐dibromozeaenol, together with four other analogues, produced by the marine fungus *Cochliobolus lunatus* TA26‐46, cultured with sodium butyrate [48]. Similarly, asperbutyrolactones A and B, which were obtained from the addition of 5‐AZA in the culture medium of the fungus *Aspergillus terreus* GZU‐31‐1, demonstrated potential anti‐inflammatory activity [49].

As shown in Figure 5, the parameters used to generate the molecular network resulted in additional molecular clusters with a large quantity of associated ions, but with the absence of any spectral signal attributed to a molecule identified by similarity analysis algorithms. A large number of isolated nodes that did not present sufficient similarity to forming a molecular cluster were also observed. Many of these isolated nodes correspond to the treatments with epigenetic modifiers, but, due to the dependence on the presence of spectral data of the structures in the databases, the chemical profile of the compounds referring to these signals could not be precisely identified, a scenario that demands future studies focused on the isolation and characterization of these molecules.

Previous studies performed by our research group reported the anticholinesterasic and antioxidant activities of the crude extracts of the endophytic fungus *Curvularia* sp. G6‐32 were described, as well as the isolation and characterization of (‐)‐asperpentyn, produced in high yield [6]. Consequently, the strategy of adding epigenetic modifiers in the cultivation of the endophyte was proposed following the observation of the loss of inhibitory potential against previously tested pathogenic strains, as well as reduced signals in chemical analyses aimed at metabolite detection (unpublished data). To resolve this point, a targeted analysis for (‐)‐asperpentyn was carried out on the extracts of the three treatments using the same instrumental and culture conditions described by Polli et al. [6]. Under these matched conditions, (‐)‐asperpentyn could not be detected in any of the analyzed extracts. This absence is consistent with the previously observed reduction of the strain's metabolite signals that motivated the present epigenetic‐modulation approach.

Through biological assays, it was not possible to observe anticholinesterasic, antioxidant, and antifungal activities in any of the fungal extracts derived from the treatments in this study. A plausible explanation for the absence of detectable bioactivity is the low absolute amount of metabolites in the extracts. Although the cultivation and assay conditions followed the same parameters described by Polli et al. [6], the present study used considerably smaller cultivation volumes, in line with its exploratory, metabolomic‐screening scope. This reduced total volume resulted in a lower absolute amount of secondary metabolites, which likely remained below the threshold required to elicit measurable activity in the assays.

Taken together, the modulated chemical classes suggest that the two modifiers acted on partly distinct portions of the biosynthetic repertoire. Nicotinamide and sodium butyrate act on distinct classes of histone deacetylases, as outlined above, and therefore do not address the same set of enzymes [13, 14]. Accordingly, 138 features were common to both treatments and absent from the control, while 7 features were restricted to nicotinamide and 9 to sodium butyrate, a pattern consistent with overlapping but not identical derepression of BGCs. The reduced relative intensity of diketopiperazines in the treated cultures is compatible with the redirection of these stable cyclodipeptide scaffolds toward downstream enzymatic modification, since the concurrent derepression of tailoring enzymes such as cytochrome P450s would consume them rather than suppress their synthesis [25, 26]. Integrated transcriptomic and metabolomic approaches have shown that sodium butyrate reshapes the expression of entire biosynthetic routes rather than individual genes [17], and an equivalent design would be required to link the chemical shift reported here to specific loci, either through targeted transcriptomics or through deletion of candidate acetyltransferase and deacetylase genes. It should also be noted that only the extracellular metabolites recovered from the fermented broth were analyzed in this study. The mycelial biomass, which may contain intracellular secondary metabolites, was not extracted, and the metabolic diversity reported here may therefore represent an underestimation of the total metabolome of strain G6‐32.

## 3 Conclusions

This study demonstrated the potential of the addition of the epigenetic modifiers nicotinamide and sodium butyrate to diversify secondary metabolite production by the endophytic fungus *Curvularia* sp. G6‐32. In total, the two treatments yielded 138 chemical entities, a value more than double that observed for the treatment without the addition of elicitors, among which it was possible to putatively annotate compounds of potential industrial interest. It should be emphasized, however, that all compounds reported here were annotated at a putative level, based on accurate mass, MS/MS fragmentation, and spectral library matching, and were not confirmed by isolation; by NMR or other orthogonal methods. Consequently, the biotechnological relevance and potential applications discussed throughout this work should be regarded as hypotheses that require future validation. In subsequent studies, this dataset may be further explored as spectral databases continue to expand, and chemical and microbiological approaches can be applied for the isolation, unequivocal structural characterization, and bioactivity evaluation of the compounds produced in the presence of the chemical elicitors.

## 4 Experimental Section

### 4.1 Microorganism

This study evaluated secondary metabolite production using the endophytic fungus *Curvularia* sp. G6‐32, isolated and identified by Garcia et al. [50], deposited in the Collection of Endophytic Microorganisms and from the Environment of the Laboratory of Microbial Biotechnology (CMEA‐LBIOMIC), located at UEM.

### 4.2 Culture Conditions and Epigenetic Modifiers

The endophytic fungus G6‐32 was inoculated in Petri dishes with potato dextrose agar (PDA) medium for seven days at 28°C. Next, three 5‐mm diameter discs from the fungal material were inoculated into nine Erlenmeyer flasks, each containing 20 mL of potato dextrose broth (PD). To evaluate the effect of epigenetic modifiers on metabolic production, three distinct culture media were performed: I) with addition of nicotinamide (C_6_H_6_N_2_O), II) with addition of sodium butyrate (C_4_H_7_NaO_2_), and III) a control group without addition of chemical modifier. All treatments had an incubation period of 21 days, as described by Polli et al. [6]; a final volume of 180 mL. The final concentration of epigenetic modifiers was 10 µM, based on previous studies that applied these modulators to endophytic fungi at comparable concentrations [51, 52]. Considering there is no report of the ideal concentration for both chemical modifiers in fungal fermentation, future studies will be conducted to explore the metabolic profile of G6‐32 at different levels of these epigenetic modifiers; as there is evidence of the inhibitory potential of sodium butyrate on fungal cells and a lack of information about the effects of nicotinamide [15].

### 4.3 Extraction of Metabolites

Following the incubation period, the fermented broth was filtered to separate the mycelium and obtain only the liquid phase. Metabolites extraction was performed by liquid‐liquid partition using ethyl acetate (EtOAc) and the filtrate in a 1:1 (v:v) ratio, in a 500 mL separating funnel, in three repetitions. The organic phase, containing the fraction of secondary metabolites of interest was concentrated under reduced pressure in a rotary evaporator at 37°C, to then obtain the metabolic extracts of each treatment.

### 4.4 Analysis of Metabolites by UHPLC‐MS/MS

The UHPLC‐MS/MS analyses of the extracts were conducted using an Impact II quadrupole time‐of‐flight (Q‐TOF) mass spectrometer (Bruker Daltonics Corporation, USA), at the Complex of Research Support Centers (COMCAP) of UEM. Chromatographic separation was performed using a Shim‐Pack XR‐ODS III C18 column (75 × 2.0 mm internal diameter, particle size of 1.6 µm) maintained at 40°C. The extracts were resuspended in methanol (10.0 mg/mL) and filtered through a 0.22 µm Sterile Polyethersulfone (PES) membrane filter. The injection volume was 3 µL. Gradient elution was performed using solvents A (water) and B (methanol in 0.1% formic acid) at 40°C, as follows: 0–1 min, 5% B; 1–3 min, 30% B; 3–12 min, 95% B; 12–15 min, maintained at 95% B; and 15–17 min, 5% B. Mass spectrometric detection was performed using an electrospray ionization (ESI) source. The capillary voltage was adjusted to 4500 V in positive and negative ionization mode, and sodium formate (10 µM) as calibrant. Drying gas parameters were: 8 L min^−1^, 200°C and 4 bar. Collision‐induced dissociation (CID) was performed using nitrogen (N_2_) collision gas and collision energy varying from 15–30 eV. Data were collected in the range of 50–1300 m/z in centroid mode with a mass resolution of 50 000 and a scan rate of 5 spectra per second. Data were analyzed using DataAnalysis version 4.3 (Bruker), with mass error calculated from molecular formulas and considered less than or equal to 10 ppm [53].

### 4.5 Metabolomic Identification and Molecular Network

To generate metabolite identification, raw UHPLC‐MS/MS data obtained in DDA (Data‐Dependent Acquisition) in positive and negative ionization were converted to the mzML file format using MSConvert software (ProteoWizard), applying the Peak Picking filter and then processed in the Mzmine 3 software (available at https://mzio.io/). Data collection was performed with a tolerance of 0.015 daltons (Da) for MS1 and 0.5 Da for MS2. Molecular formula prediction was processed exclusively with carbon (C), fluorine (F), hydrogen (H), oxygen (O), phosphorus (P), nitrogen (N), and sulfur (S) atoms. Peak detection was applied with an amplitude of 64 for minimum height, a minimum peak width equivalent to 5 Da, and a “mass slice” width of 0.1 Da. Deconvolution parameters were defined as follows: sigma window value of 0.5 and MS/MS abundance cutoff of 32 amplitudes [54].

Compound verification was performed using Mzmine's integrated tools for isotopic pattern matching and in silico fragmentation, comparing results against literature data, as well as the previous deconvolution. Potential candidate molecules for each metabolite were annotated by querying databases (including the Global Natural Product Social Molecular Networking—GNPS, MassBank, and MassBank of North America—MoNA) and then ranked by their similarity scores, calculated based on the comparison between experimental MS/MS spectra and those present in the databases [54].

Finally, molecular network was generated by importing the mzML files into the GNPS platform (http://gnps.ucsd.edu). The filter for edges, with a cosine score higher than 0.7 and at least 4 matching peaks, was established on the condition of 6 nodes similar to each other. Nodes annotation was achieved by spectral matching against the GNPS libraries and ranked according to their similarity (score) [55]. Finally, a table (Table S1) was exported with the intensity signals of features detected in each of the injections and a metadata text file was constructed for the identification of files corresponding to the samples. The table, in csv format, and the metadata file were uploaded to the Metaboanalyst web platform (https://www.metaboanalyst.ca/MetaboAnalyst/ModuleView.xhtml) and a heatmap was generated.

To (‐)‐asperpentin detection by mass spectrometry was used the same protocol described in Polli et al. [6].

### 4.6 Biological Assays

The antioxidant activity was evaluated to DPPH scavenging capacity and ABTS assay [56, 57]. Extracts were prepared according to Polli et al. [6]. The percentage of inhibition (I%) of DPPH and ABTS free radicals measures the results observed [56].

The anticholinesterasic activity of the crude extracts was quantified against Ache and BuChe through Ellman's method with modifications [6, 58]. Results are expressed as the concentration that inhibited enzyme activity by 50% (IC_50_) [6].

The antimicrobial activity was evaluated through the method for antifungal disk diffusion susceptibility testing for filamentous fungi, according to the CLSI M51 guideline with modifications [59]. All assays were conducted on PDA with the phytopathogens *Fusarium solani*, *Moniliophthora perniciosa*, and *Sclerotinia sclerotiorum*. Disks were embedded with extracts solubilized in methanol at the concentration of 10 mg.mL^−1^. Fluconazole was used as positive control (4 mg.mL^−1^) and pure methanol as negative control.

### 4.7 Statistical Analysis

The experiments were conducted in triplicate. PCA, analysis of variance (ANOVA), and Volcano Plot were performed on Mzmine 3 software (https://mzio.io/). PCA was performed using Auto Scaling to assess overall variance and sample clustering in response to treatments. To identify differentially expressed metabolites, ANOVA was employed with *p* < 0.05. Visualization and selection of the most significant features were carried out using a Volcano Plot through Student's *t* test, with a statistical significance threshold set at *p* < 0.05. In the Metaboanalyst web platform (https://www.metaboanalyst.ca/MetaboAnalyst/ModuleView.xhtml), the data matrix was subjected to logarithmic transformation and Pareto scaling, and Hierarchical cluster analysis (HCA) combined with a heatmap was generated using Euclidean distance as the dissimilarity metric and Ward's linkage algorithm. The graphical representation was constructed including only statistically significant features (FDR < 0.05). No correction for multiple comparisons was applied to the pairwise volcano analyses, and the differentially detected features are therefore reported as exploratory. The false discovery rate threshold of 5% was applied only to the hierarchical clustering and heatmap representation generated in MetaboAnalyst. All statistical analyses were conducted using peak height values.

## Author Contributions

**Guilherme Edison Almeida Pega**: conceptualization, data curation, investigation, methodology, writing – original draft. **Laís Stella Perassolli Nicchio**: conceptualization, formal analysis, investigation, methodology, writing – original draft. **Andressa Domingos Polli**: formal analysis, investigation, methodology, validation, writing – review and editing. **Francielli Alana Pereira Valeze**: data curation, investigation, methodology, writing – original draft. **Évelin Lemos de Oliveira**: investigation, methodology, resources, validation. **Eduardo Cesar Meurer**: investigation, methodology, resources, validation. **Débora Cristina Baldoqui**: formal analysis, methodology, resources, writing – review and editing. **João Lúcio Azevedo**: conceptualization, project administration, supervision, writing – review and editing. **Julio Cesar Polonio**: conceptualization, funding acquisition, project administration, resources, supervision, validation, visualization, writing – review and editing.

## Conflicts of Interest

The authors declare no conflicts of interest.

## Supporting information




**Supporting File**: cbdv71774‐sup‐0001‐SuppMat.pdf.

## Data Availability

The data that support the findings of this study are available from the corresponding author upon reasonable request.
