# Supplementary material for: Nicotinamide and Sodium Butyrate Double the Pool of Treatment‐Exclusive Metabolites in the Endophytic Fungus Curvularia sp. G6‐32
Source: Chem Biodivers. 2026 Sep 29;23(10):e71774. doi: 10.1002/cbdv.71774 (PMC13623109; doi:10.1002/cbdv.71774)
Supplement: Supplementary file 1 [file CBDV-23-e71774-s001.pdf]

Supplementary Material

Chromatograms of the features presented on Table 1.

Figure S1. Chromatogram obtained through UPLC corresponding to the Pyridoxine signal. m/z 170.08. Detected on 0.73 min.

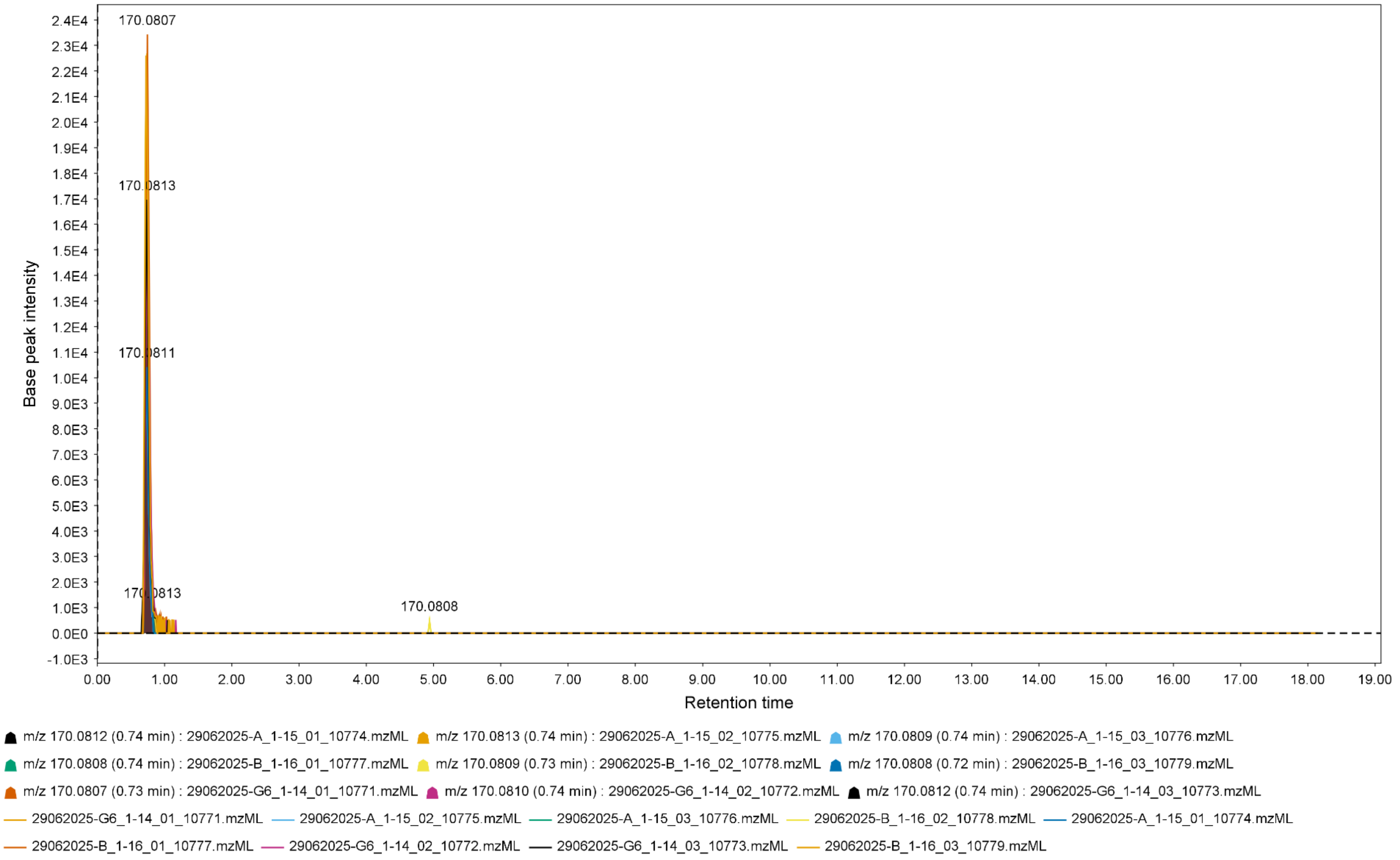

Figure S2. Chromatogram obtained through UPLC corresponding to the Glutamate signal. m/z 148.06. Detected on 0.77 min.

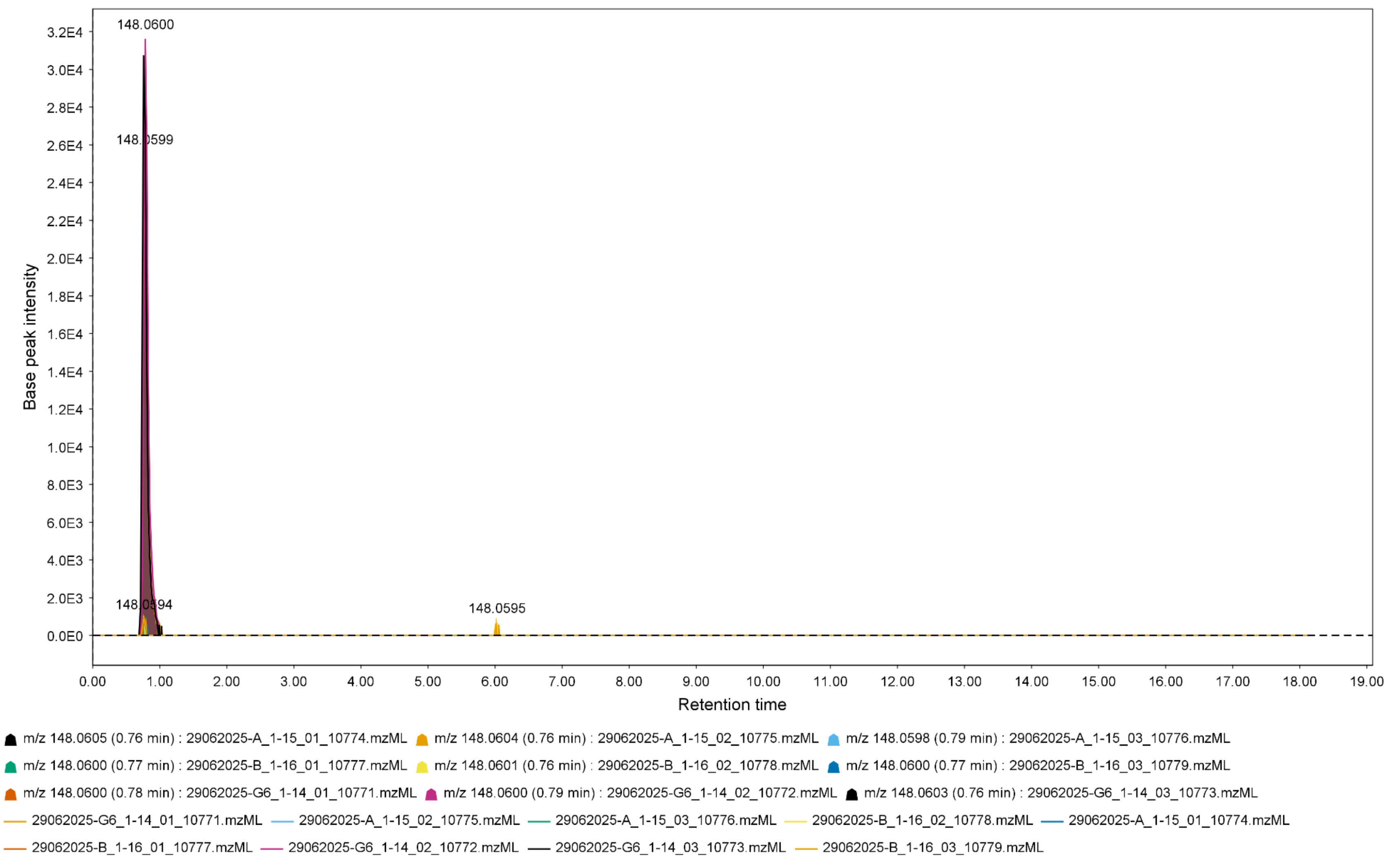

Figure S3. Chromatogram obtained through UPLC corresponding to the Xanthine signal. m/z 153.04. Detected on 1.58 min.

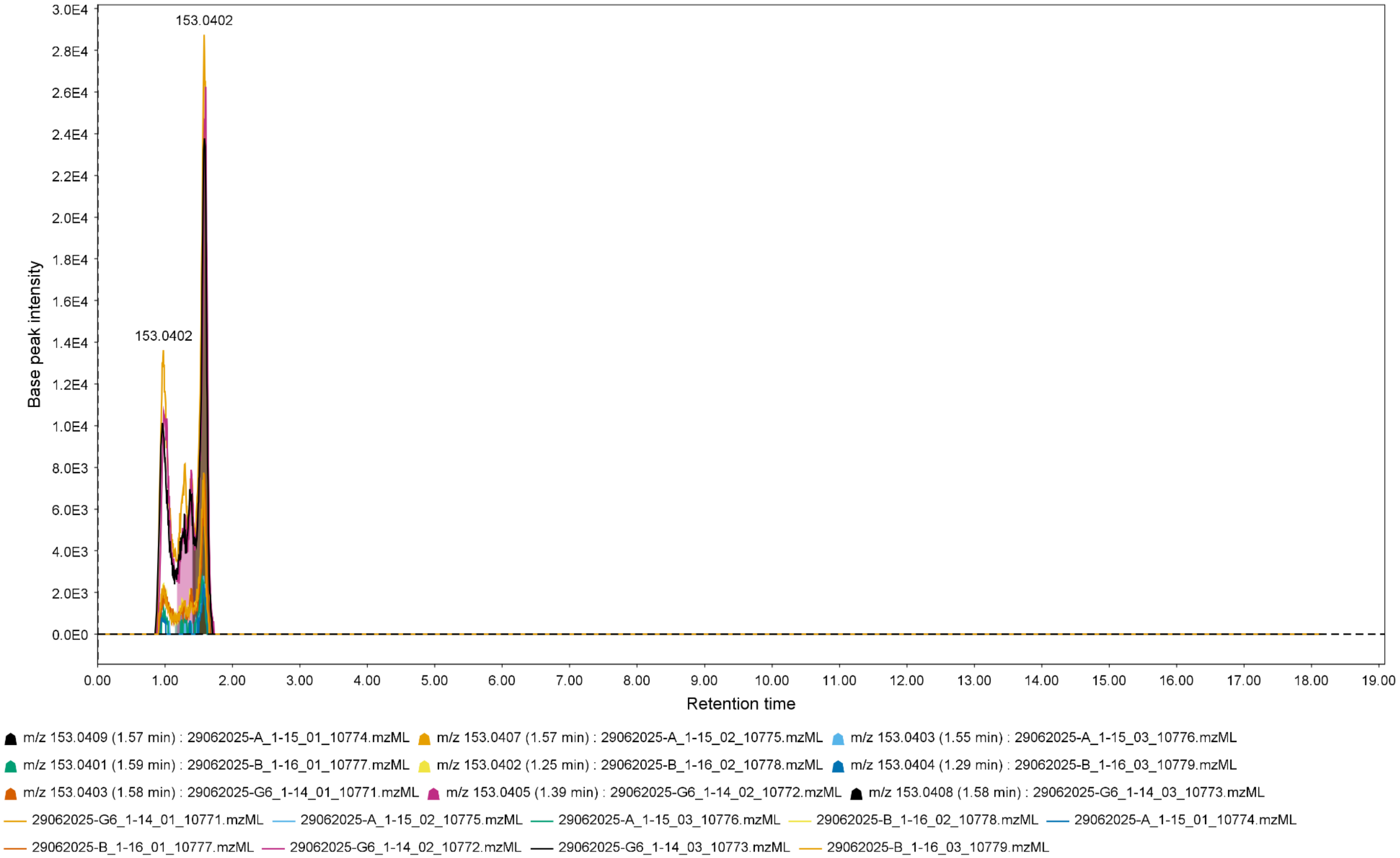

Figure S4. Chromatogram obtained through UPLC corresponding to the Cyclo-(Ala-4-hydroxy-Pro) signal. m/z 185.09. Detected on 1.73 min.

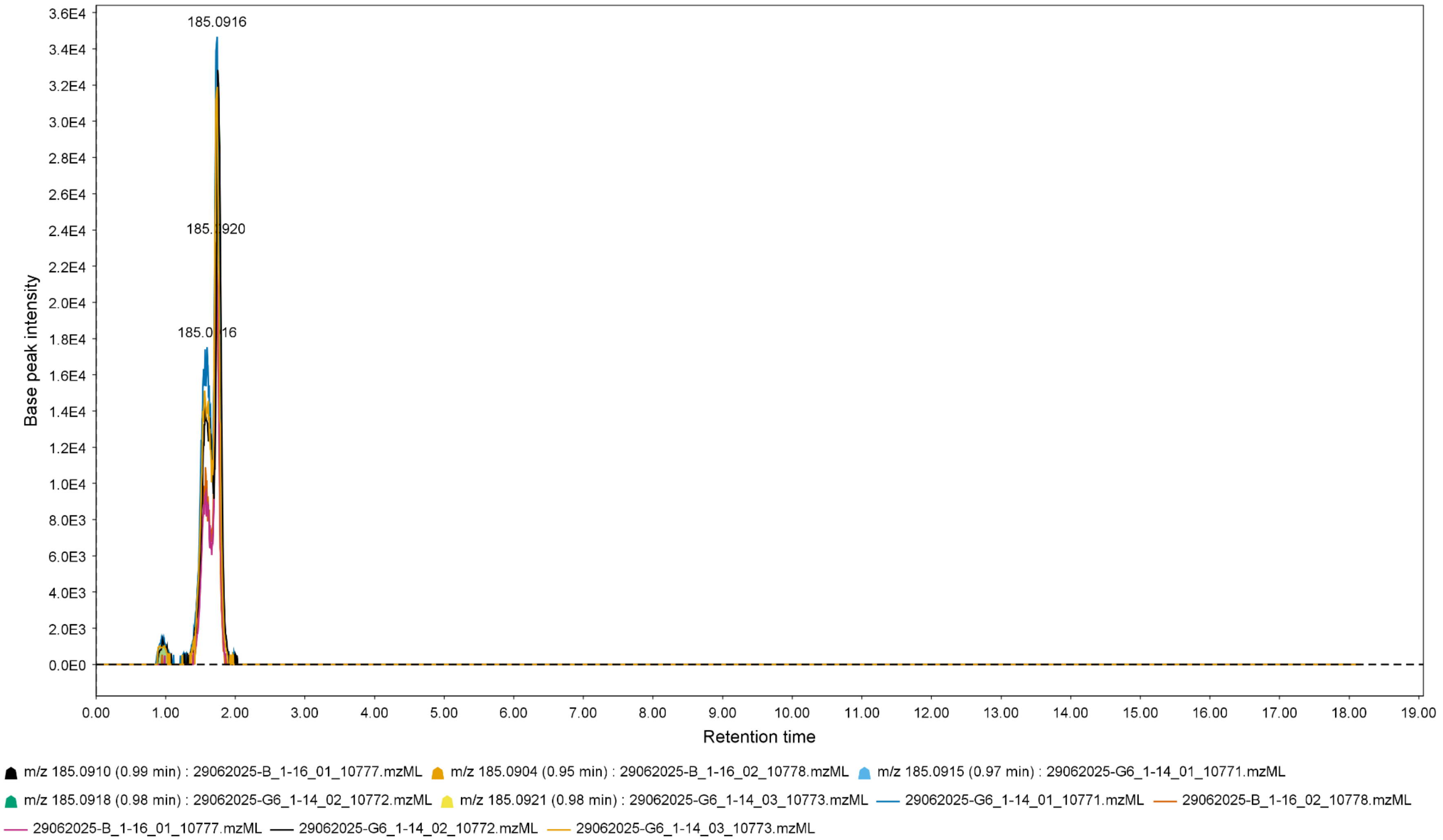

Figure S5. Chromatogram obtained through UPLC corresponding to the Thymine signal. m/z 127.05. Detected on 1.82 min.

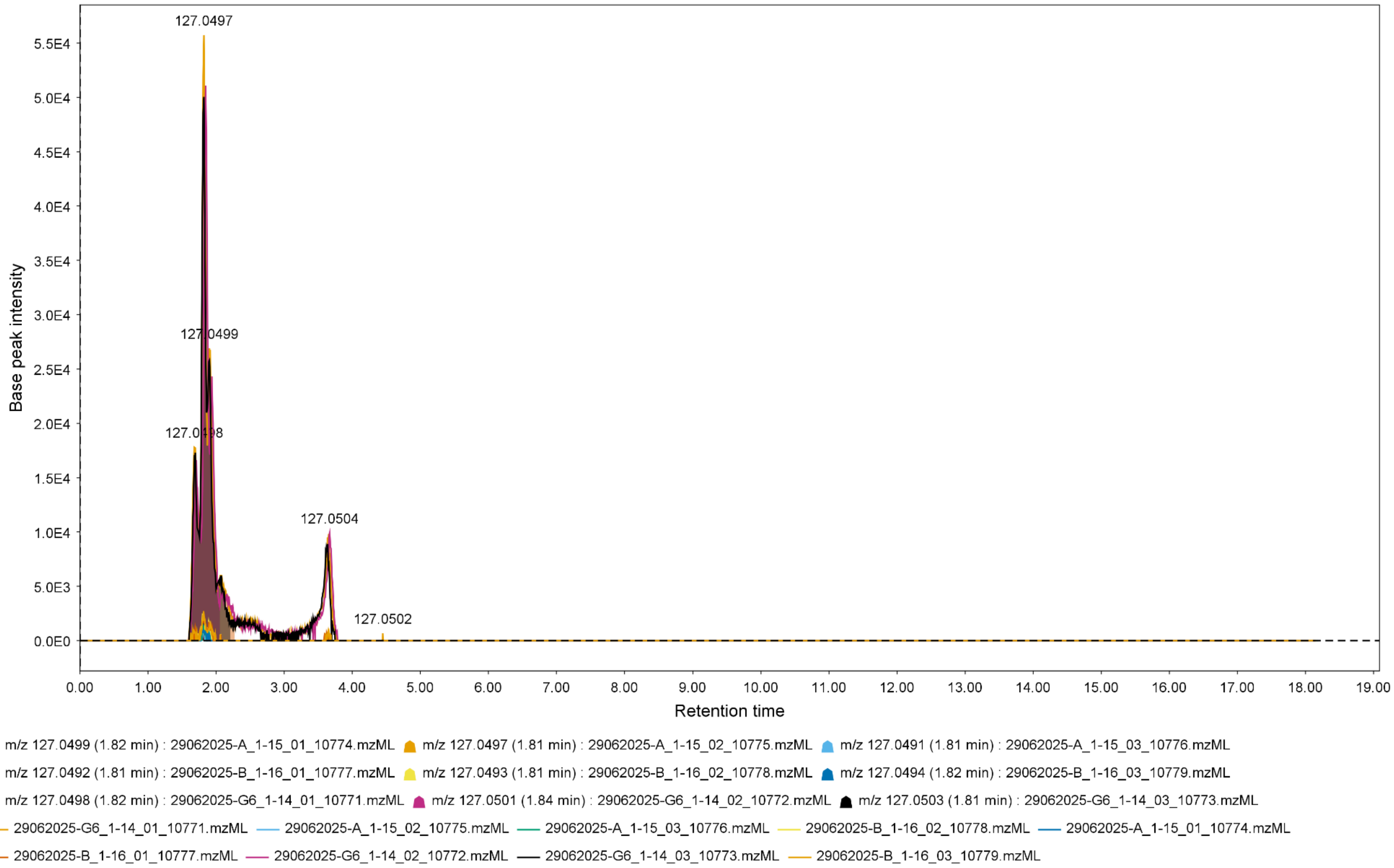

Figure S6. Chromatogram obtained through UPLC corresponding to the Cyclo-(Pro-Gly) signal. m/z 155.08. Detected on 2.02 min.

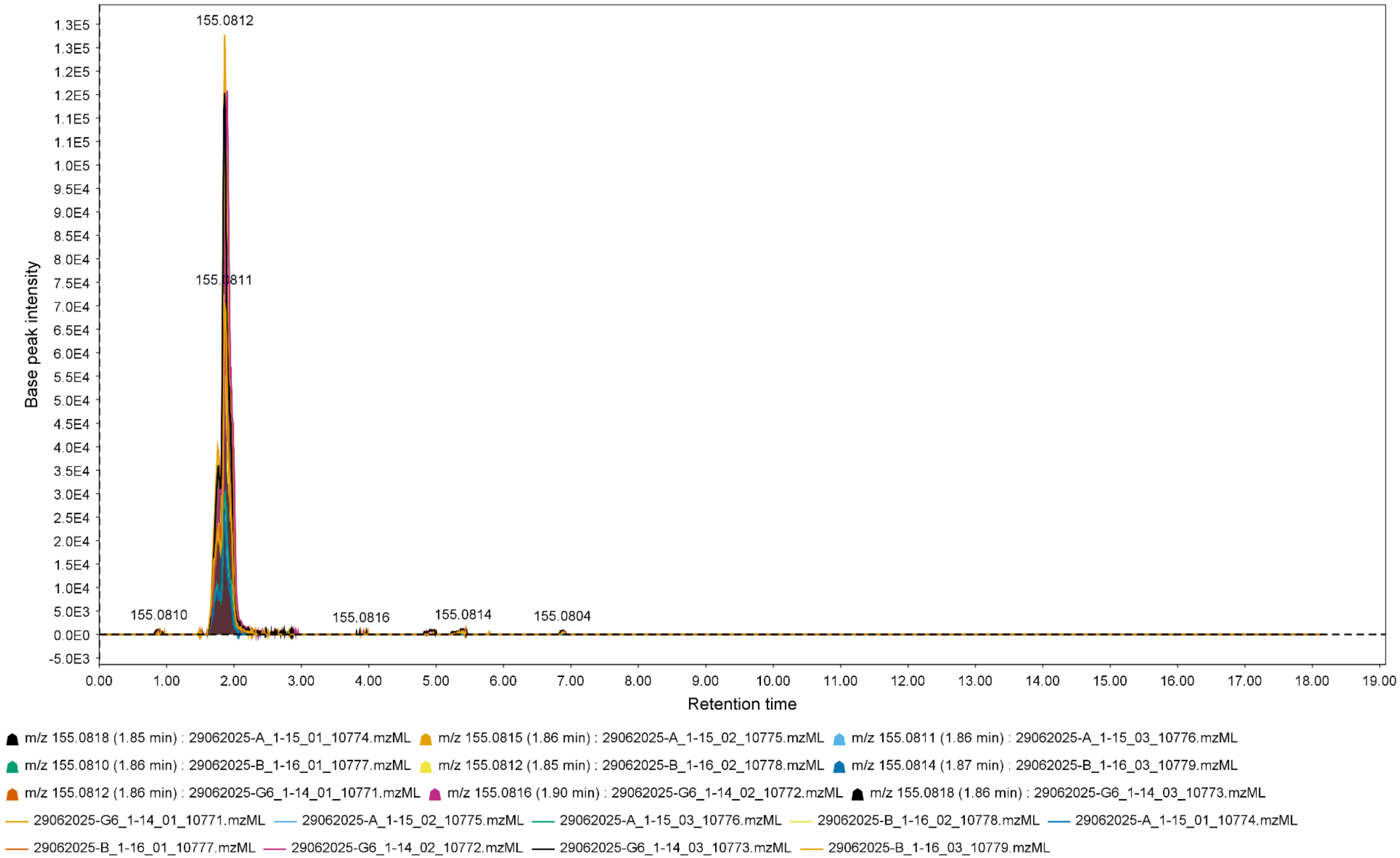

Figure S7. Chromatogram obtained through UPLC corresponding to the Cyclo-(Th-Pro) signal. m/z 199.11. Detected on 1.86 min.

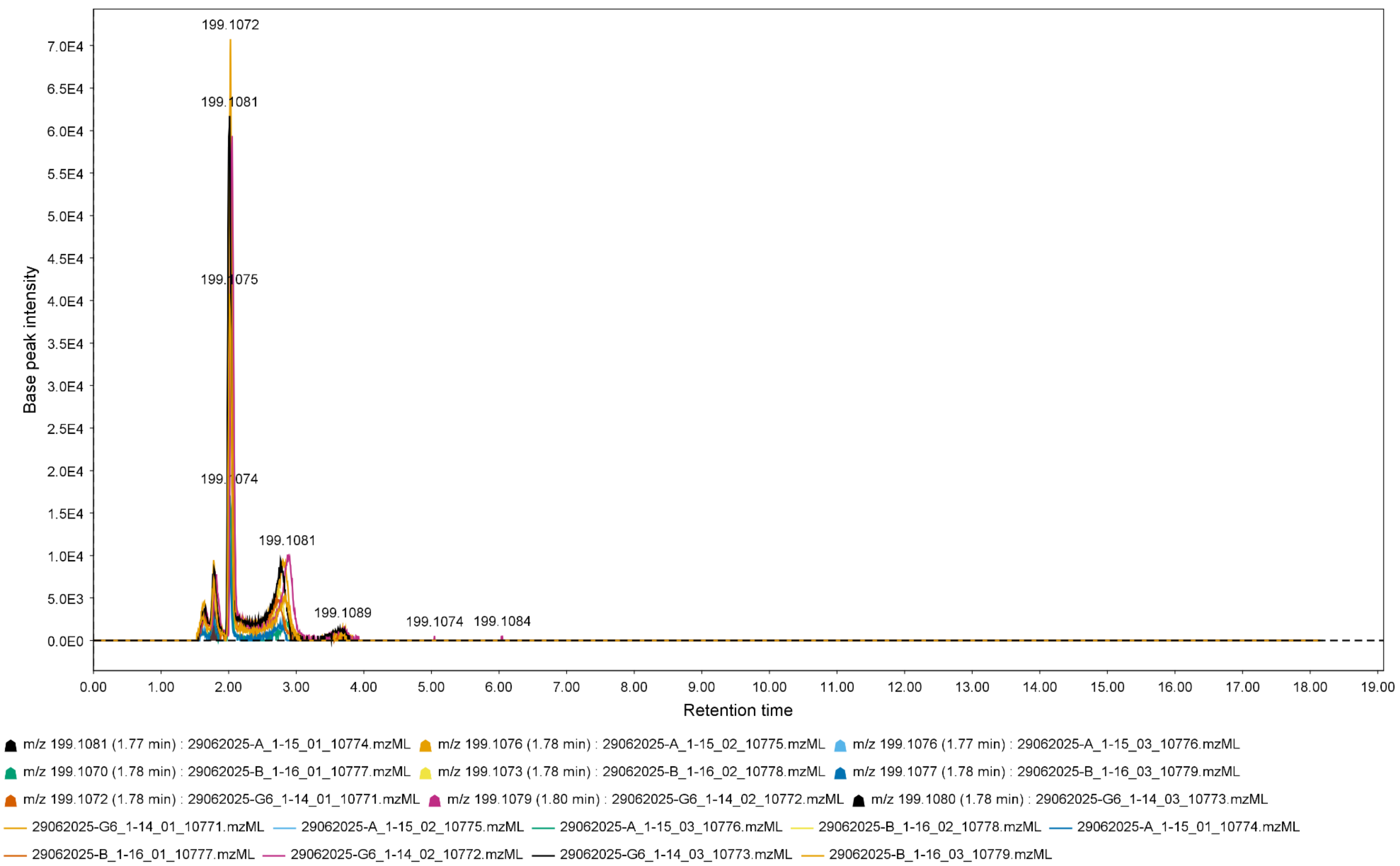

Figure S8. Chromatogram obtained through UPLC corresponding to the Paeonol signal. m/z 167.07. Detected on 4.93 min.

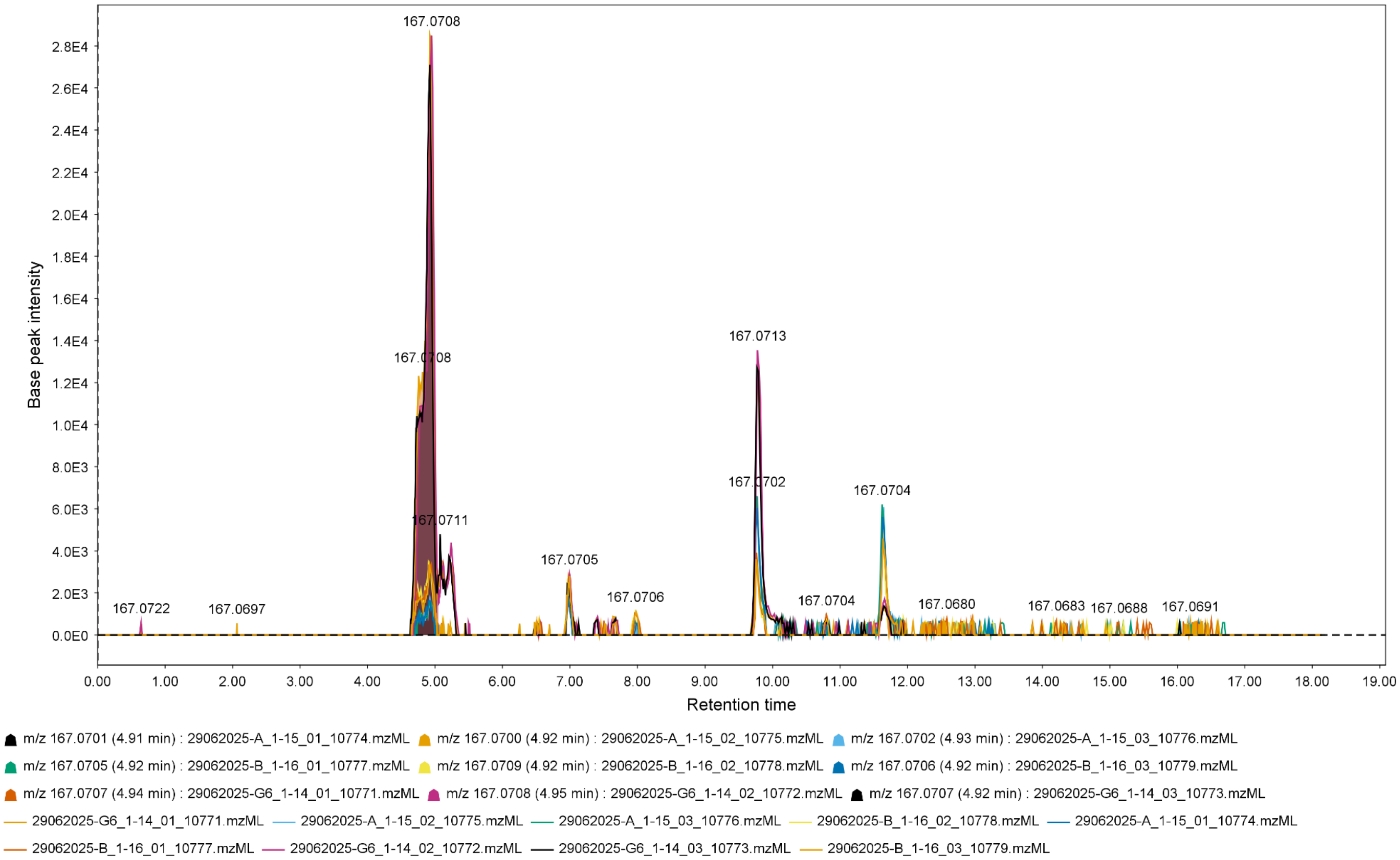

Figure S9. Chromatogram obtained through UPLC corresponding to the Cyclo-(Gly-Leu) signal. m/z 171.11. Detected on 4.99 min.

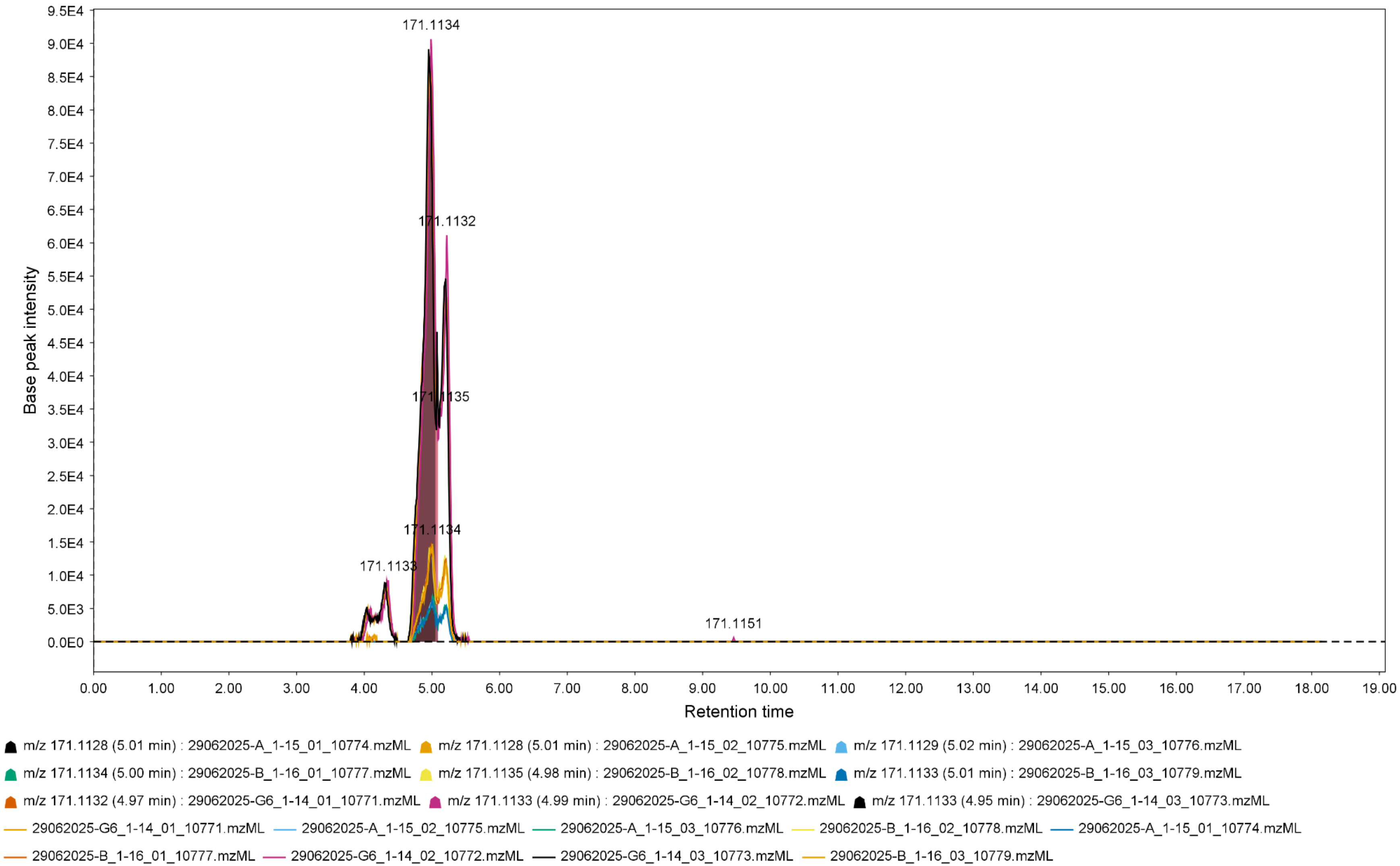

Figure S10. Chromatogram obtained through UPLC corresponding to the Cyclo-(glycinyl-L-phenylalanine) signal. m/z 205.09. Detected on 5.62 min.

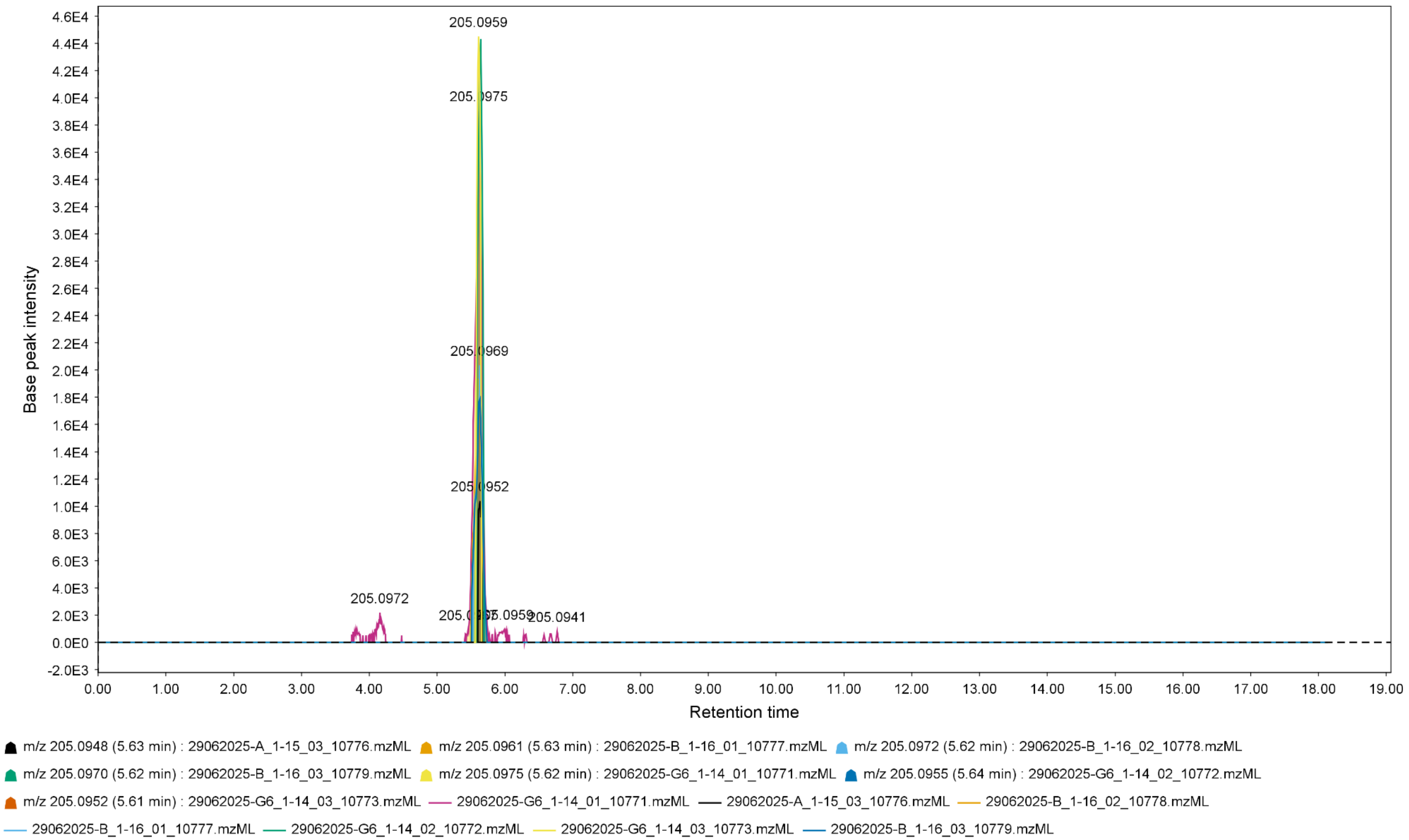

Figure S11. Chromatogram obtained through UPLC corresponding to the Cyclo(L-Val-L-Pro) signal. m/z 197.13. Detected on 5.77 min.

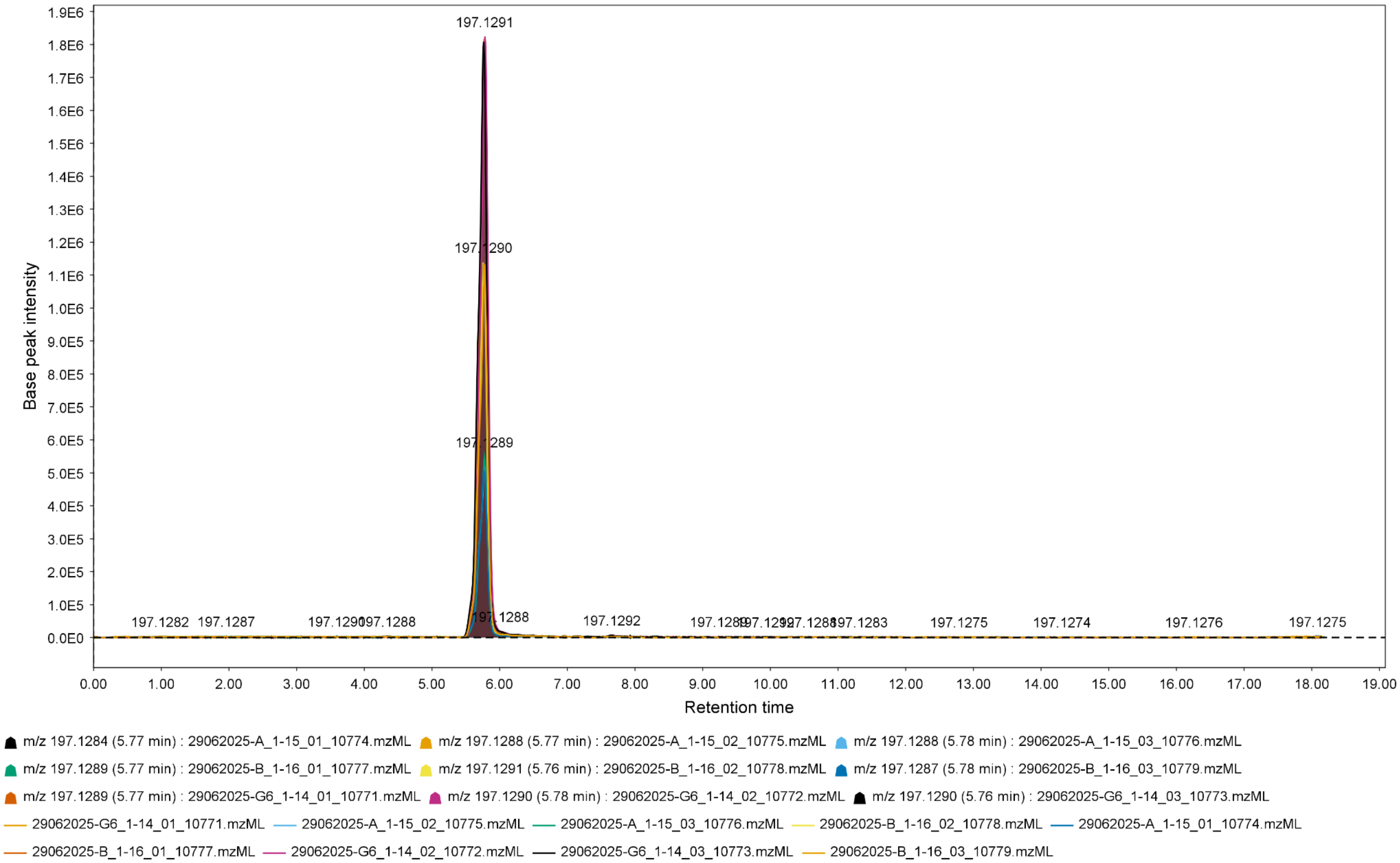

Figure S12. Chromatogram obtained through UPLC corresponding to the 3-isobutylidene-hexahydro-pyrrolo[1,2-a]pyrazine-1,4-dione signal. m/z 209.13. Detected on 6.17 min.

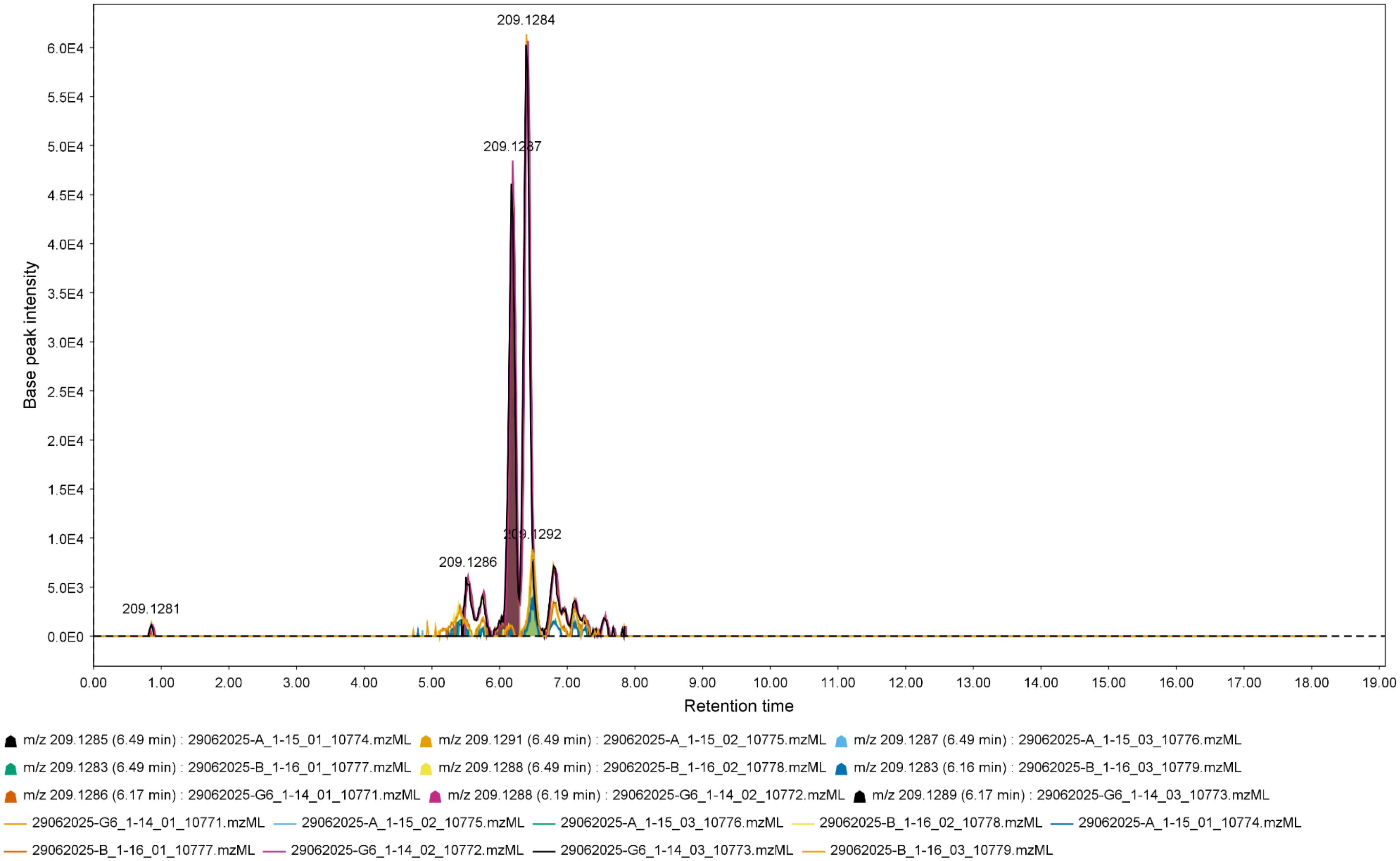

Figure S13. Chromatogram obtained through UPLC corresponding to the Cyclo-(Ala-Leu) signal. m/z 185.13. Detected on 6.26 min.

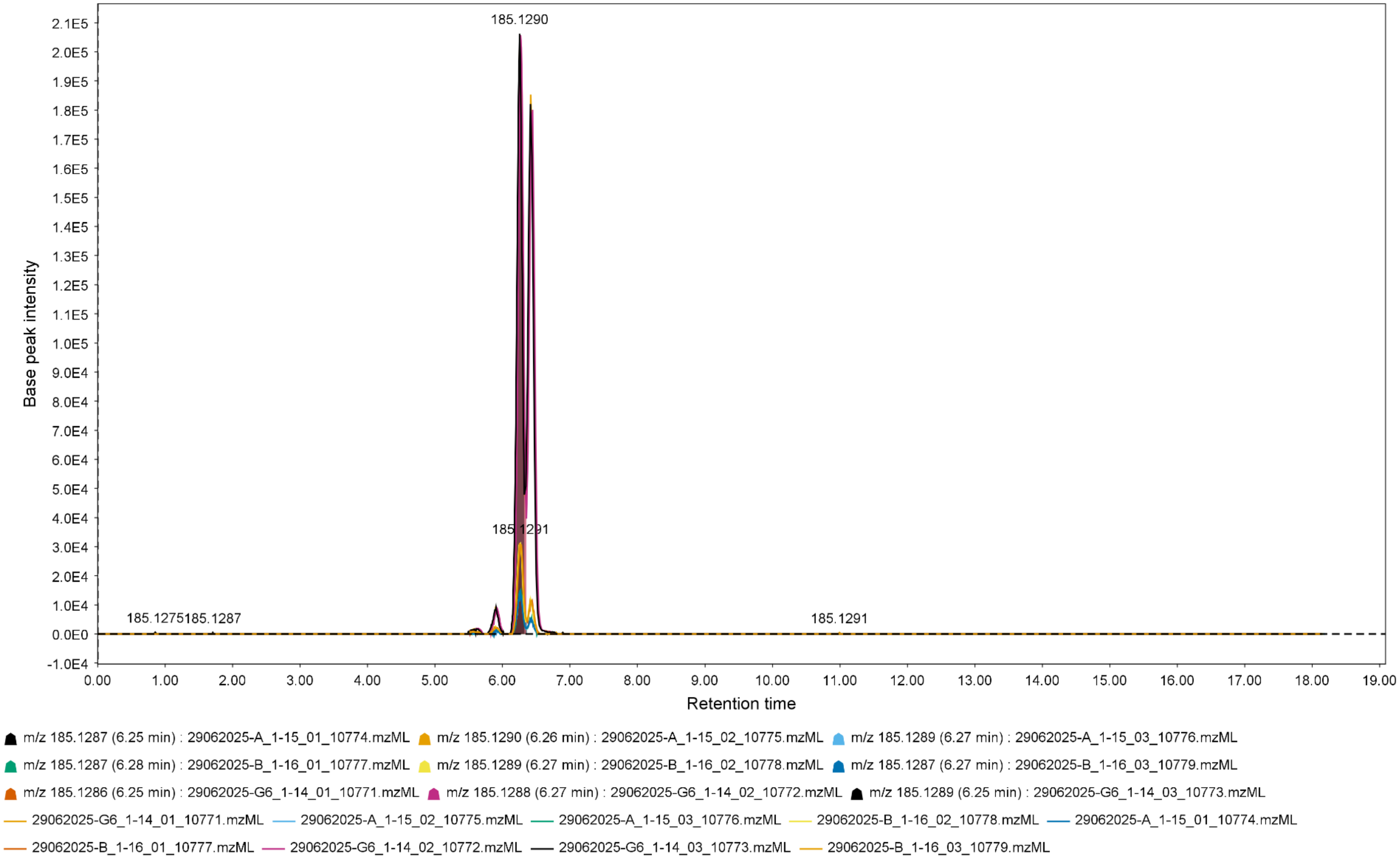

Figure S14. Chromatogram obtained through UPLC corresponding to the Isoquinolone signal. m/z 146.06. Detected on 6.49 min.

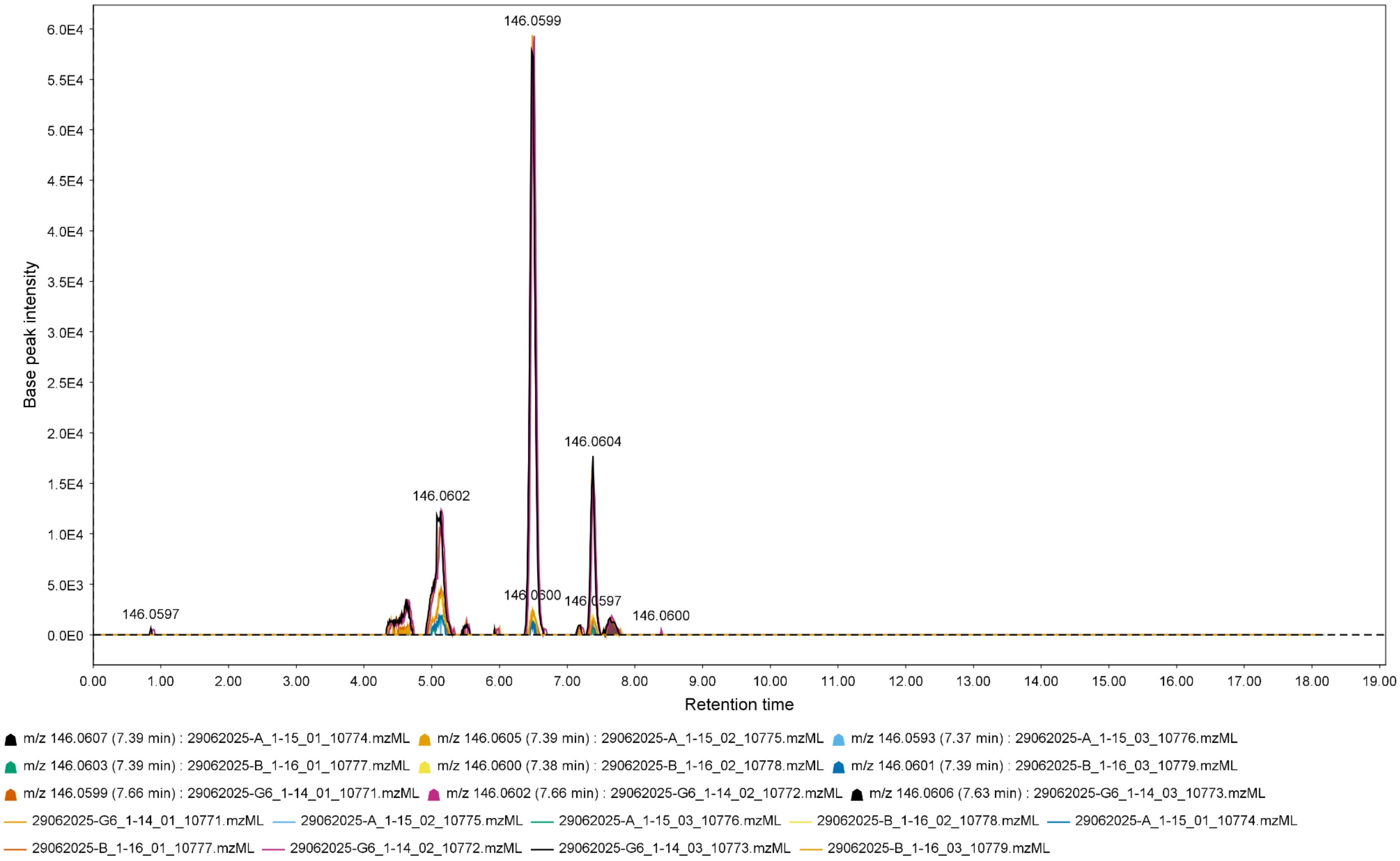

Figure S15. Chromatogram obtained through UPLC corresponding to the N-acetyltyramine signal. m/z 180.10. Detected on 6.79 min.

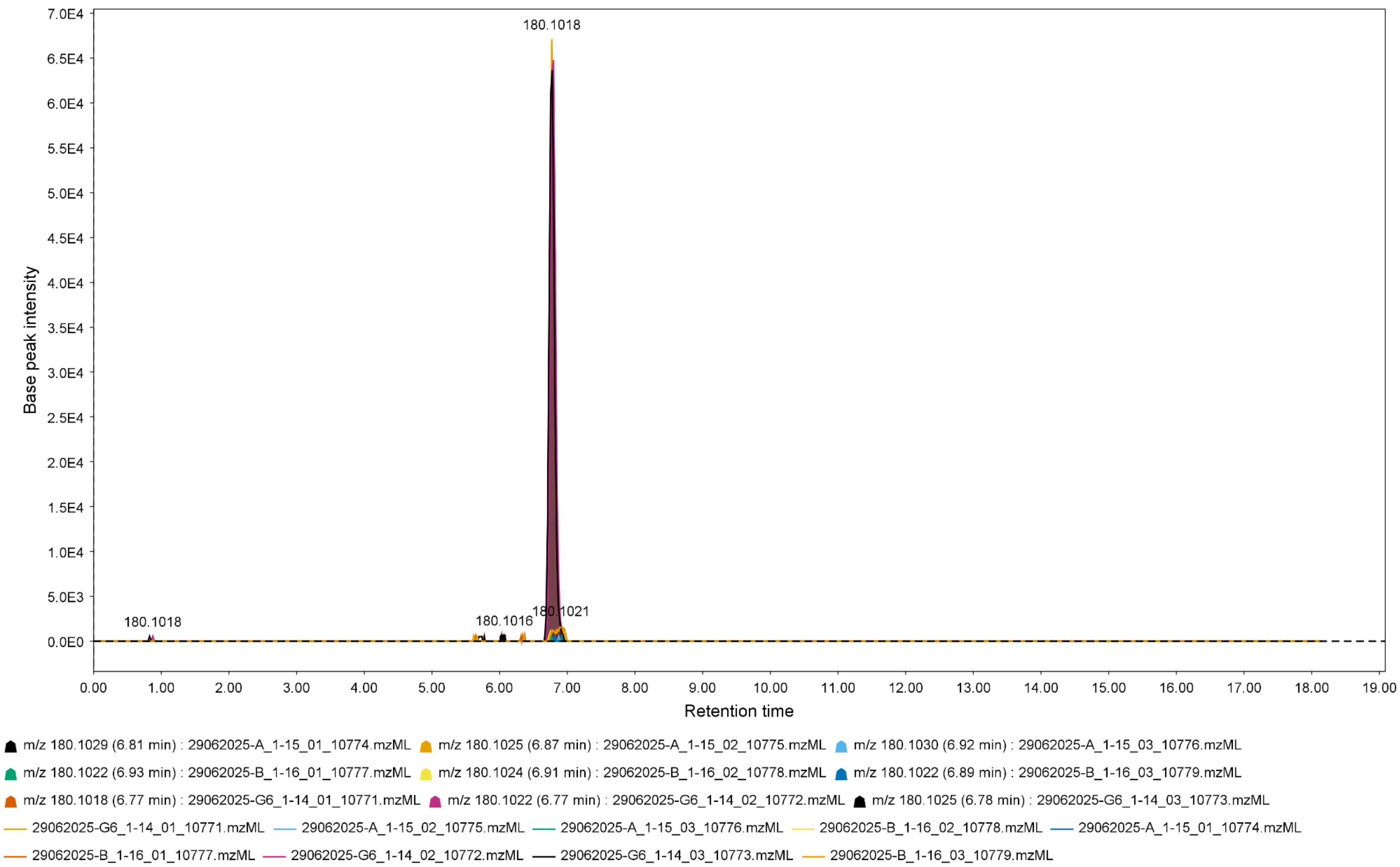

Figure S16. Chromatogram obtained through UPLC corresponding to the Cyclo-(L-Leu-L-Pro) signal. m/z 211.14. Detected on 7.04 min.

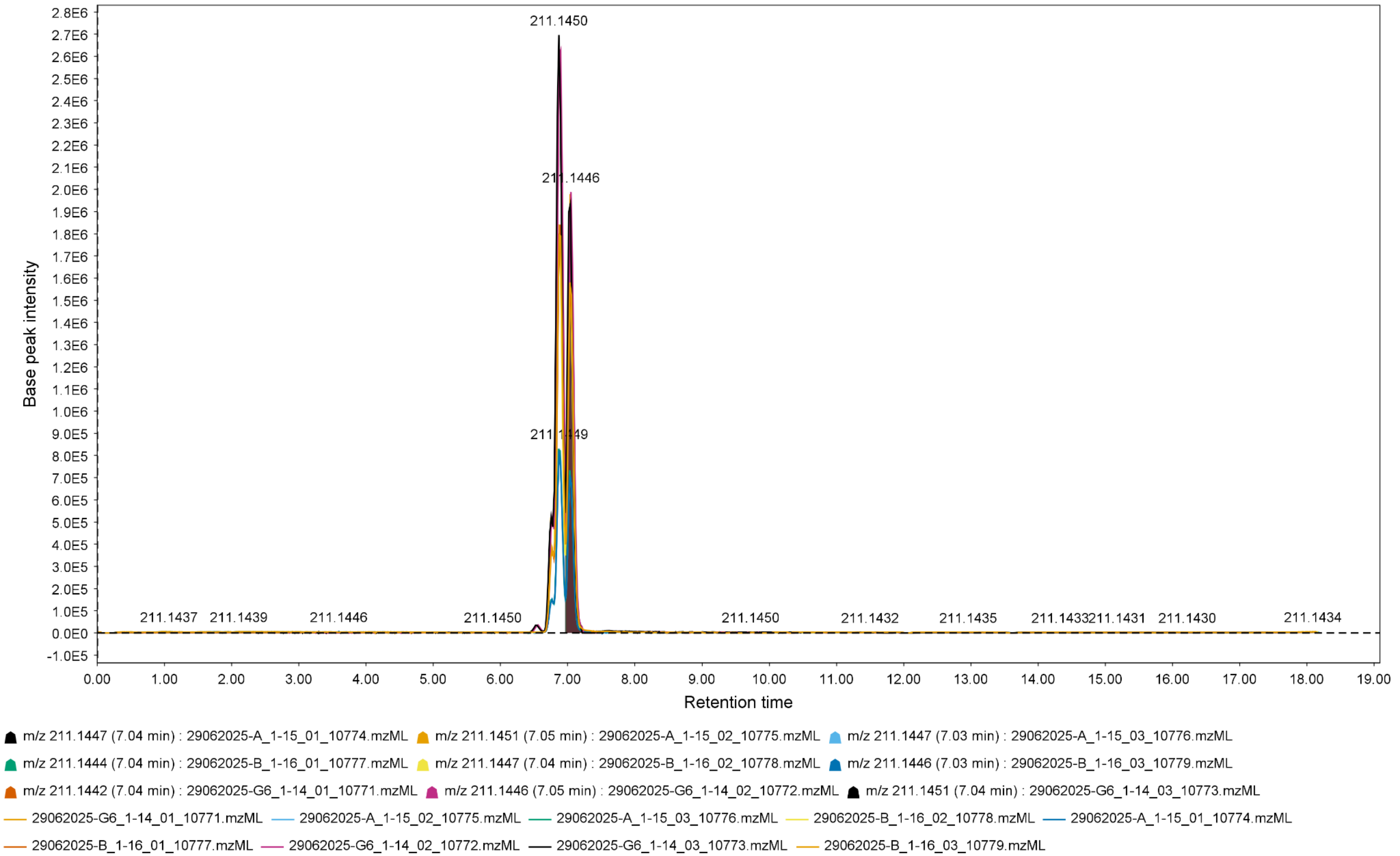

Figure S17. Chromatogram obtained through UPLC corresponding to the Cyclo-(D-Trp-L-Pro) signal. m/z 284.14. Detected on 7.36.

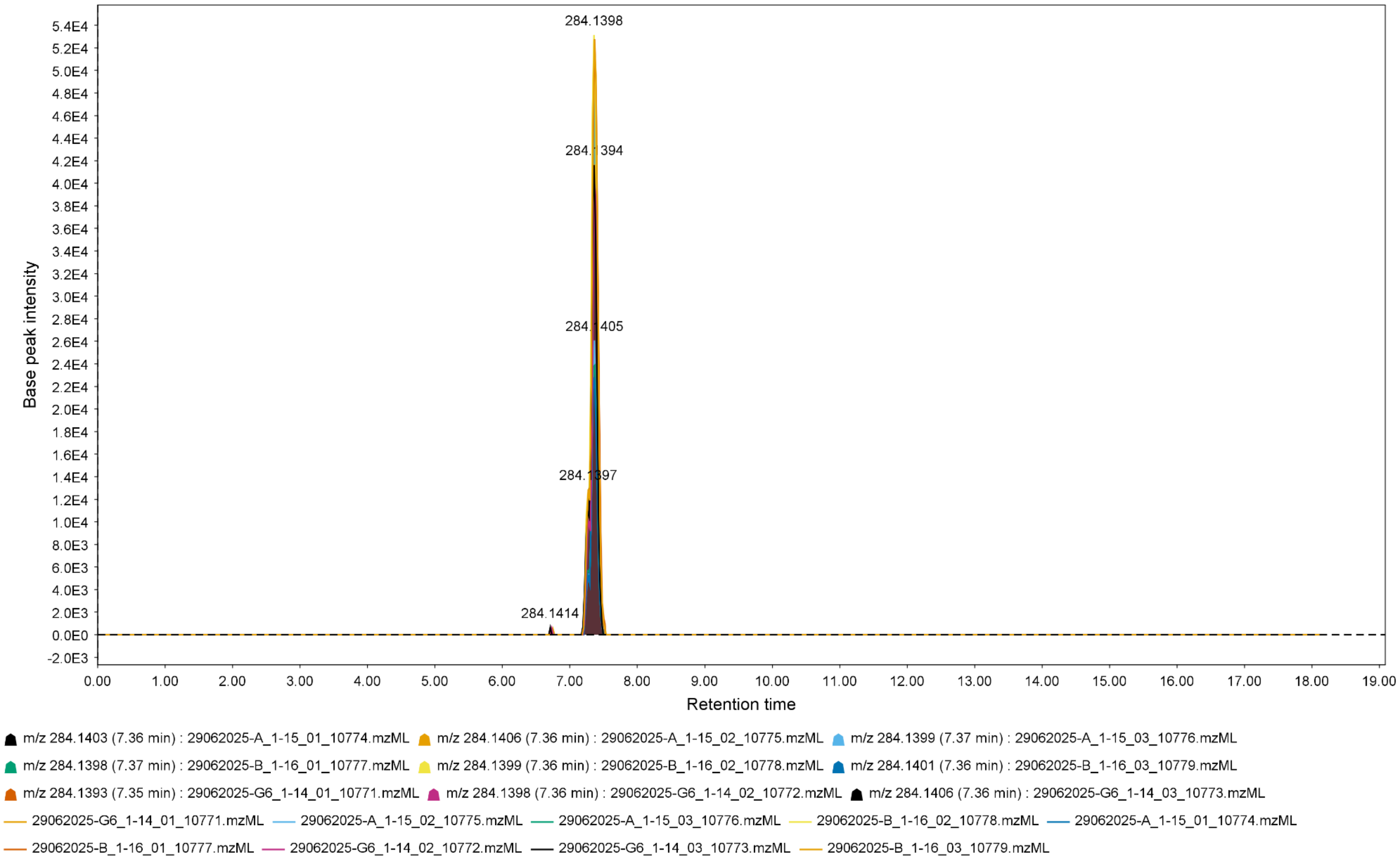

Figure S18. Chromatogram obtained through UPLC corresponding to the Cyclo(L-Phe-D-Pro) signal. m/z 245.12. Detected on 7.45 min.

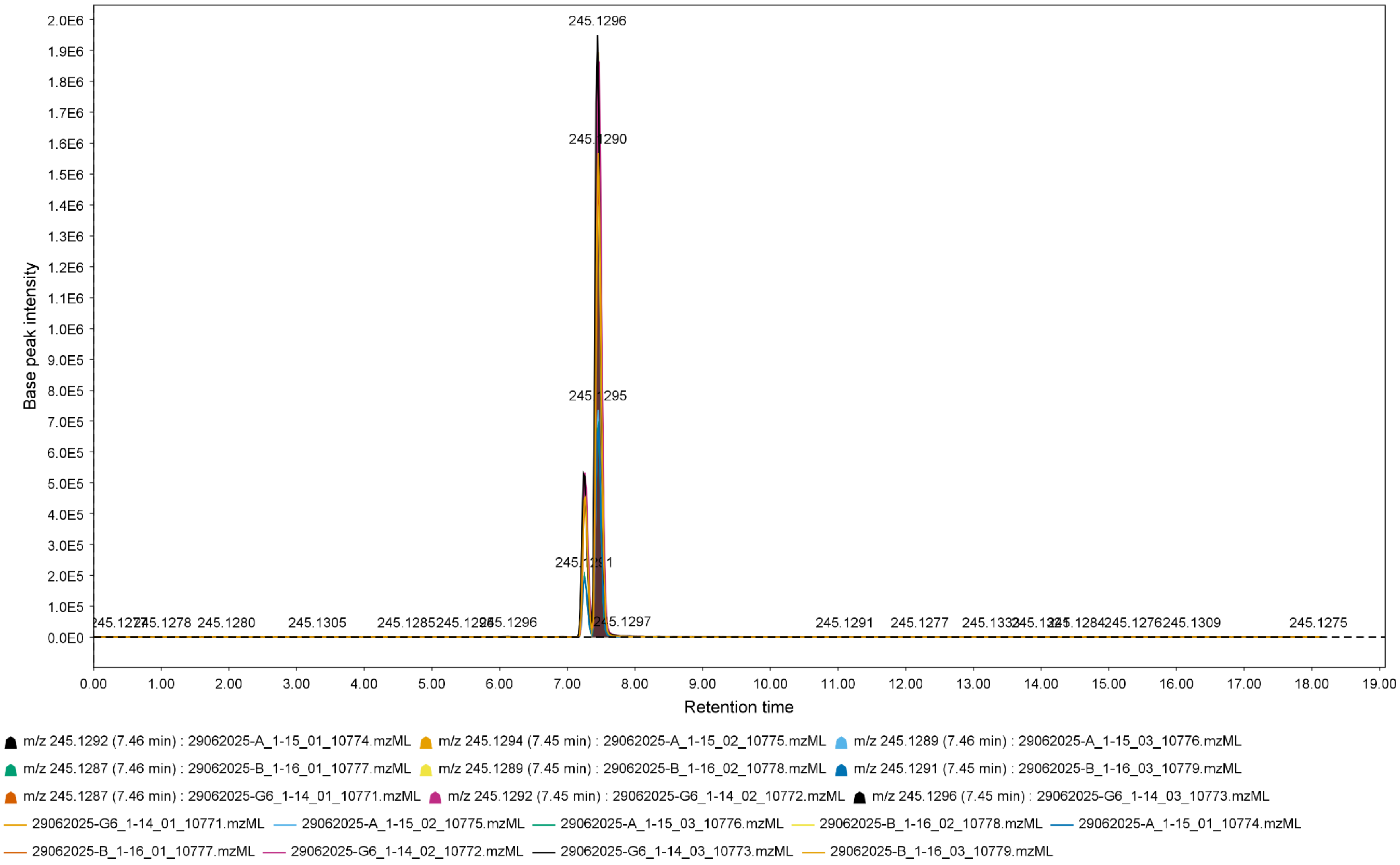

Figure S19. Chromatogram obtained through UPLC corresponding to the Indole-3-acetic acid signal. m/z 176.07. Detected on 7.66 min.

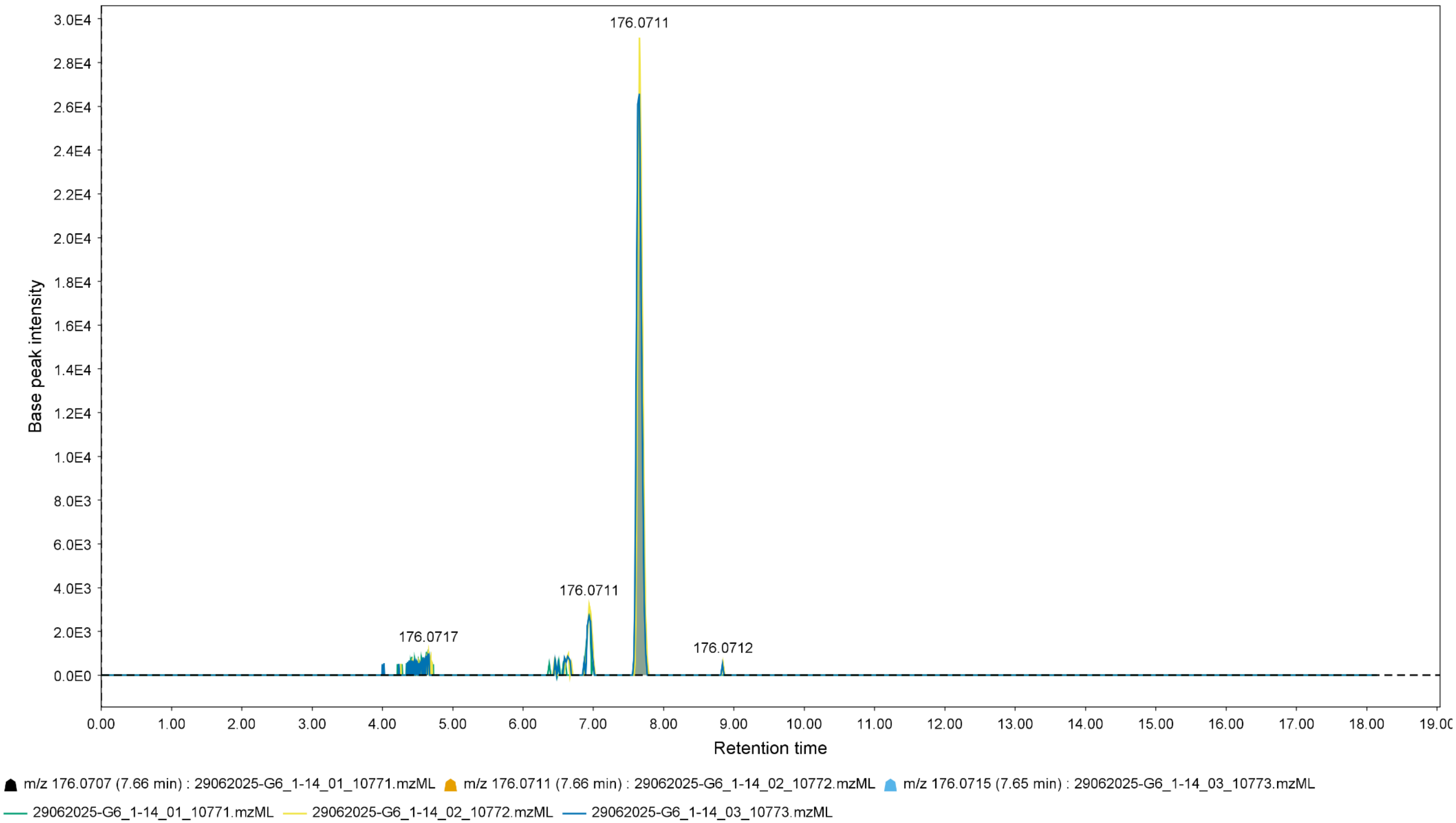

Figure S20. Chromatogram obtained through UPLC corresponding to the Cyclo-(Val-Leu) signal. m/z 213.16. Detected on 8.09 min.

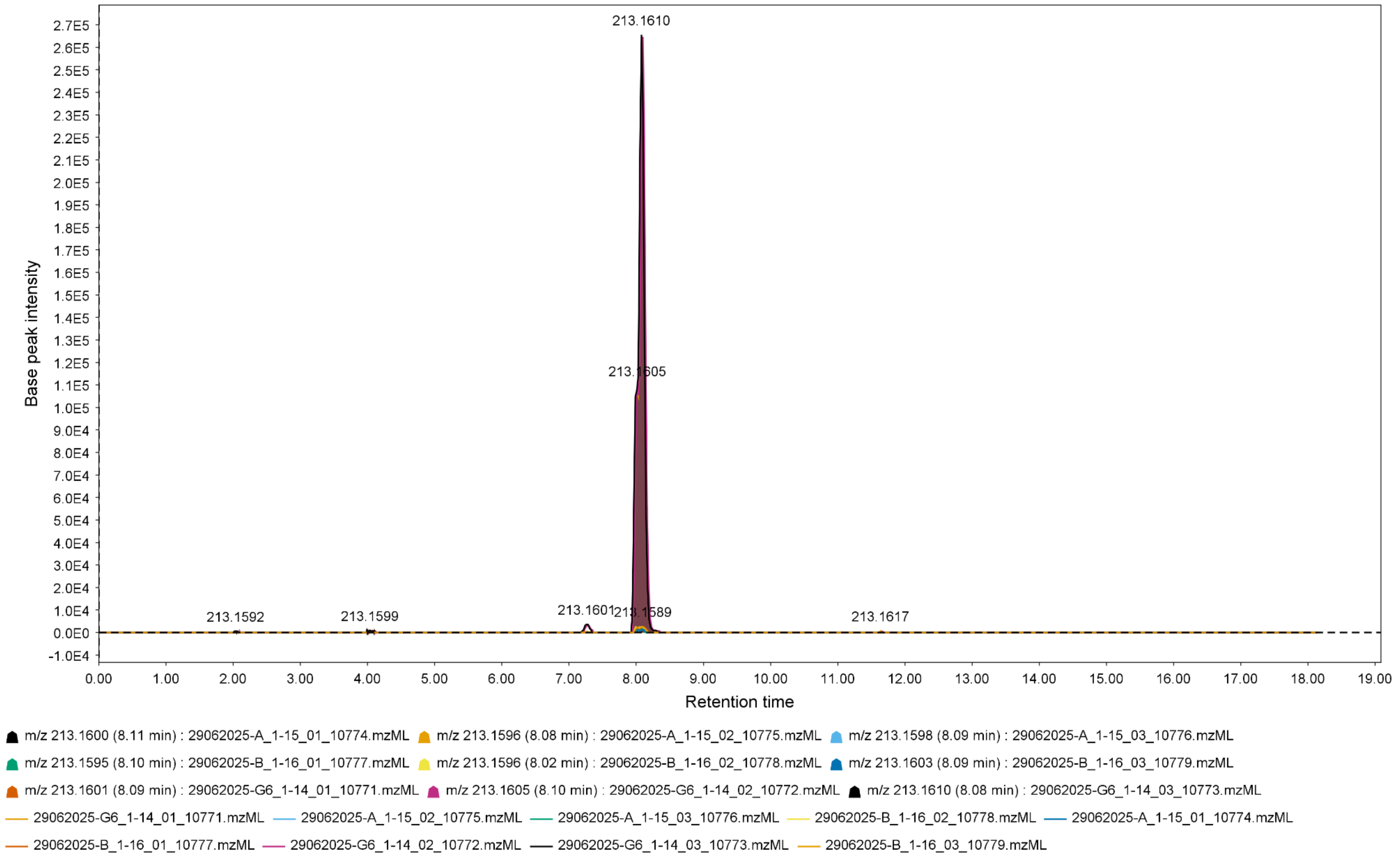

Figure S21. Chromatogram obtained through UPLC corresponding to the Loliolide signal. m/z 197.12. Detected on 8.27 min.

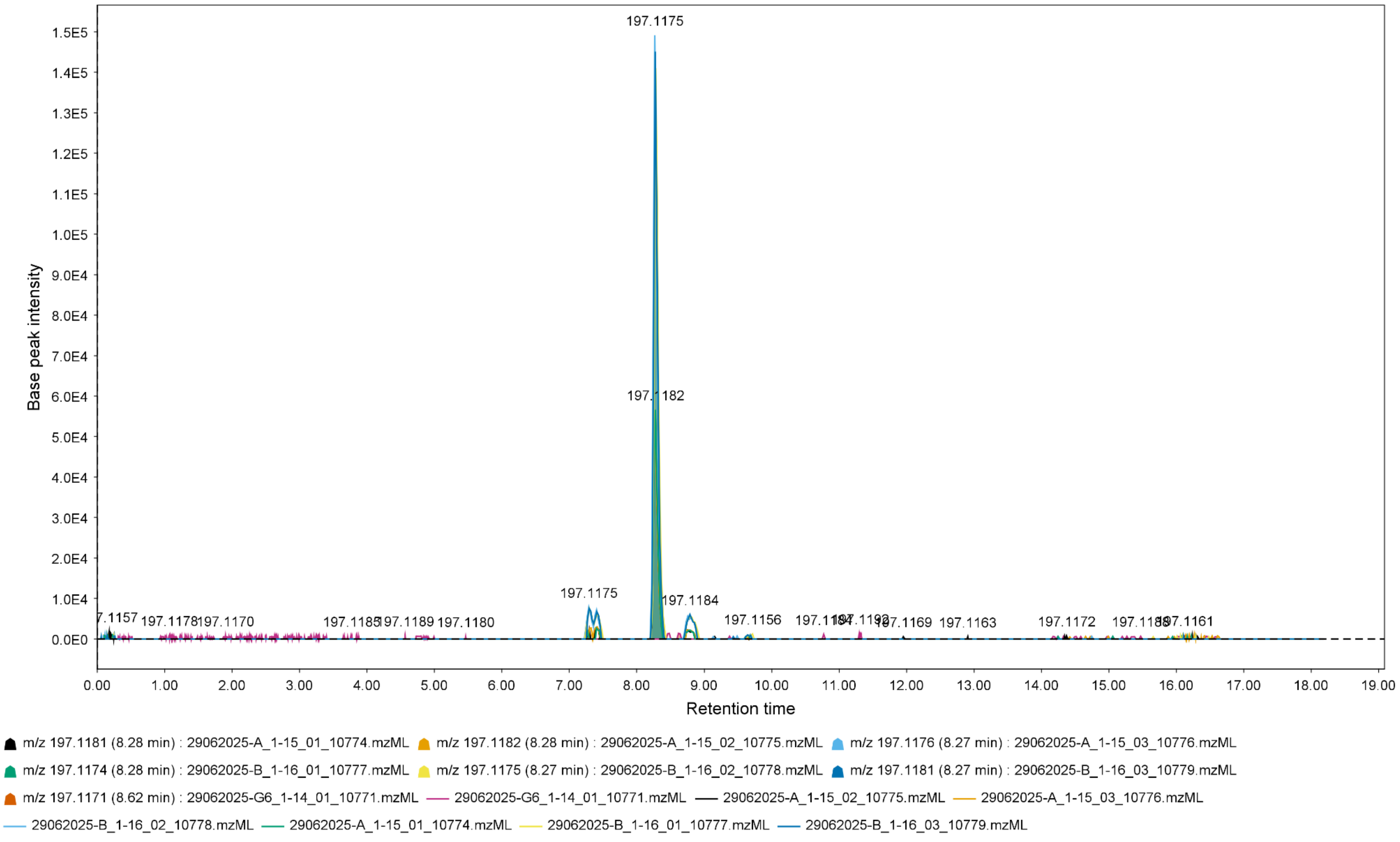

Figure S22. Chromatogram obtained through UPLC corresponding to the Cyclo-(Val-Phe) signal. m/z 247.14. Detected on 8.30 min.

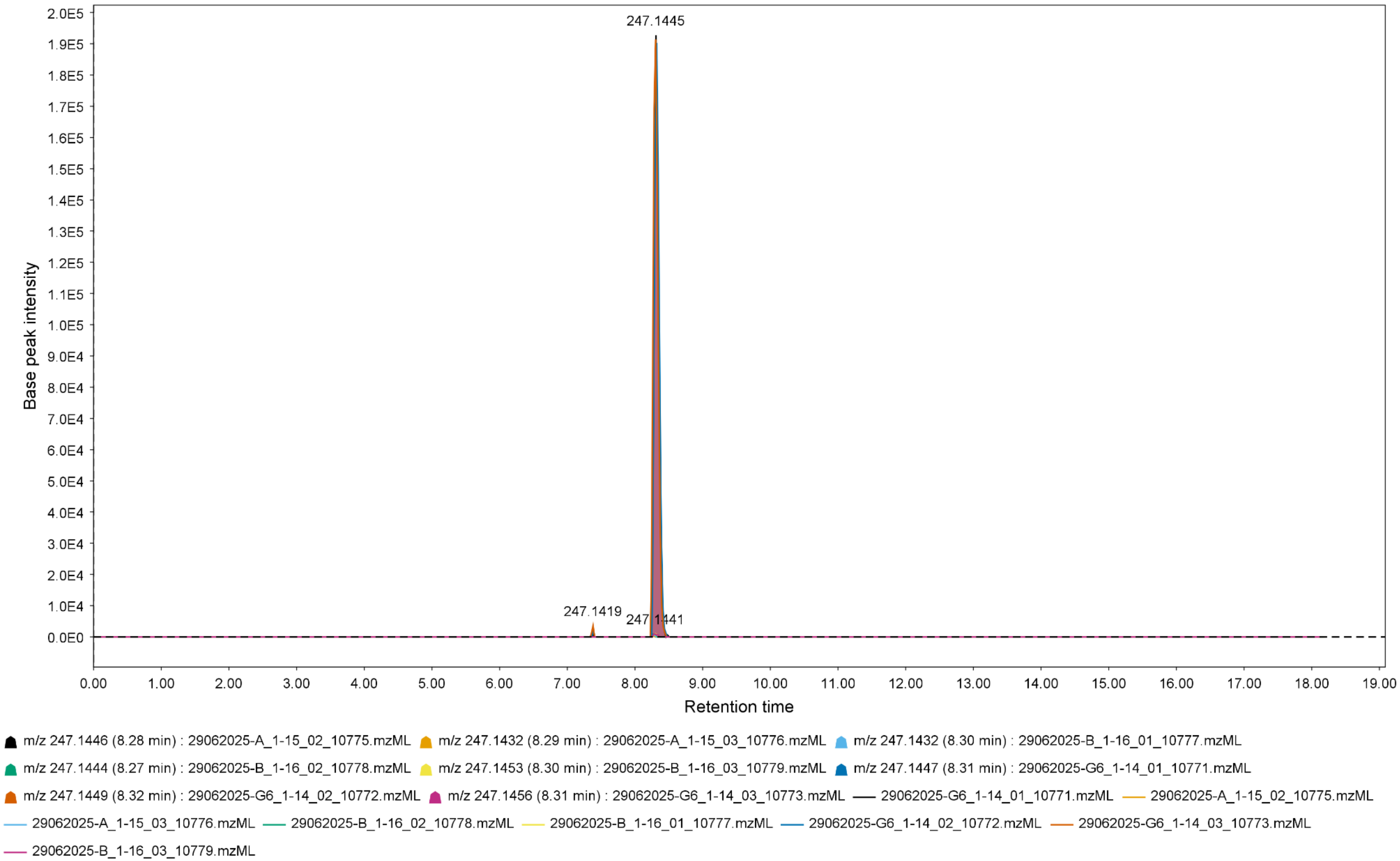

Figure S23. Chromatogram obtained through UPLC corresponding to the (2-(4,4-dimethyl-4,5-dihydrooxazol-2-yl)-5-fluorophenyl)(phenyl)methanol signal. m/z 300.13. Detected on 8.51 min.

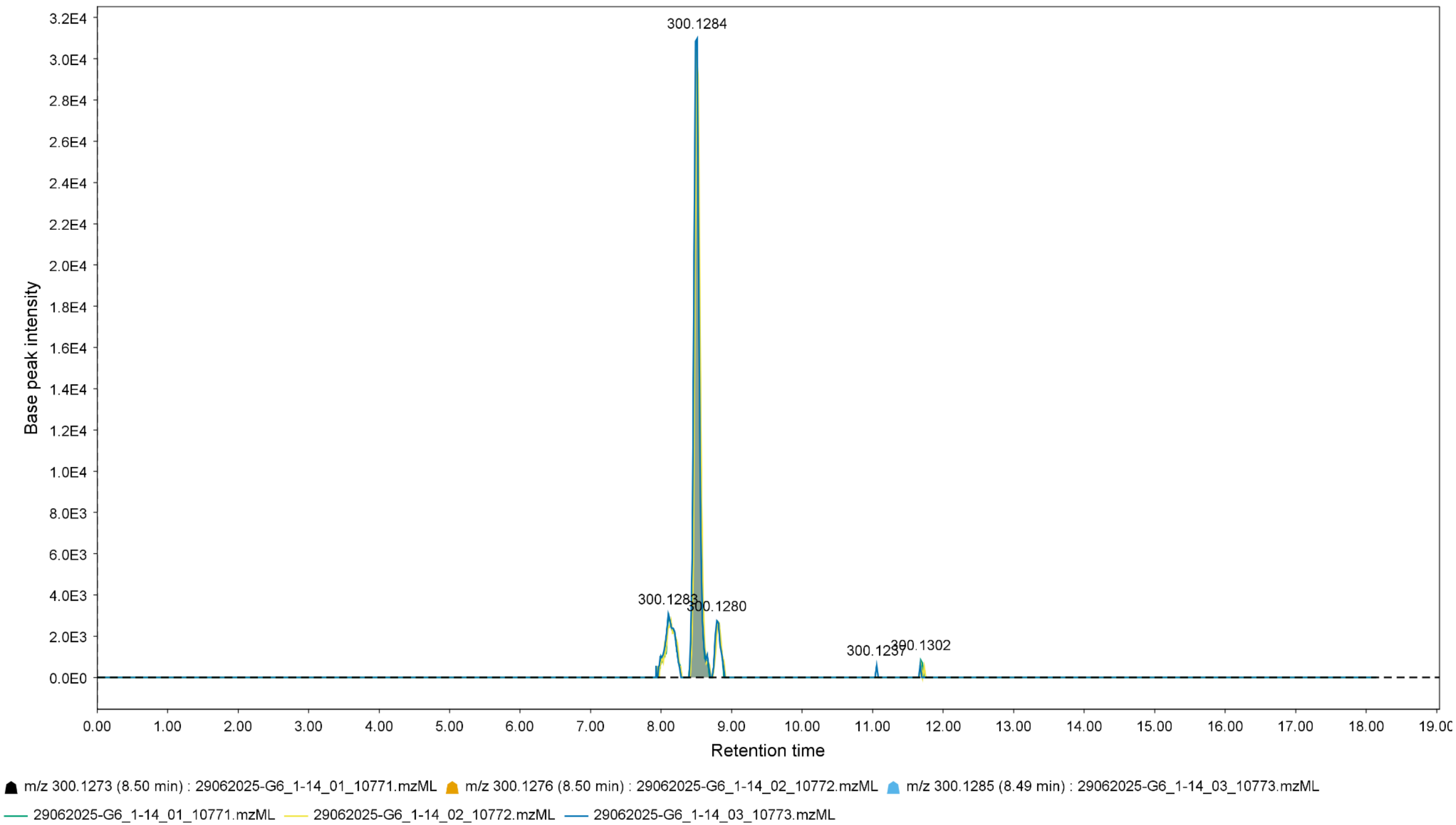

Figure S24. Chromatogram obtained through UPLC corresponding to the Cyclo-(Leu-Leu) signal. m/z 227.18. Detected on 8.78 min.

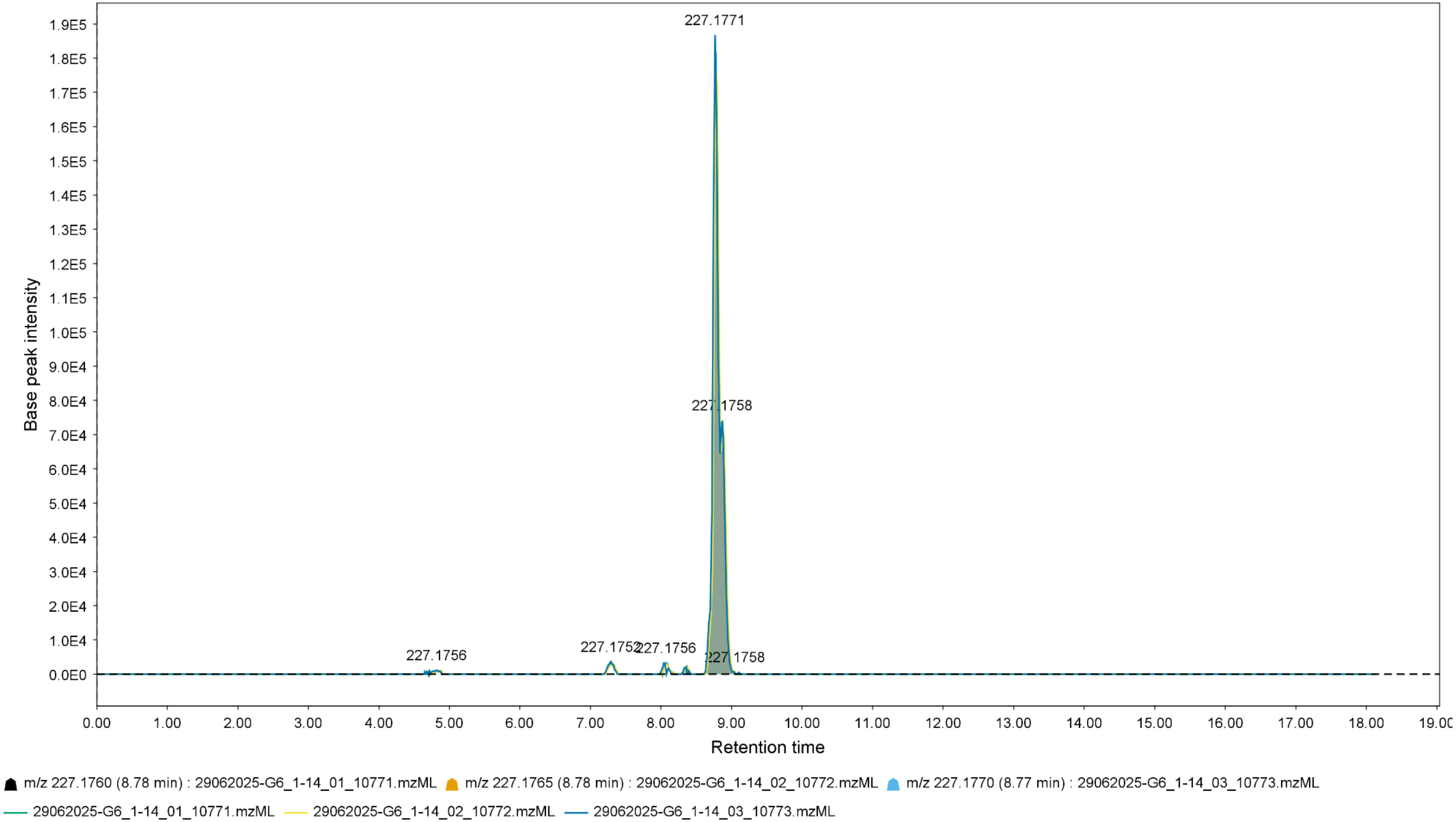

Figure S25. Chromatogram obtained through UPLC corresponding to the Cyclo-(Leu-Phe) signal. m/z 261.16. Detected on 8.84 min.

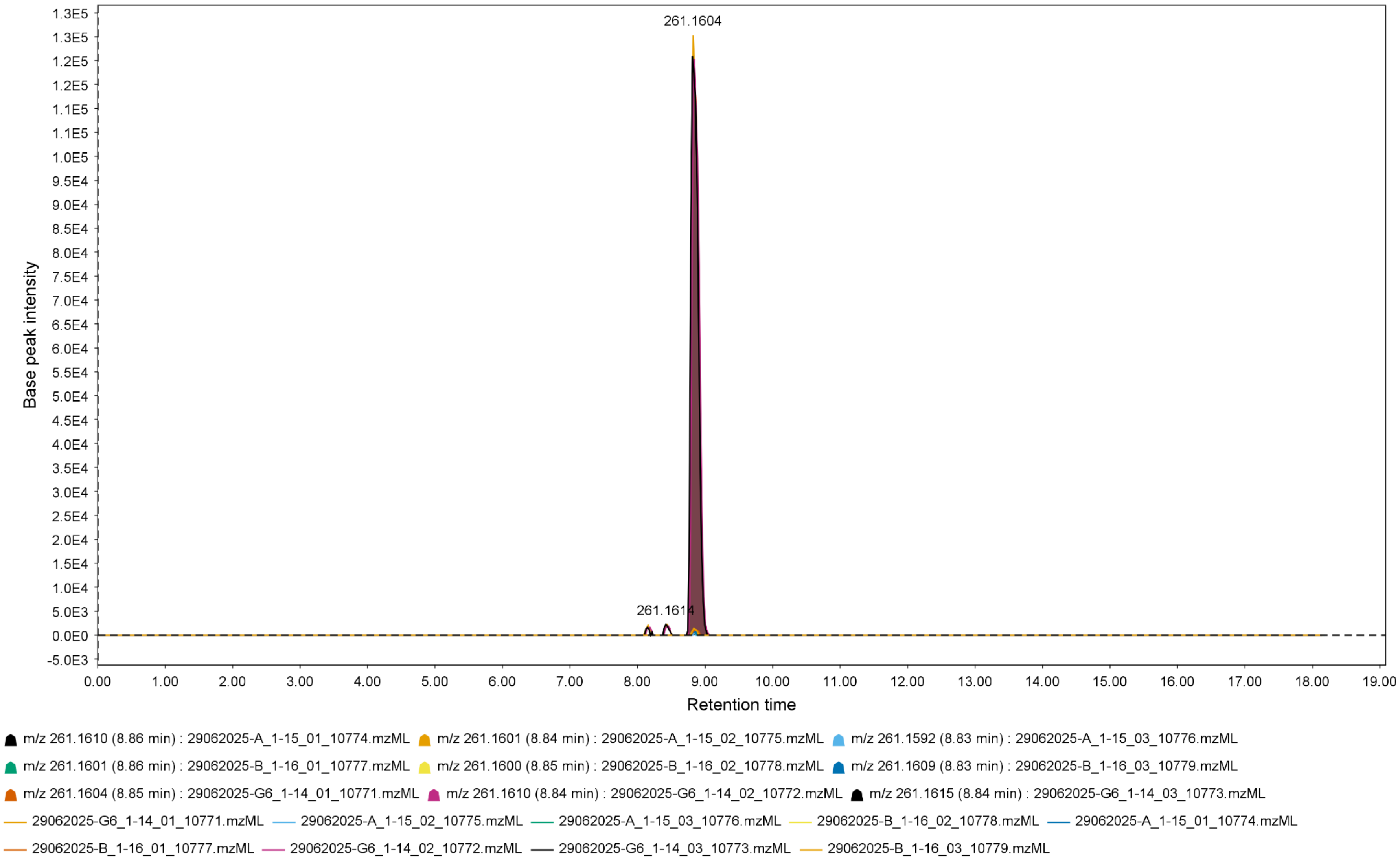

Figure S26. Chromatogram obtained through UPLC corresponding to the N-(3-Hydroxytetradecanoyl)-DL-homoserine lactone signal. m/z 328.25. Detected on 9.94 min.

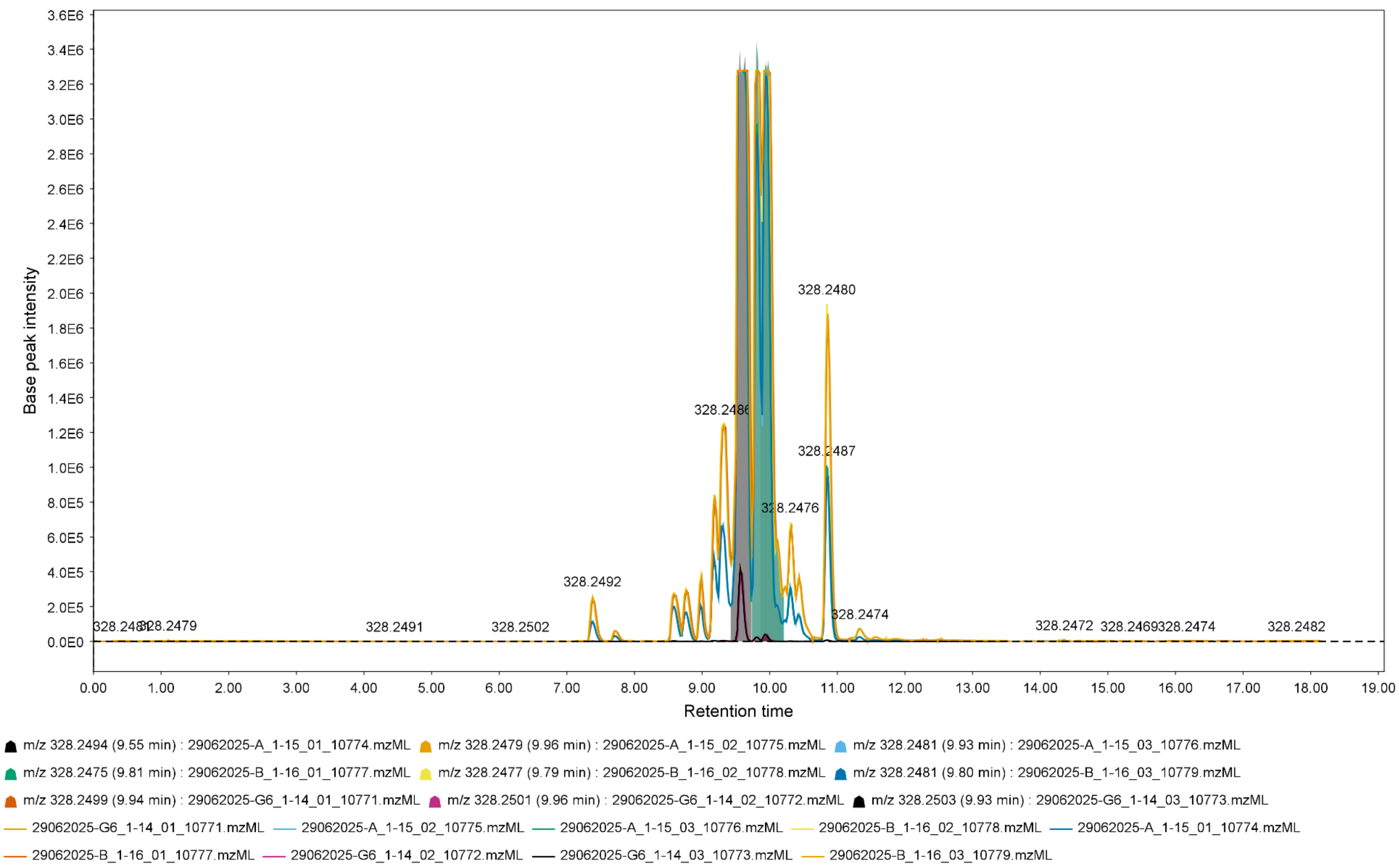

Figure S27. Chromatogram obtained through UPLC corresponding to the (3S)-5-[(1S,8aR)-2,5,5,8a-tetramethyl-4-oxo-4a,6,7,8-tetrahydro-1H-naphthalen-1-yl]-3-methylpentanoic acid signal. m/z 321.24. Detected on 10.77 min.

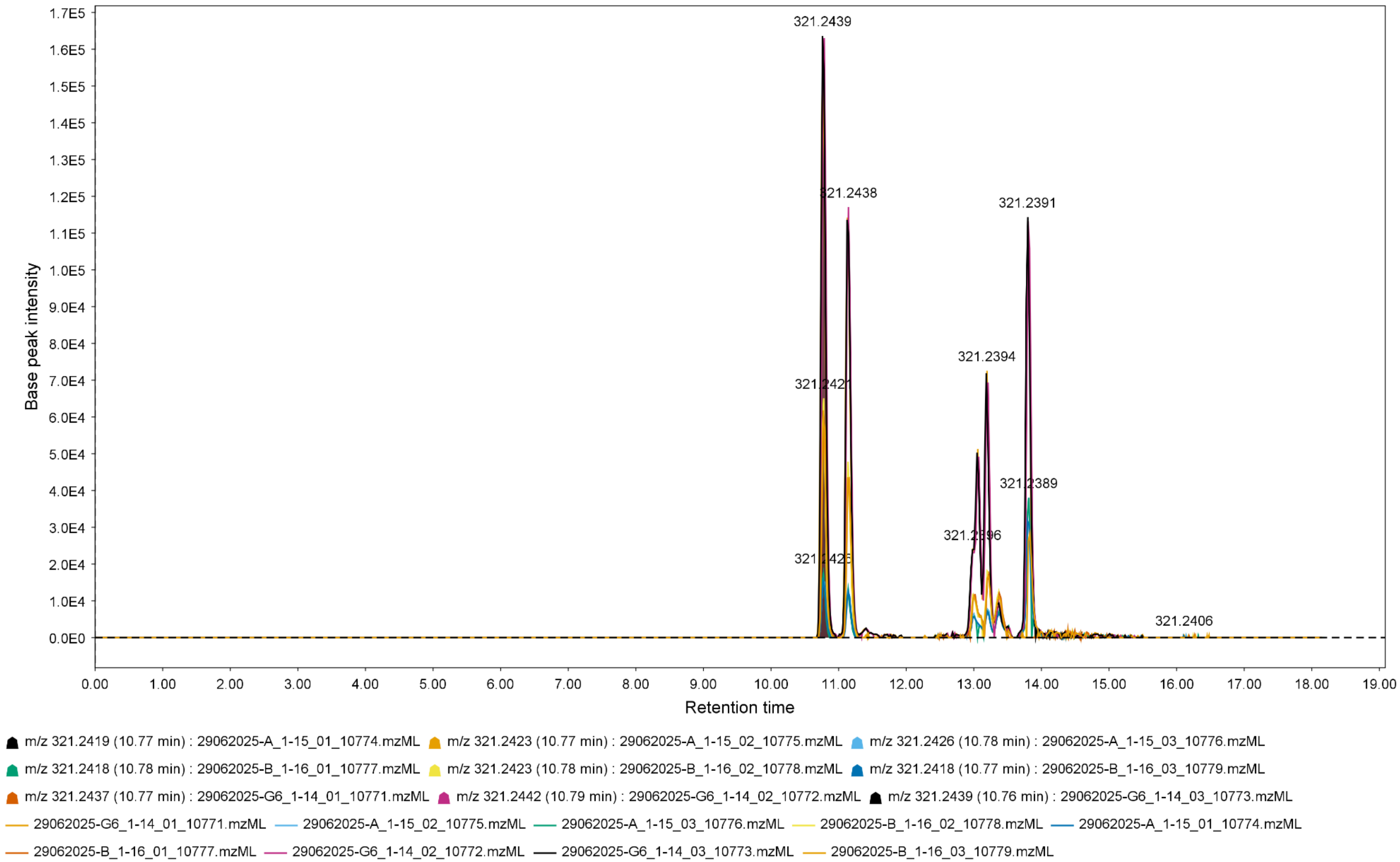

Figure S28. Chromatogram obtained through UPLC corresponding to the Fa 18:3+1O signal. m/z 295.23. Detected on 10.84 min.

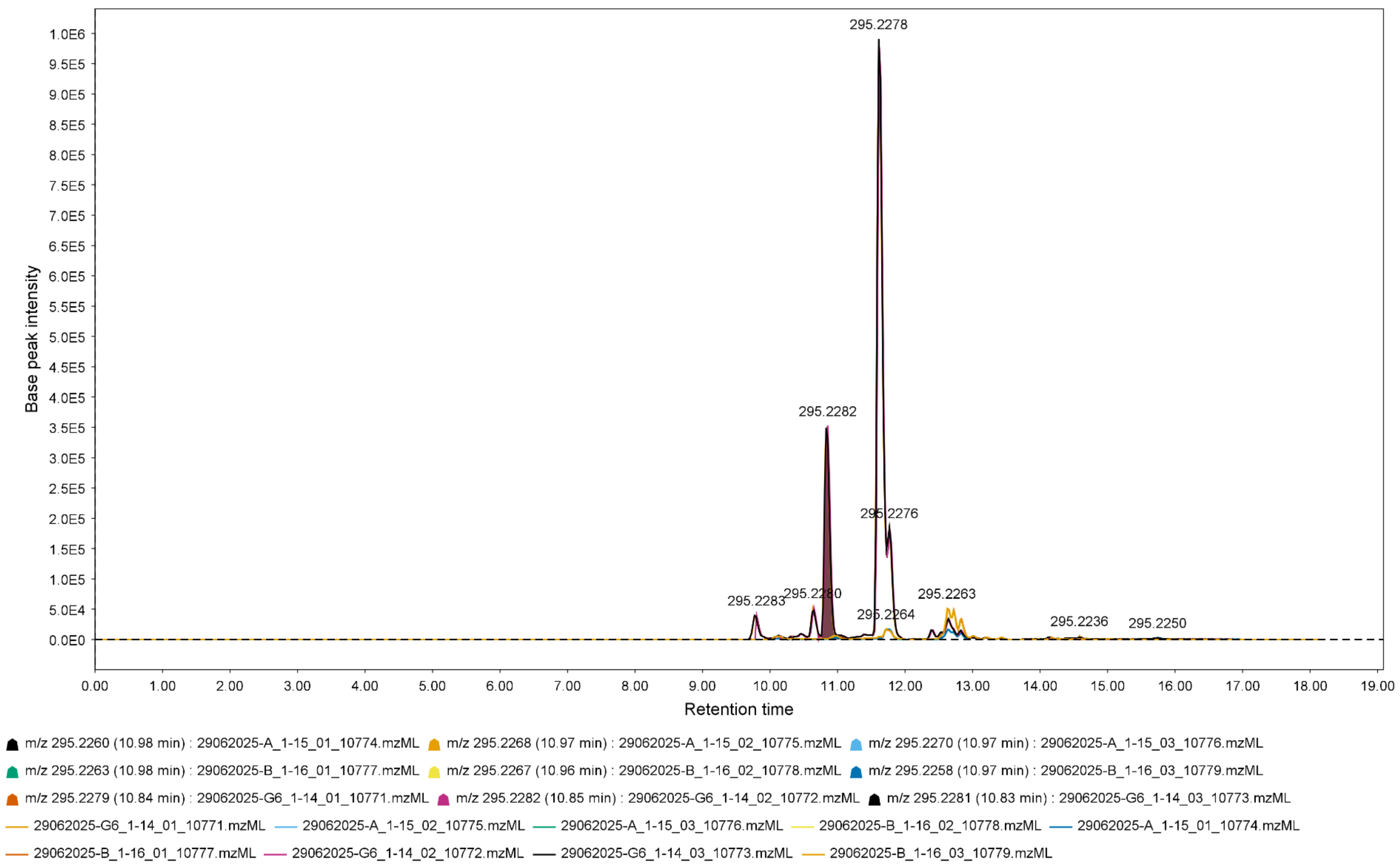

Figure S29. Chromatogram obtained through UPLC corresponding to the (10E,15E)-9,12,13-trihydroxy-10,15-octadecadienoic acid signal. m/z 293.21. Detected on 11.03 min.

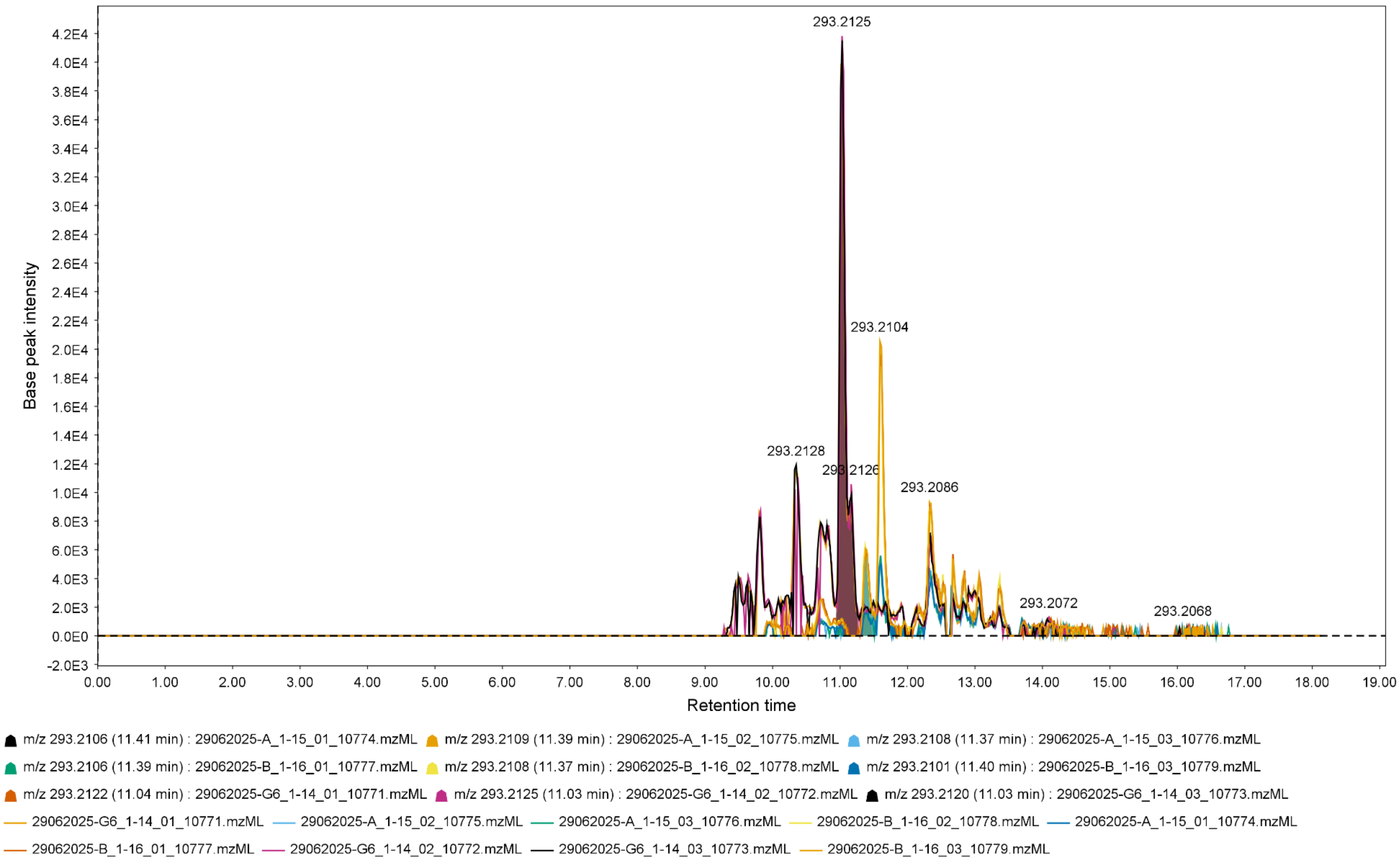

Figure S30. Chromatogram obtained through UPLC corresponding to the 5-[(8aS)-2,5,5,8a-tetramethyl-3-oxo-4a,6,7,8-tetrahydro-4H-naphthalen-1-yl]-3-methylpentanoic acid signal. m/z 321.24. Detected on 11.14 min.

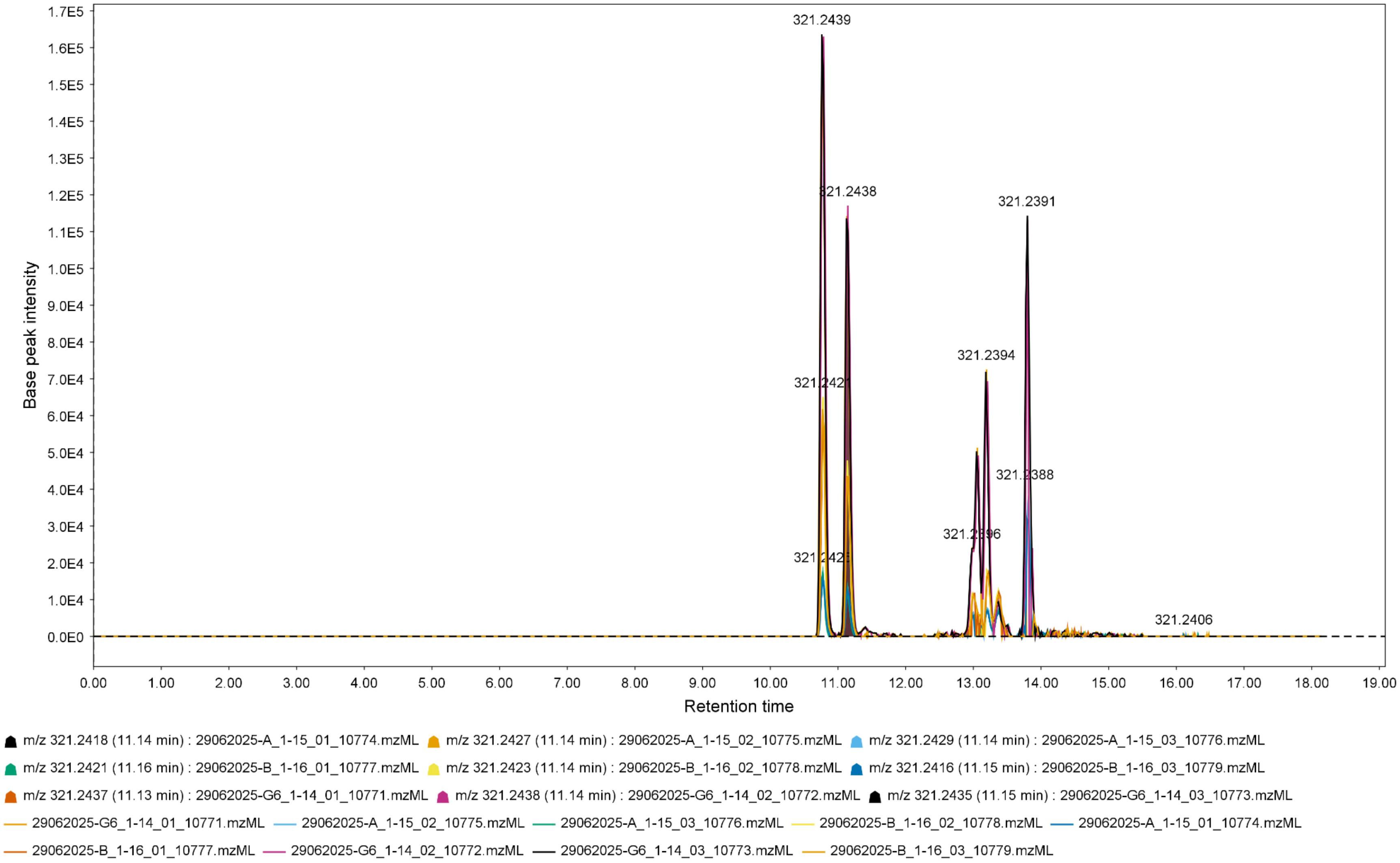

Figure S31. Chromatogram obtained through UPLC corresponding to the Linoleic acid signal. m/z 281.24. Detected on 9.81 min.

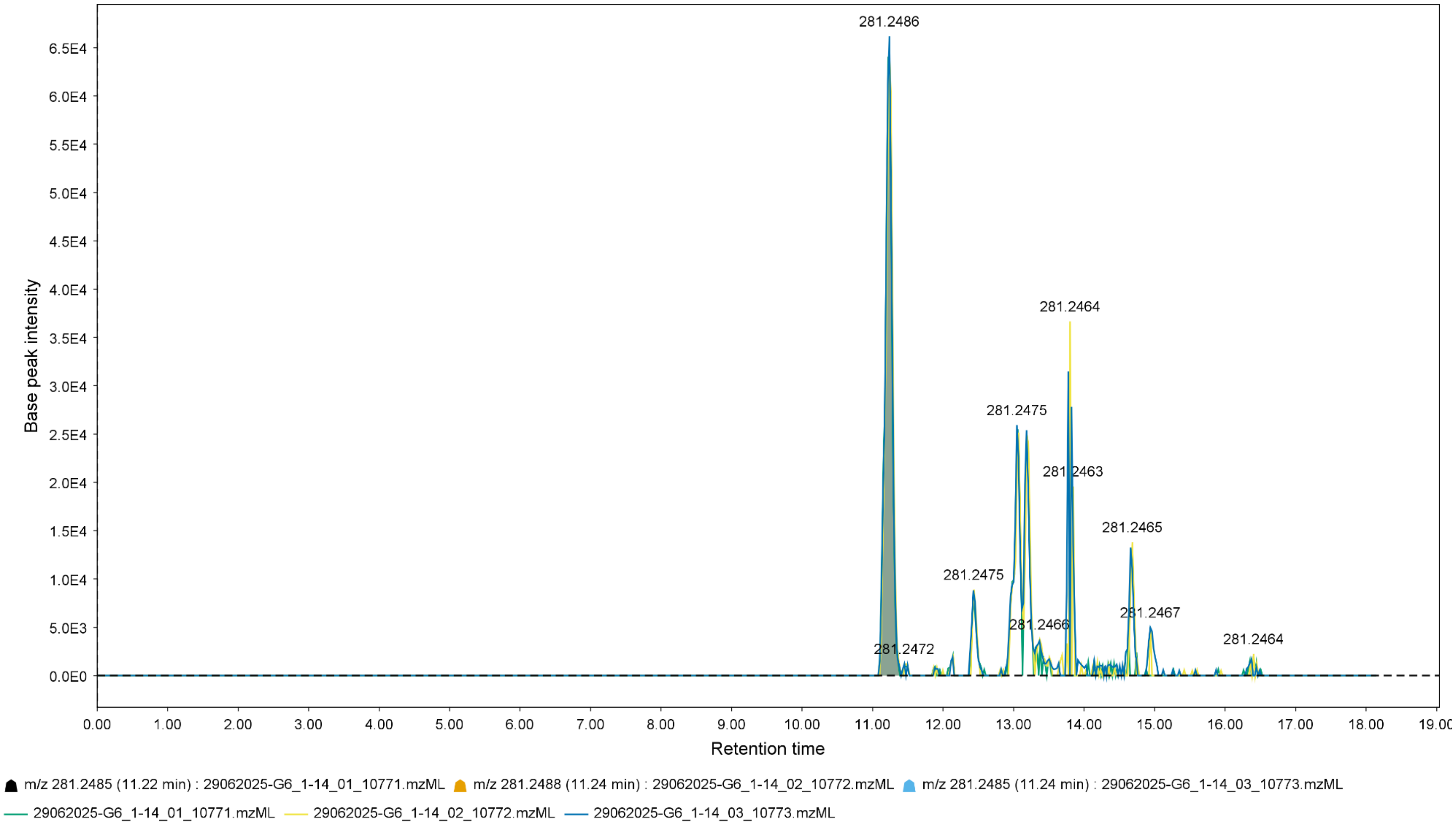

Figure S32. Chromatogram obtained through UPLC corresponding to the Gamma-linolenic acid signal. m/z 279.23. Detected on 12.74 min.

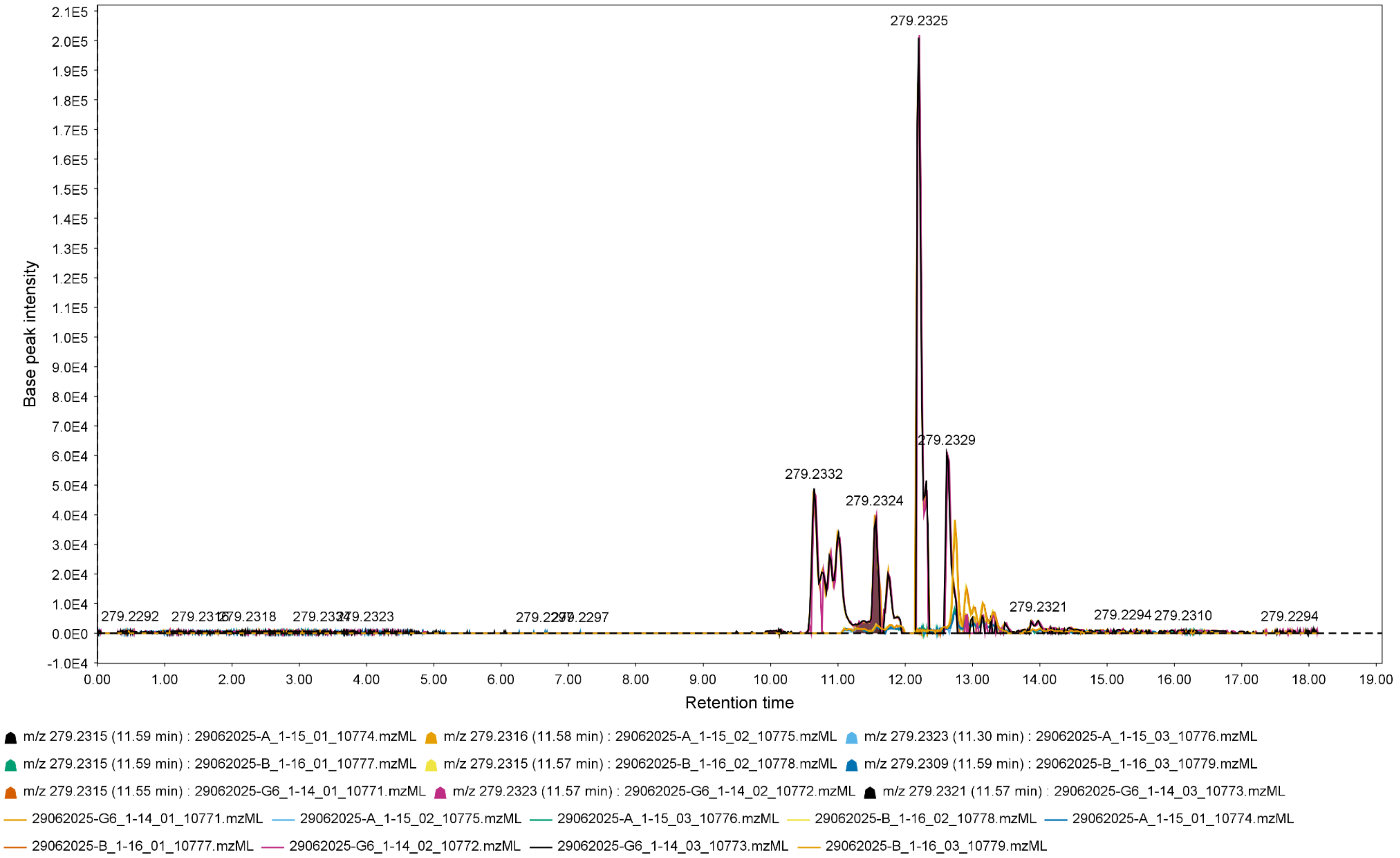

Figure S33. Chromatogram obtained through UPLC corresponding to the D-sphingosine signal. m/z 300.29. Detected on 14.48 min.

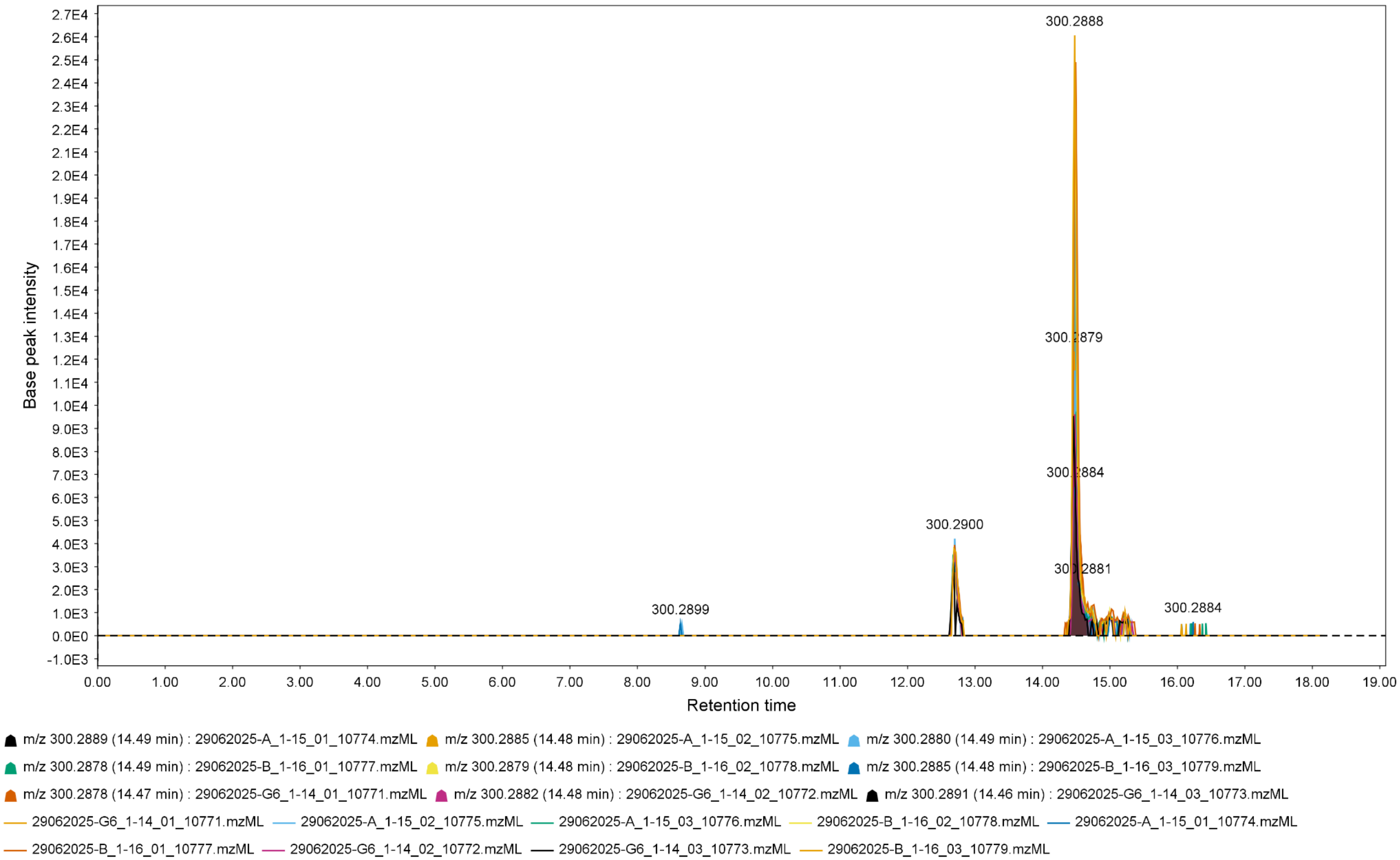

Figure S34. Chromatogram obtained through UPLC corresponding to the Ceratodictyol signal. m/z 331.28. Detected on 14.81 min.

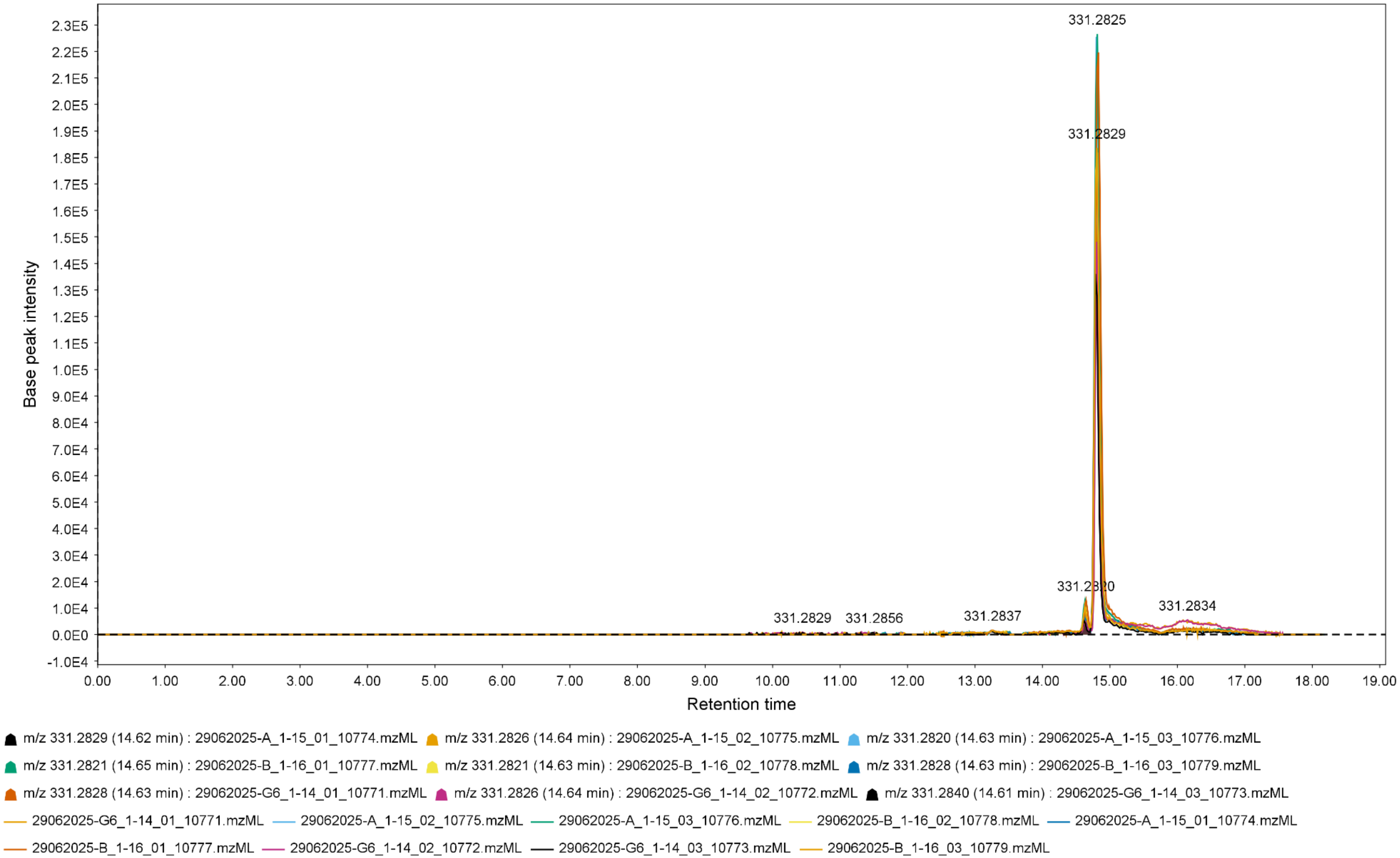

Supplementary Material

Fragmentation spectra of the features presented on Table 1.

Figure S1 Fragmentation spectrum (MS/MS) corresponding to the Pyridoxine molecule [M-H+], m/z 170.08. Detected on 0.73 min.

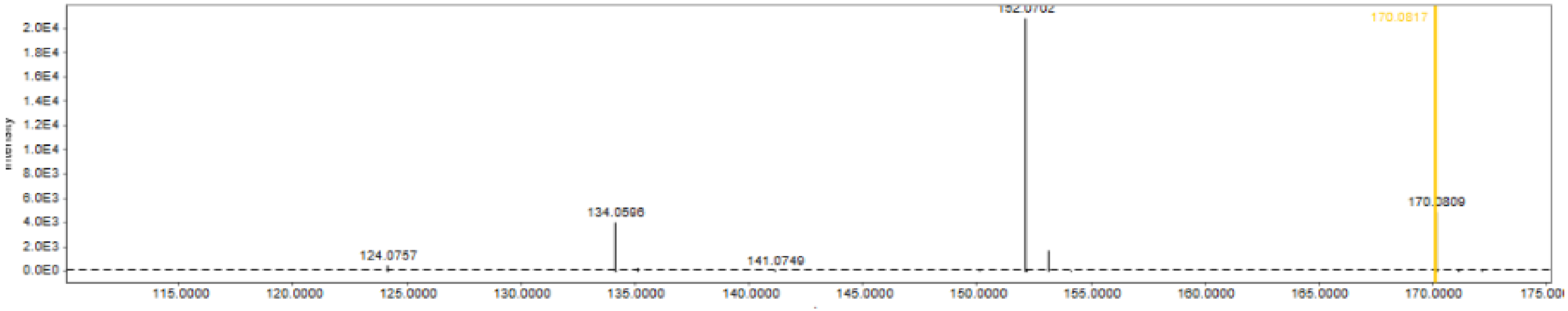

Figure S2. Fragmentation spectrum (MS/MS) corresponding to the Glutamate molecule [M-H+], m/z 148.06. Detected on 0.77 min.

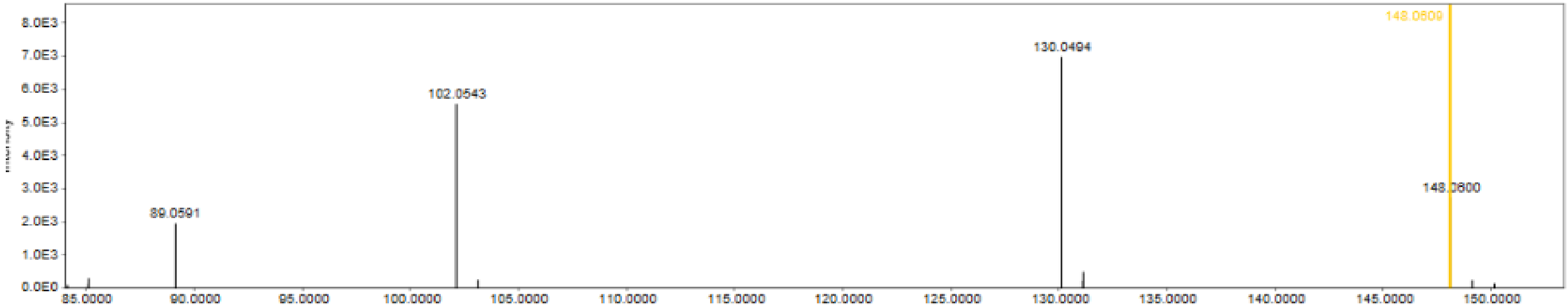

Figure S3. Fragmentation spectrum (MS/MS) corresponding to the Xanthine molecule [M-H+], m/z 153.04. Detected on 1.58 min.

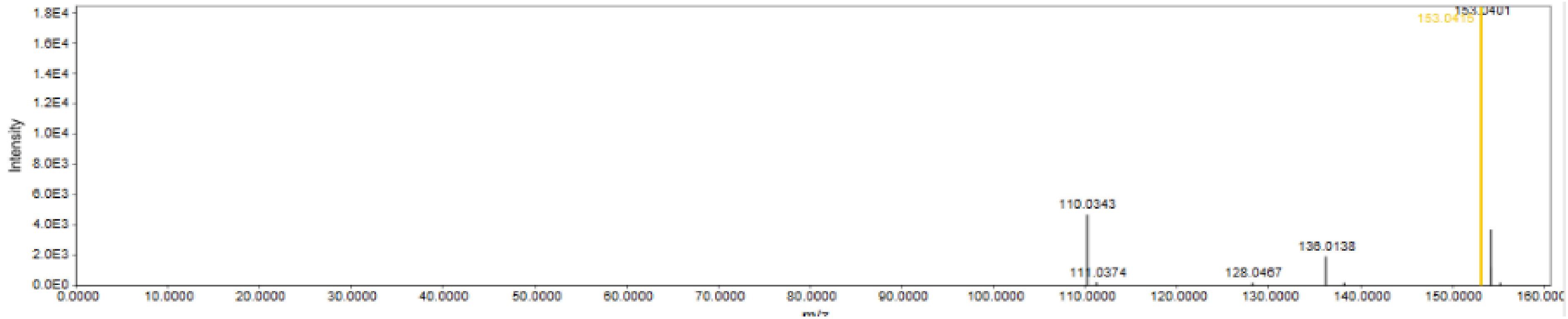

Figure S4. Fragmentation spectrum (MS/MS) corresponding to the Cyclo-(Ala-4-hydroxy-Pro) molecule [M-H+], m/z 185.09. Detected on 1.73 min.

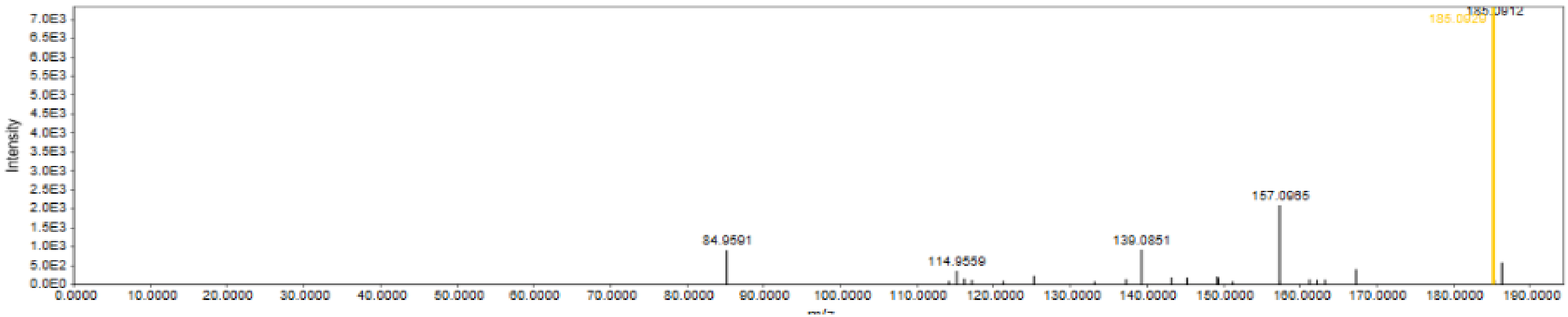

Figure S5. Fragmentation spectrum (MS/MS) corresponding to the Thymine molecule [M-H+], m/z 127.05. Detected on 1.82 min.

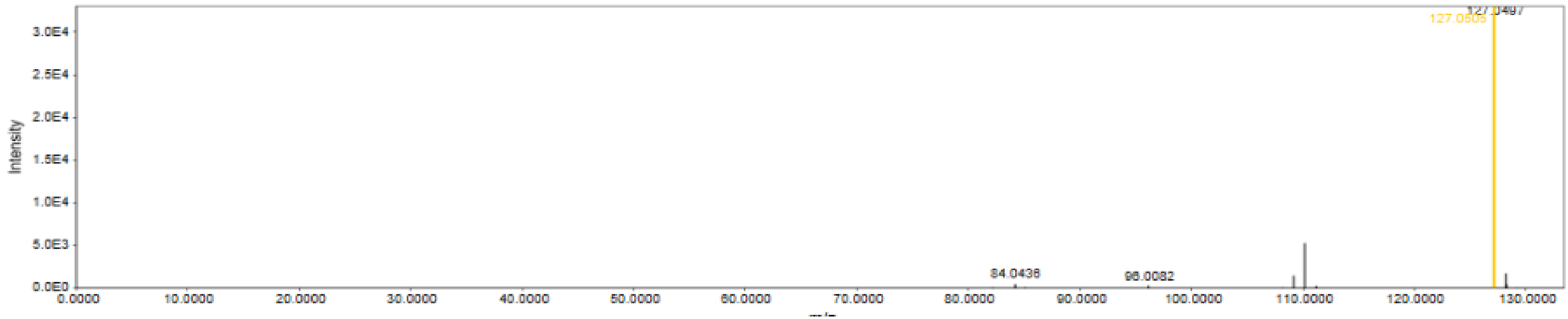

Figure S6. Fragmentation spectrum (MS/MS) corresponding to the Cyclo-(Pro-Gly) molecule [M-H+], m/z 155.08. Detected on 2.02 min.

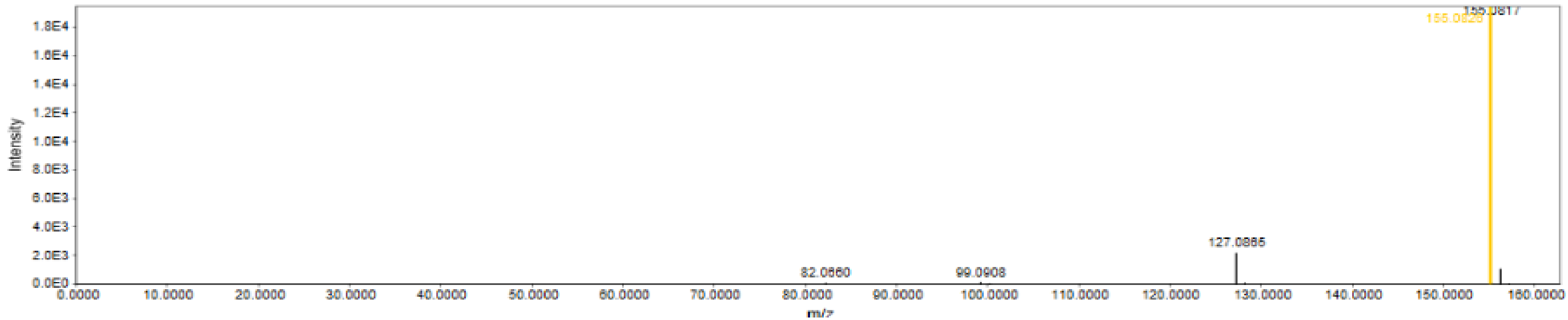

Figure S7. Fragmentation spectrum (MS/MS) corresponding to the Cyclo-(Th-Pro) molecule [M-H+], m/z 199.11. Detected on 1.86 min.

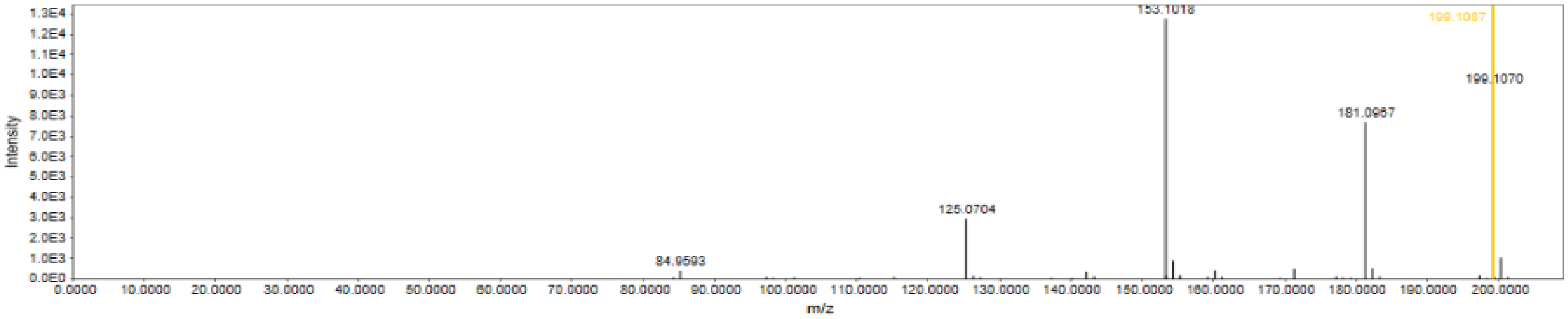

Figure S8. Fragmentation spectrum (MS/MS) corresponding to the Paeonol molecule [M-H+], m/z 167.07. Detected on 4.93 min.

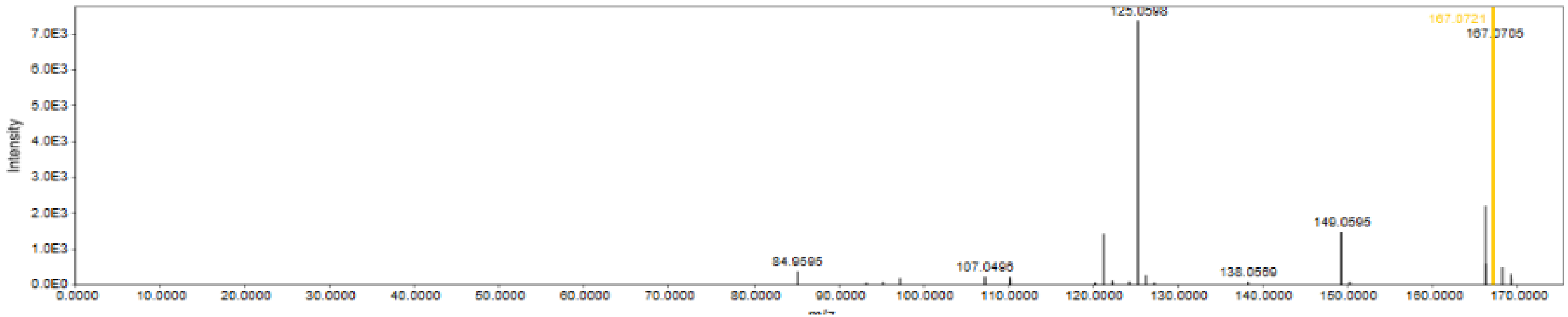

Figure S9. Fragmentation spectrum (MS/MS) corresponding to the Cyclo-(Gly-Leu) molecule [M-H+], m/z 171.11. Detected on 4.99 min.

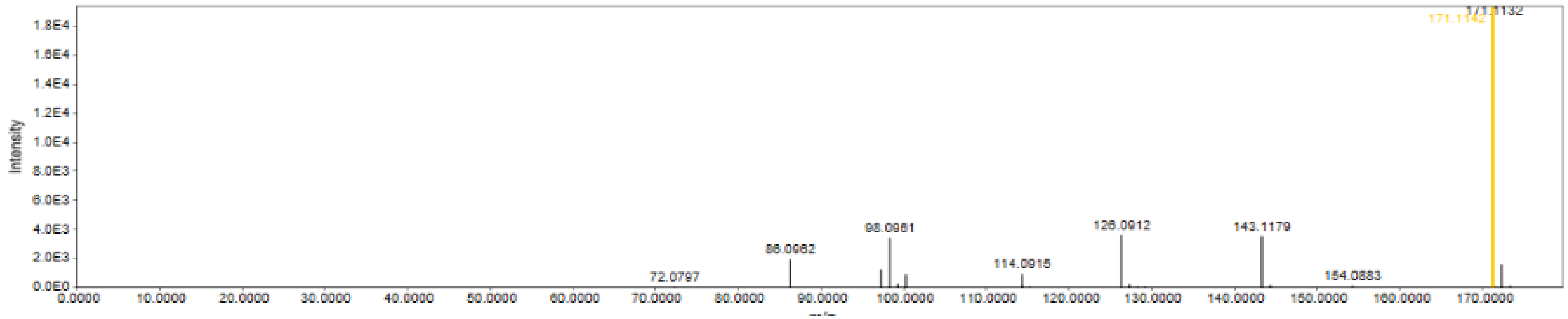

Figure S10. Fragmentation spectrum (MS/MS) corresponding to the Cyclo-(glycinyl-L-phenylalanine) molecule [M-H+], m/z 205.09. Detected on 5.62 min.

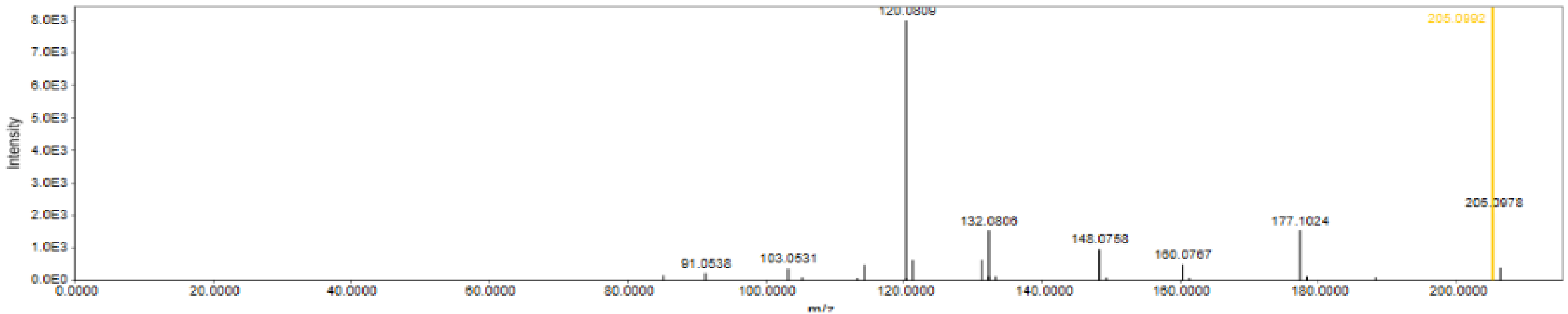

Figure S11. Fragmentation spectrum (MS/MS) corresponding to the Cyclo(L-Val-L-Pro) molecule [M-H+], m/z 197.13. Detected on 5.77 min.

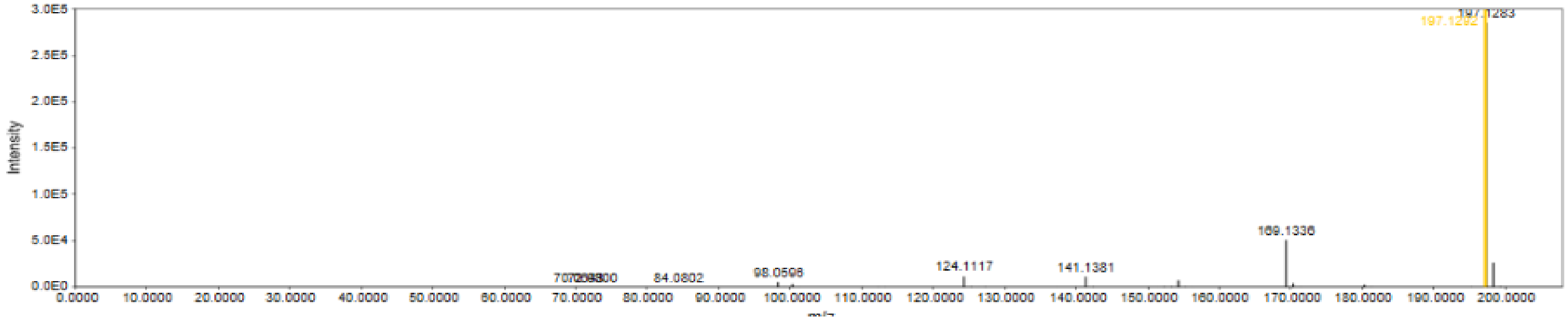

Figure S12. Fragmentation spectrum (MS/MS) corresponding to the 3-isobutylidene-hexahydro-pyrrolo[1,2-a]pyrazine-1,4-dione molecule [M-H+], m/z 209.13. Detected on 6.17 min.

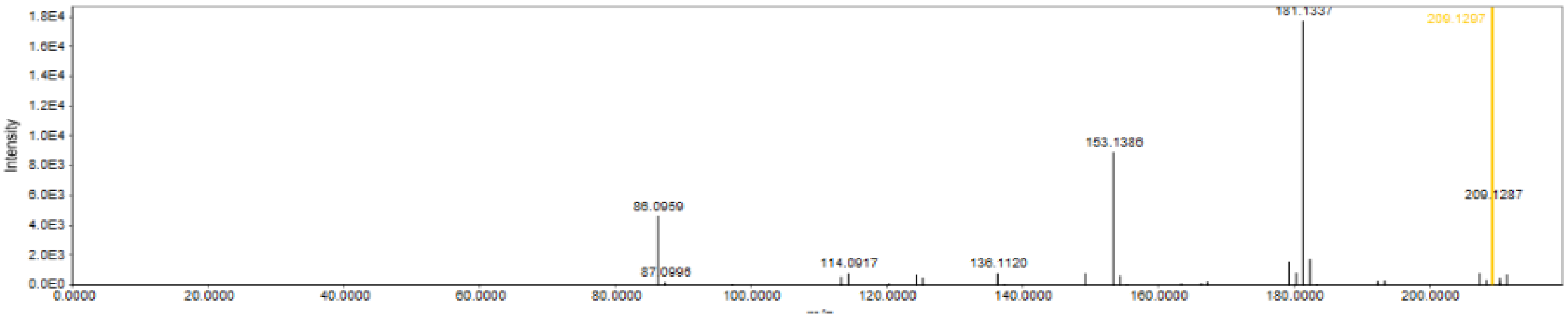

Figure S13. Fragmentation spectrum (MS/MS) corresponding to the Cyclo-(Ala-Leu) molecule [M-H+], m/z 185.13. Detected on 6.26 min.

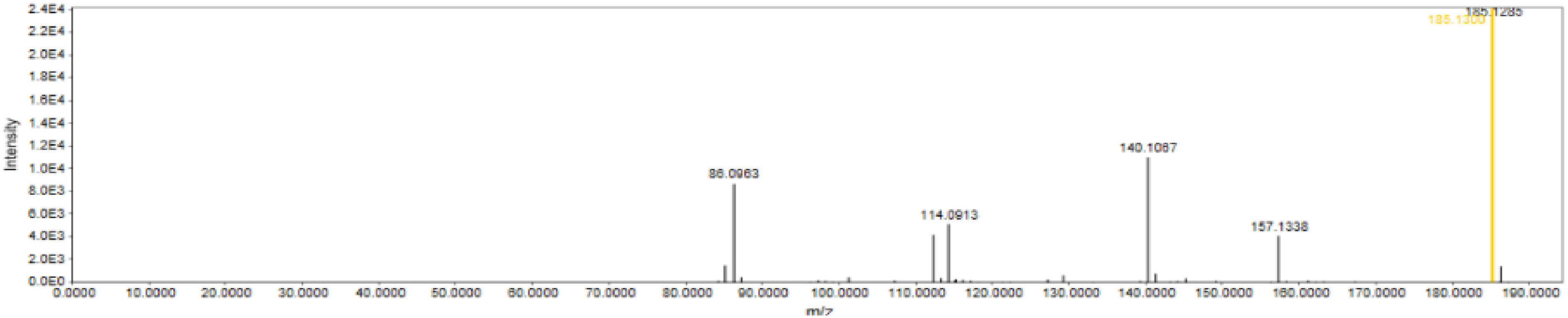

Figure S14. Fragmentation spectrum (MS/MS) corresponding to the Isoquinolone molecule [M-H+], m/z 146.06. Detected on 6.49 min.

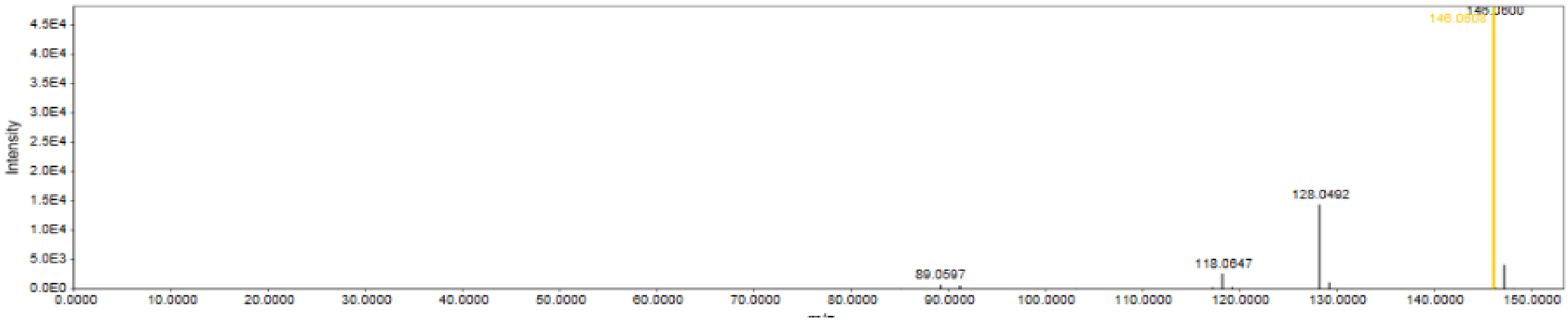

Figure S15. Fragmentation spectrum (MS/MS) corresponding to the N-acetyltyramine molecule [M-H+], m/z 180.10. Detected on 6.79 min.

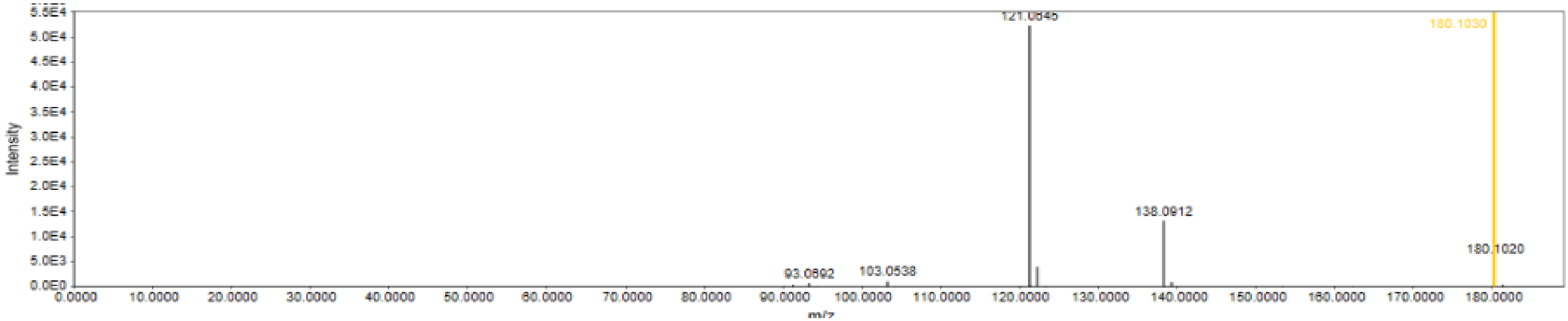

Figure S16. Fragmentation spectrum (MS/MS) corresponding to the Cyclo-(L-Leu-L-Pro) molecule [M-H+], m/z 211.14. Detected on 7.04 min.

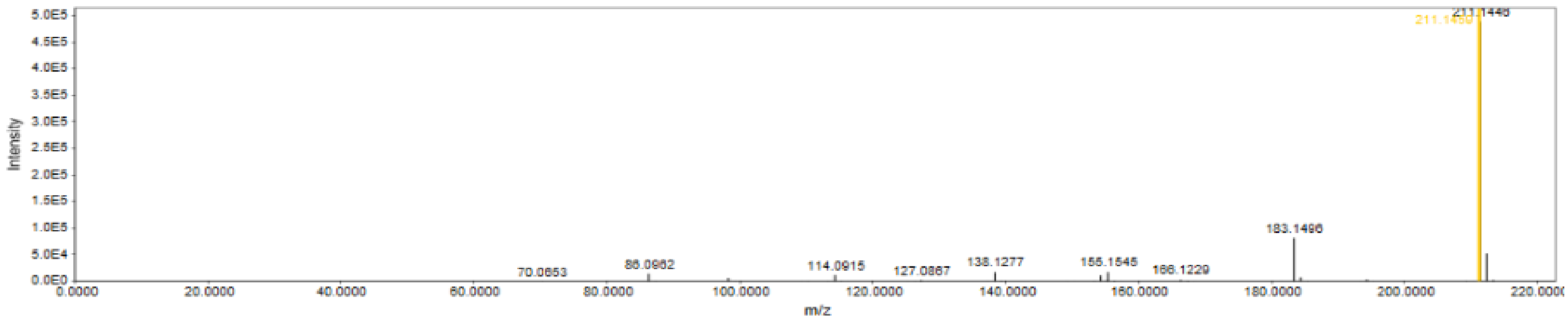

Figure S17. Fragmentation spectrum (MS/MS) corresponding to the Cyclo-(D-Trp-L-Pro) molecule [M-H+], m/z 284.14. Detected on 7.36.

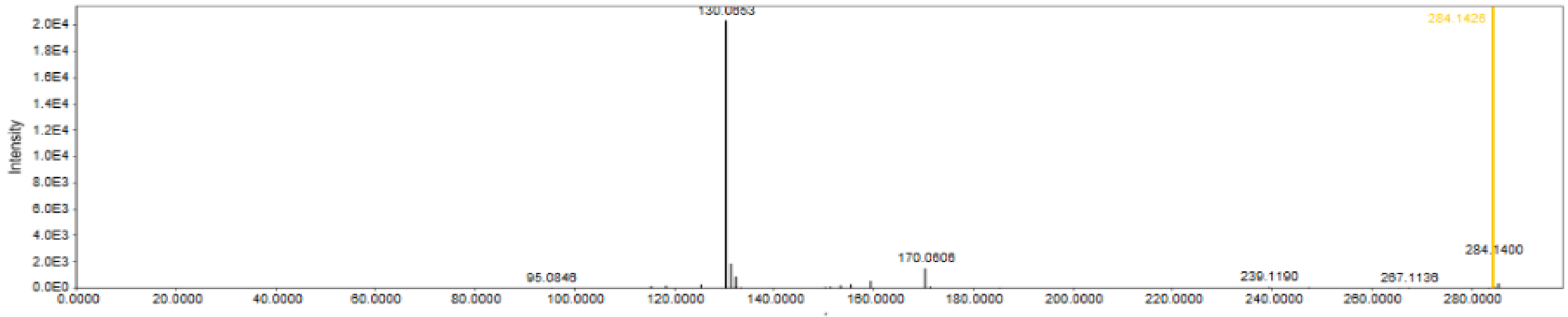

Figure S18. Fragmentation spectrum (MS/MS) corresponding to the Cyclo(L-Phe-D-Pro) molecule [M-H+], m/z 245.12. Detected on 7.45 min.

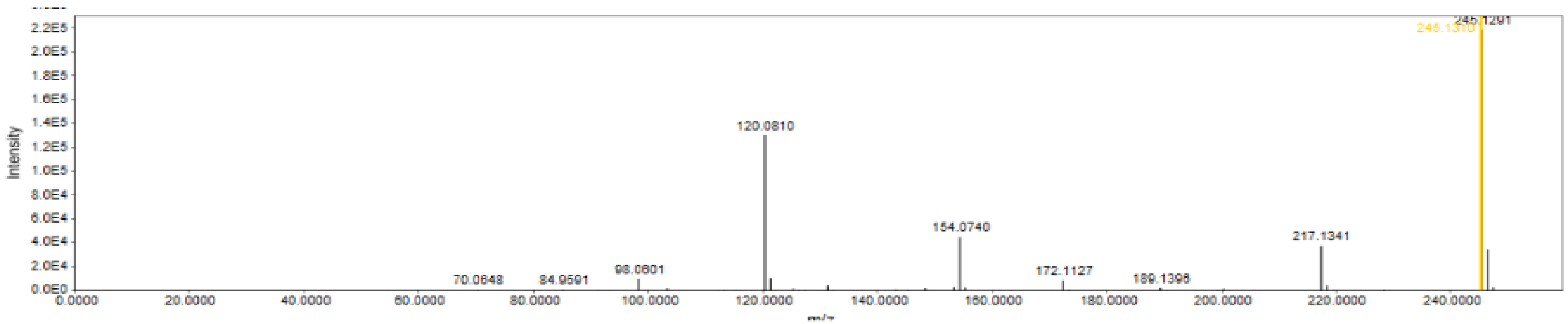

Figure S19. Fragmentation spectrum (MS/MS) corresponding to the Indole-3-acetic acid molecule [M-H+], m/z 176.07. Detected on 7.66 min.

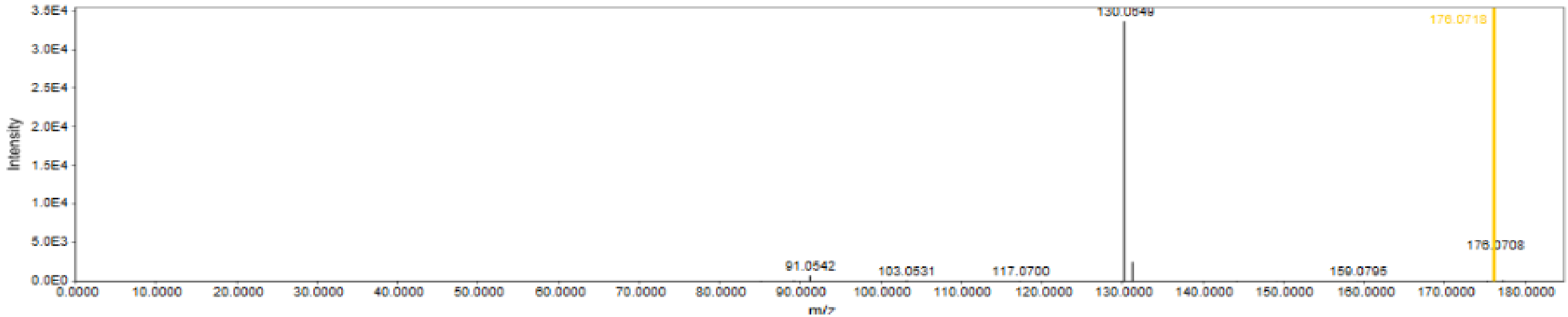

Figure S20. Fragmentation spectrum (MS/MS) corresponding to the Cyclo-(Val-Leu) molecule [M-H+], m/z 213.16. Detected on 8.09 min.

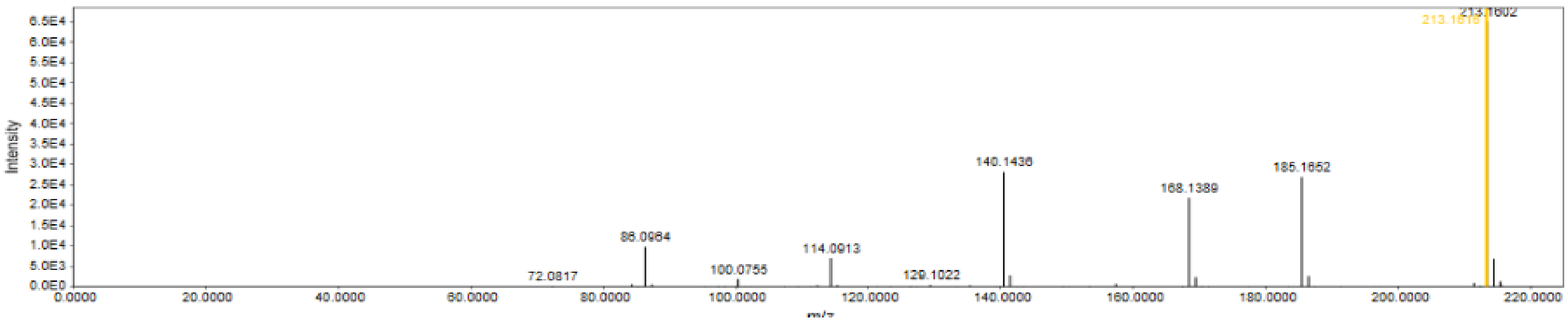

Figure S21. Fragmentation spectrum (MS/MS) corresponding to the Loliolide molecule [M-H+], m/z 197.12. Detected on 8.27 min.

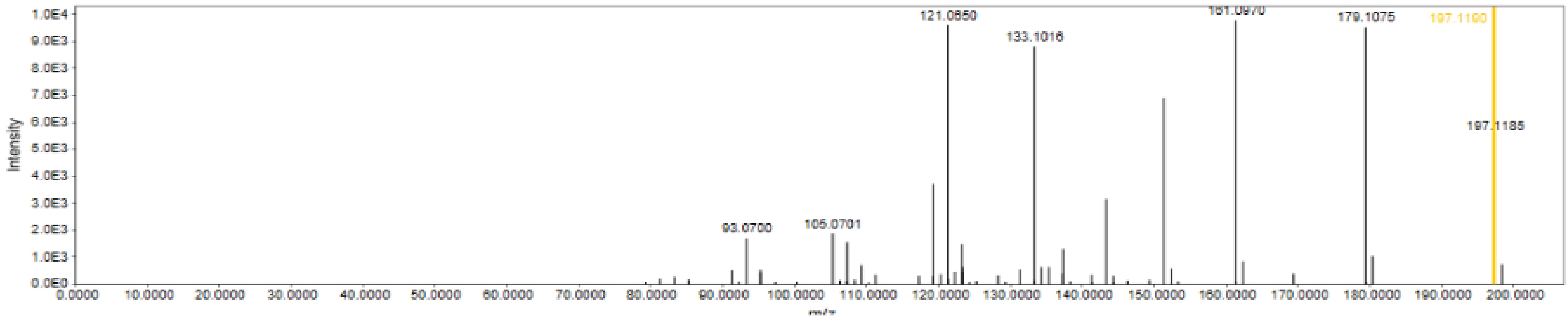

Figure S22. Fragmentation spectrum (MS/MS) corresponding to the Cyclo-(Val-Phe) molecule [M-H+], m/z 247.14. Detected on 8.30 min.

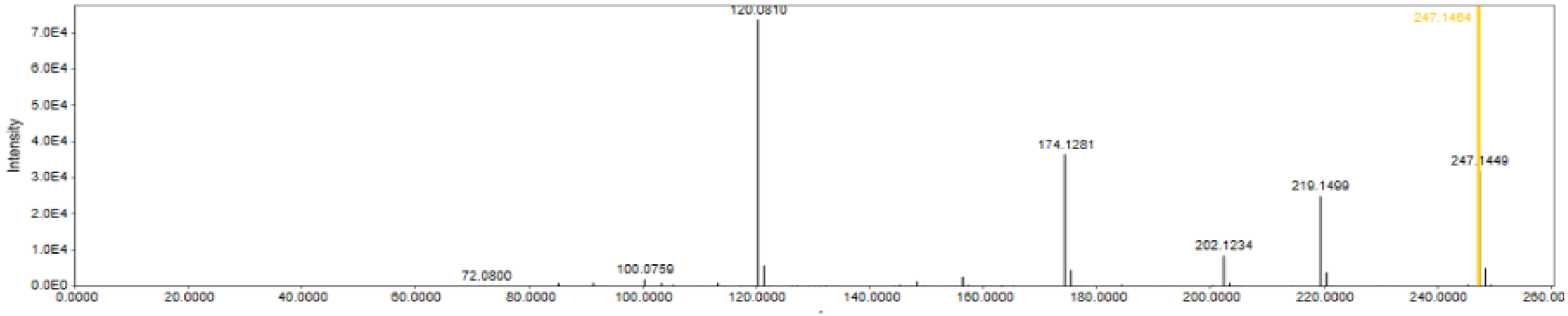

Figure S23. Fragmentation spectrum (MS/MS) corresponding to the (2-(4,4-dimethyl-4,5-dihydrooxazol-2-yl)-5-fluorophenyl)(phenyl)methanol molecule [M-H+], m/z 300.13. Detected on 8.51 min.

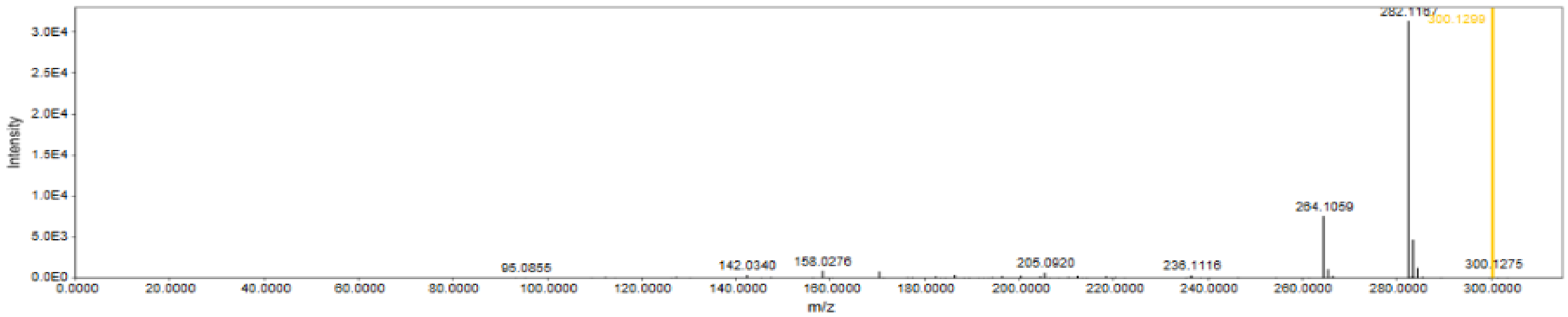

Figure S24. Fragmentation spectrum (MS/MS) corresponding to the Cyclo-(Leu-Leu) molecule [M-H+], m/z 227.18. Detected on 8.78 min.

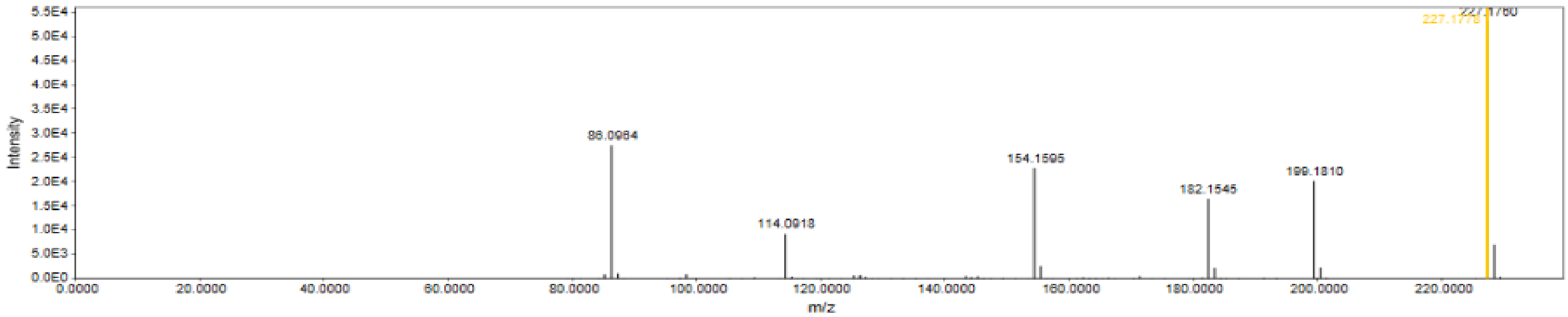

Figure S25. Fragmentation spectrum (MS/MS) corresponding to the Cyclo-(Leu-Phe) molecule [M-H+], m/z 261.16. Detected on 8.84 min.

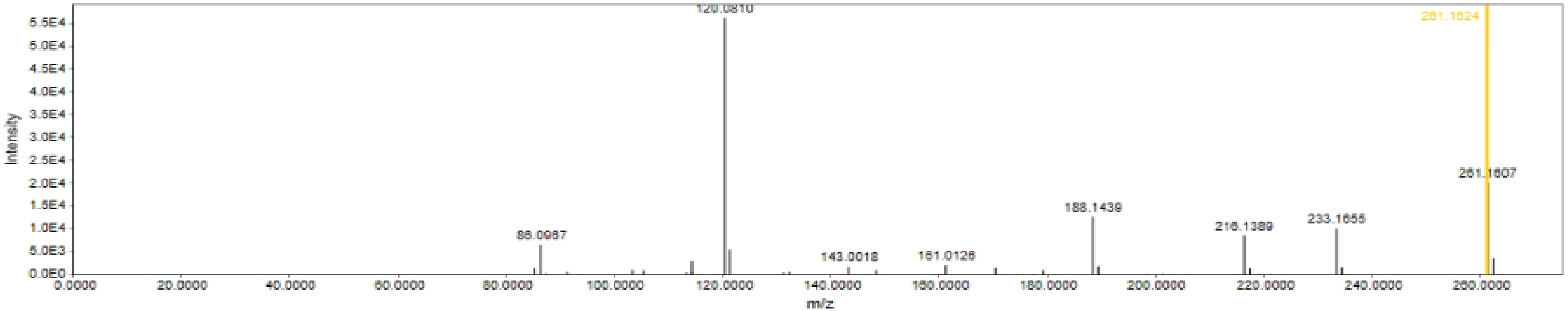

Figure S26. Fragmentation spectrum (MS/MS) corresponding to the N-(3-Hydroxytetradecanoyl)-DL-homoserine lactone molecule [M-H+], m/z 328.25. Detected on 9.94 min.

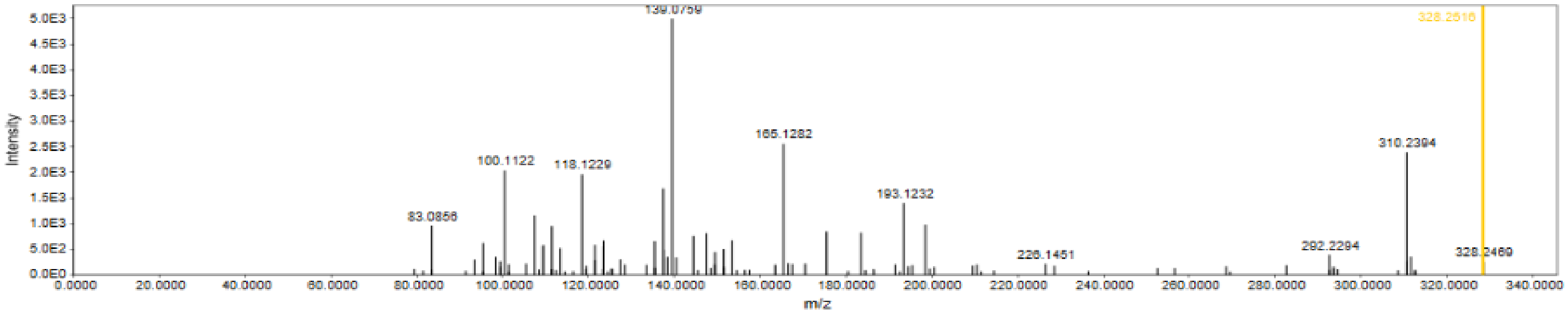

Figure S27. Fragmentation spectrum (MS/MS) corresponding to the (3S)-5-[(1S,8aR)-2,5,5,8a-tetramethyl-4-oxo-4a,6,7,8-tetrahydro-1H-naphthalen-1-yl]-3-methylpentanoic acid molecule [M-H+], m/z 321.24. Detected on 10.77 min.

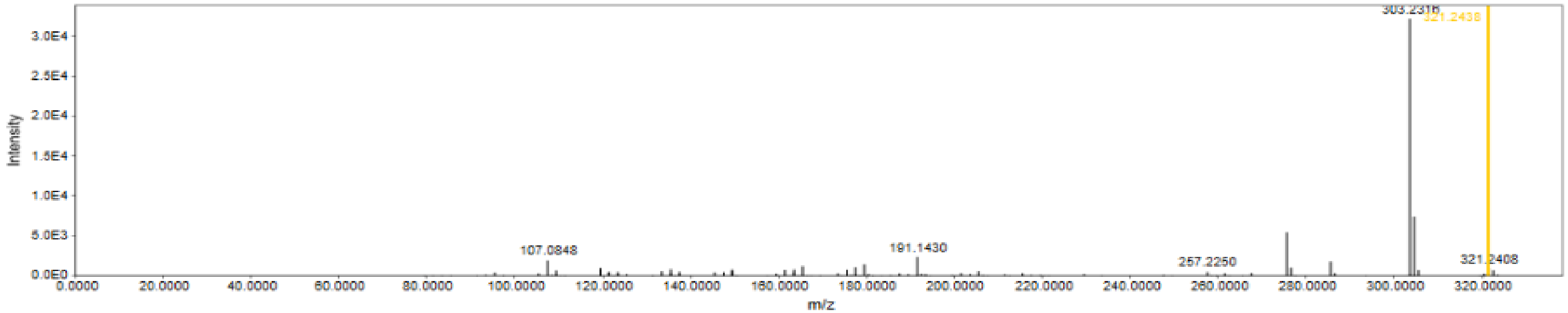

Figure S28. Fragmentation spectrum (MS/MS) corresponding to the Fa 18:3+1O molecule [M-H+], m/z 295.23. Detected on 10.84 min.

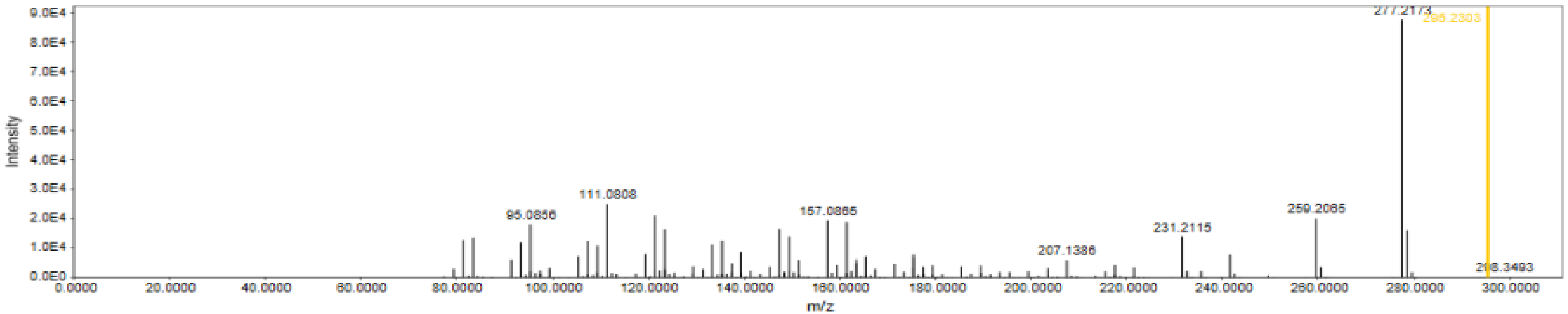

Figure S29. Fragmentation spectrum (MS/MS) corresponding to the (10E,15E)-9,12,13-trihydroxy-10,15-octadecadienoic acid molecule [M-H+], m/z 293.21. Detected on 11.03 min.

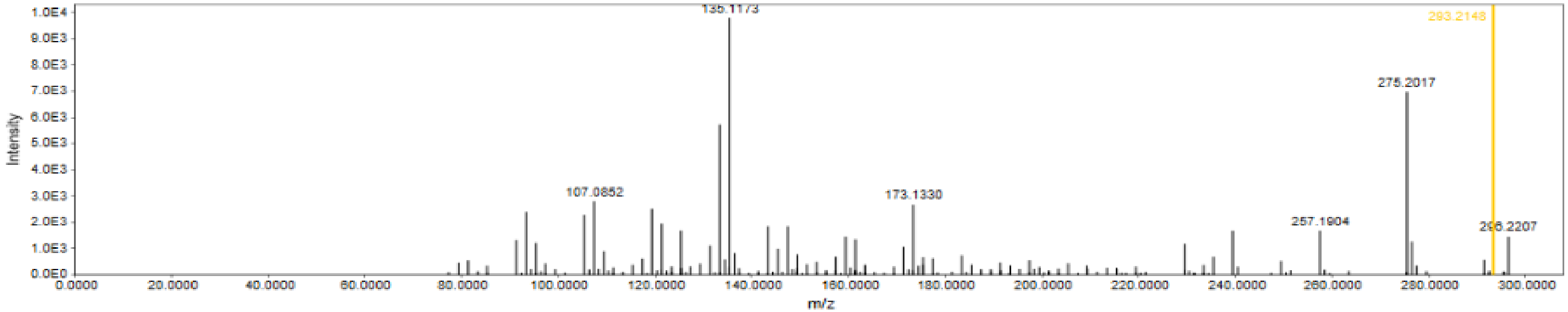

Figure S30. Fragmentation spectrum (MS/MS) corresponding to the Linoleic acid molecule [M-H+], m/z 281.24. Detected on 9.81 min.

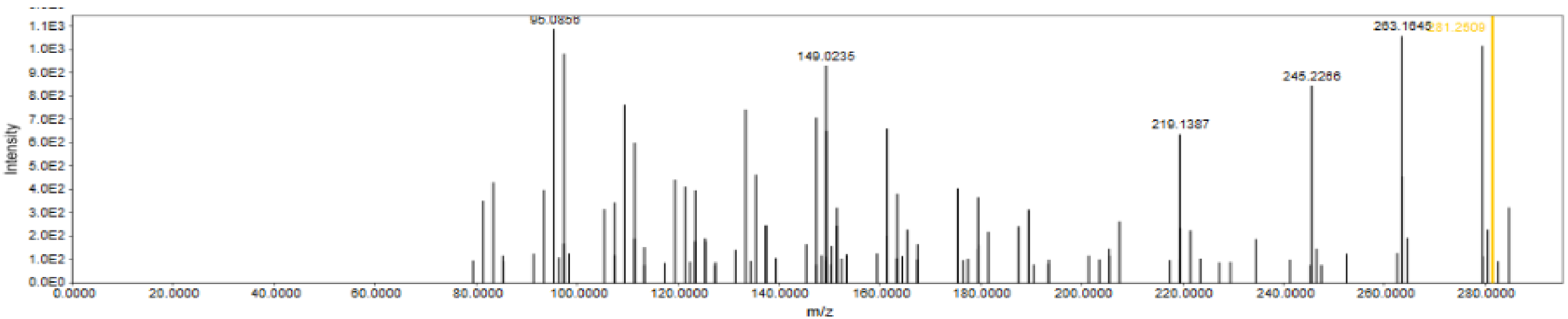

Figure S31. Fragmentation spectrum (MS/MS) corresponding to the Gamma-linolenic acid molecule [M-H+], m/z 279.23. Detected on 12.74 min.

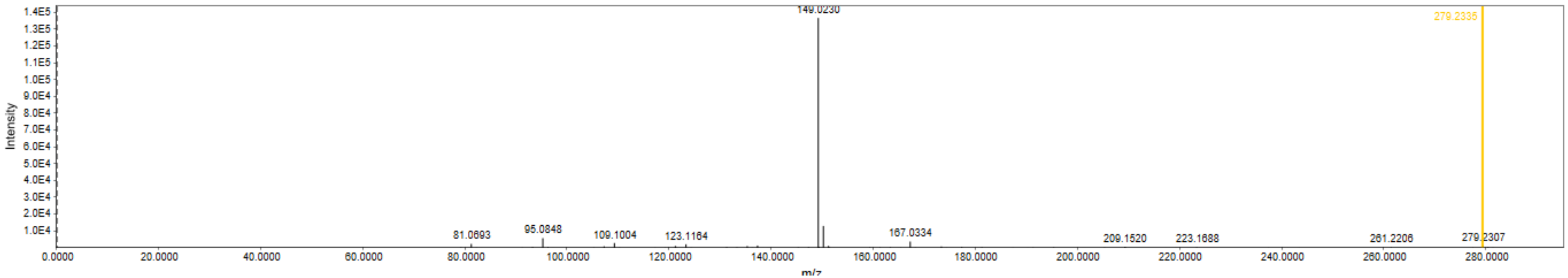

Figure S32. Fragmentation spectrum (MS/MS) corresponding to the D-sphingosine molecule [M-H+], m/z 300.29. Detected on 14.48 min.

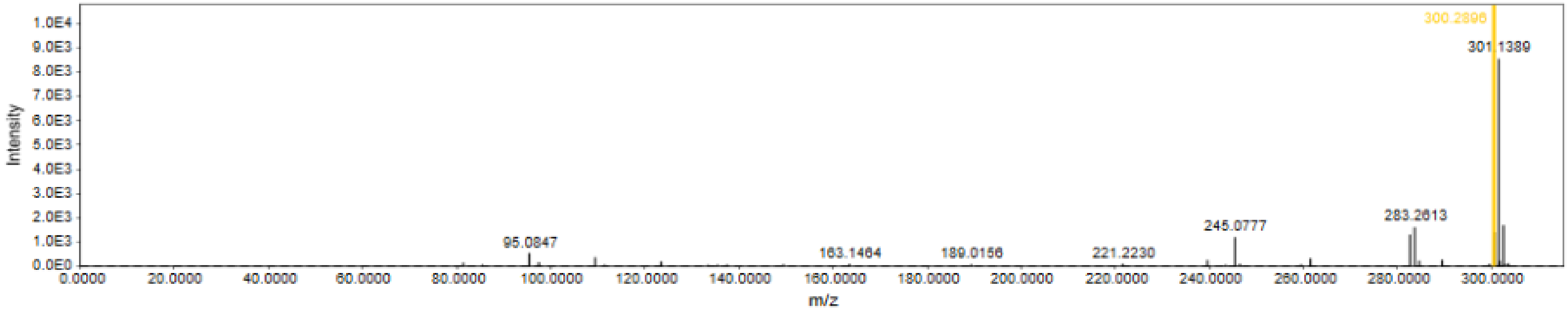

Figure S33. Fragmentation spectrum (MS/MS) corresponding to the Ceratodictyol molecule [M-H+], m/z 331.28. Detected on 14.81 min.

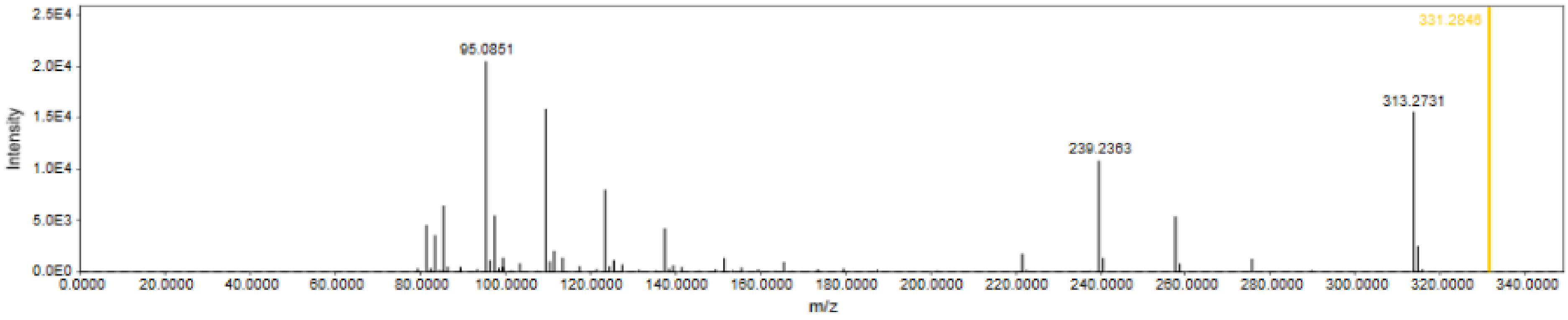

2.5E4
2.0E4
1.5E4
1.0E4
5.0E3
0.0E0
Intensity
95.0851
239.2363
313.2731
331.2846
0.0000
20.0000
40.0000
60.0000
80.0000
100.0000
120.0000
140.0000
160.0000
180.0000
200.0000
220.0000
240.0000
260.0000
280.0000
300.0000
320.0000
340.0000
m/z

Supplementary Material

Table S1 – Intensity of all detected features.

row ID	Nicotinamide I	Nicotinamide II	Nicotinamide III	Sodium butyrate I	Sodium butyrate I	Sodium butyrate I	PD broth I	PD broth II	PD broth III
1	43223813	52739276	6506148	47177655	42552634	5955172	47403415	50631168	45289206
2	40874692	43043814	43959058	9926711	10757445	11344115	16226929	17277017	17246814
3	3178024	29684338	3041348	7147122	6920752	68960406	48922717	48794583	5174776
4	1954468	19212142	19382987	4568886	46202925	45029277	57691343	0	58611465
5	26908104	2965383	27060644	56561935	57233112	5973477	97924756	12090952	11068628
6	19555664	26206419	24681017	27152168	0	26694548	77884015	9647257	93806165
7	19099873	16811464	16741295	36562683	34348713	36705767	10067658	1412523	0
8	27939276	2716437	29257224	5314198	5468569	543562	25101418	24062312	24937893
9	6351777	8051076	8856488	15016064	18558049	16071643	4338764	40393884	4145648
10	7718538	7440915	71297325	21215302	20220738	21300723	6241033	62511005	613578
11	7093123	85392044	8130396	23454593	21508179	23668149	87707605	80098895	7252985
12	2703624	27359622	29937555	68569904	7090987	6683705	10578363	100179297	973329
13	291657	37128304	39324883	6949735	72365105	8267683	17000919	15581989	15269586
14	8100804	9200924	92506525	21297063	22105222	2134862	38967634	35326191	35178533
15	48336963	52839954	52928424	11636992	11715398	12457048	21589941	18113547	18829877
16	12481455	88314716	10277249	19949218	2098954	21859326	32032236	32963647	2993359
17	1424116	16745871	17285864	3502935	36222214	3325378	101558264	9321431	8870827
18	49545082	53114525	4878238	10229351	10525279	99730835	86780164	86899146	82664624
19	3257345	35200192	3507206	80809735	8039177	80178217	0	0	0
20	40920453	43226862	47784766	0	9385803	90441125	0	0	0
21	69323456	75782007	76050476	12126647	11793193	0	11654077	11226964	12050682
22	3545707	36438516	39091191	71674097	6925298	7015083	2980637	3590798	32857174
23	19125499	20220738	20437346	4351669	42982797	4488955	26855935	2765006	27192134
24	5901173	5988121	61711694	11257167	10806564	10848054	0	0	0
25	70845807	73188824	74698975	21960002	21544482	20001385	26496246	22582062	3230498
26	24394241	26606076	27777585	5611957	5972257	560616	9325092	9006283	9032825
27	6974142	8246327	5817886	11995767	12325254	13271004	64677075	65473334	6521707
28	73435327	74551313	77144194	15417551	15396744	14818281	22117395	22359475	21830527
29	18005853	17124171	18558049	40020465	3988318	38250998	7035768	71190546	7151088
30	43498386	46238007	4541734	5752904	57083624	5610736	8331445	77908417	80059235
31	22863347	23835637	24813115	54169185	55133545	54298237	17054003	16413334	19982777
32	7341458	8556289	87552014	13490051	13401884	13235919	0	0	0
33	0	37387622	31676514	62086945	59078845	54566705	11910039	13518424	13575779
34	12810332	13981842	15311993	10758666	11148253	11170524	93354645	8511747	0
35	15390734	15904703	16722807	34254414	34742145	34560043	55407965	0	55176168
36	10097556	1172577	12327695	18300867	17827992	18012566	28244053	26767158	26714377
37	3931268	4018826	42659412	9757086	9618885	8941606	9640545	1144052	10952392
38	0	0	0	80086694	7851553	7836909	0	0	0
39	20637173	21305605	2269372	5382719	53300015	5195308	15437076	20623446	23491201
40	6746856	72023413	71873926	16848683	17493928	16661669	0	0	0
41	10199453	11653467	11869769	22839246	22703179	0	11776109	0	10616803
42	16306555	17587894	18567203	3019077	3174973	33738858	16920377	17217831	16992072
43	0	0	0	0	0	0	19632849	19825659	19422343
44	3503545	3447105	34318512	76181665	7768571	7973585	18231613	19024823	1886008
45	60692725	6005511	6263609	13910148	14327192	13588287	3378157	35435104	3450766

46	22606468	20287857	16657398	33650383	0	33772415	13699032	14790305	14068179
47	10952392	123557625	9915118	0	0	0	86978467	76633185	78305023
48	23399678	25699985	2663658	11739194	12082104	11393843	0	0	0
49	44053632	44560065	4594513	9232958	9495937	943126	6223399	6276178	62915234
50	7912874	7966873	8192328	17997007	17924397	16880106	5056405	4624411	5143658
51	9719866	10177487	10778496	22136644	21099371	21581398	11818516	12403049	11721805
52	16062491	17319423	17786195	42009592	44590573	42217047	0	16934412	16199167
53	1537606	16870955	1726756	32369045	32552094	29196209	22713857	21830955	2163021
54	63151672	0	6302964	4344347	8389715	39568947	18716387	18509237	18390255
55	14094722	13759132	12325254	22850533	26114895	22240372	88278107	8818048	8457138
56	1549199	15373009	16758075	33345303	36161197	34532068	5653722	54945005	5554449
57	3215549	33192764	3685373	7501931	7660573	7218206	18117513	18087921	17630605
58	30712457	31102963	30535513	57507684	60045953	6654722	7305154	7097699	7329865
59	10265961	97351204	10156132	86703896	10256808	1015003	9706443	10913646	10435281
60	6037544	60680523	63914375	0	114374695	115534004	82063617	783935	8155413
61	19641086	19351259	19037025	4159468	46460718	42055353	16654347	16697058	15598463
62	0	0	0	0	0	0	38455098	38806245	3799168
63	6199847	6295032	6411573	16281234	16252556	15307112	10525279	1113544	9915118
64	23488152	25428464	27579282	5769073	6068967	5469484	41276177	42339385	39899045
65	54700943	54487385	61534748	9311058	8966317	0	62666595	65580115	61144244
66	2007125	21810208	21511229	57556494	6396929	6130594	0	0	0
67	0	0	0	0	0	0	10888325	11424352	11038424
68	16123508	16736719	1806992	37451688	4085944	40636728	6990921	72813574	71071564
69	16077745	13759132	155286	35938488	37738464	35846966	20473346	19769525	19414716
70	0	0	0	0	0	0	0	0	0
71	2537538	26903833	2777606	4308683	4326988	42645684	5002528	4991575	5154366
72	35215448	36490683	3882455	5800496	60955096	58050726	66043835	65592316	6412488
73	37271692	3854998	39785553	12284983	12582133	1225173	18986078	20487075	19822914
74	47827478	50432864	5538432	10468229	1072114	10712904	13279241	13462594	13712455
75	85544586	1121171	9558173	22643079	25535242	23384424	11163507	11428318	11007001
76	0	0	0	0	0	0	11455775	11918887	11301099
77	0	0	0	0	0	0	10584464	10233927	10714734
78	3836693	4085944	3666458	13139514	14934303	13851267	0	0	0
79	24739592	26945325	27715347	39682742	40377104	4101442	39879824	40029314	0
80	5343791	5424027	5586635	98541016	10643345	9953863	98086444	9835797	9750069
81	21699465	21605744	22374791	46671438	0	48223594	60357867	60659777	0
82	22932904	22932904	22899345	532335	581453	5617753	13353375	1306904	13459543
83	2996196	2995891	31127368	6547029	6479606	69771924	15067928	14642036	14362582
84	0	0	0	0	0	0	1603839	16160726	16730618
85	0	0	0	0	0	0	8951979	9154247	878754
86	11013408	75049815	0	0	8481239	10494771	8482765	8660627	8691745
87	11654077	11806617	14247262	17786196	21264114	0	18894858	18925977	18938485
88	20541074	232929	22563757	5839242	6272761	6261168	0	0	0
89	3514528	35968998	41948574	75873535	7492778	8020567	10135691	10586906	11147032
90	16895361	1916516	19406174	3098703	32402606	30706357	10565854	98672205	100847424
91	10012743	1075714	10330027	20239044	2075768	19991928	7128512	71770197	7112953

92	30544666	310572	31880917	85504926	8747269	82856824	0	0	0
93	6207474	7010751	63426245	23269714	2437319	2437075	0	0	0
94	31520923	35865268	3431546	64908936	69369214	6760585	0	0	0
95	57379243	5866882	59784194	13644972	0	13828081	15926455	16245661	16163839
96	10457551	11435029	1168733	20661885	21357468	21798005	11566213	11613196	11262048
97	0	0	0	0	0	0	6902783	70996514	70566045
98	8420223	8572763	0	0	0	0	4322198	4379187	4460064
99	0	0	0	0	0	0	0	64920225	6800581
100	90532654	91216034	9351939	10640905	1108144	10845918	8647813	88988947	9029469
101	7777113	8109651	83338855	2311992	23417373	22686094	0	0	0
102	33415472	38168628	36804916	9514242	9311669	9340346	0	0	0
103	6953091	79555853	7283798	16092999	1621259	1607744	1270569	1225173	12695621
104	33444456	35570562	34553118	0	7929379	7654593	40423172	40423172	4881289
105	0	0	0	0	0	0	7458914	71111224	7089767
106	3886116	41564175	40468936	102089105	10824258	98913214	0	0	0
107	14912337	1602283	17029596	41817392	5014304	45530222	62663544	59414435	6428657
108	20755453	22449078	0	45668363	0	47815062	57784543	56969188	59475848
109	31569736	3374801	37817786	47857986	5667481	499966	61830676	59869006	6069883
110	50306562	56907285	5559422	7610509	80000054	78458784	8738147	8333488	8635885
111	104673134	10751038	107693436	24418648	24363733	24449155	7657522	7817079	7666674
112	10932866	11000594	10367552	22607993	24590713	2303175	76575217	8908352	0
113	0	0	0	0	0	0	87567267	8959911	9202145
114	14680476	16035034	17554335	57355145	67435004	6105272	13972689	15101487	14704883
115	14372345	14597799	14579495	34760876	36778376	3617096	12264238	8664288	8145651
116	25450125	26546584	26202454	45304155	48003813	48016323	0	7749046	9579529
117	0	0	0	9213432	12020174	9823594	8244192	88921826	81069055
118	40362156	41918068	3343683	8850387	8752761	104886696	29837793	28393237	28116833
119	56867016	60680523	41368923	11464927	12343559	1132459	39993618	38410251	39223596
120	56040247	5716599	62706256	14322312	15596633	15005997	0	0	0
121	27335217	27945377	27213184	337114	4234518	329487	12718198	12960127	12922297
122	54941956	60729333	6090628	75858276	7493693	7992805	0	9426989	9030384
123	0	100685736	9729324	1800982	19148075	18389645	27670805	2416238	23155613
124	43229913	46527835	4750104	15448059	16084457	15550565	0	0	0
125	49999652	5495721	5475586	11375844	12171494	11794414	0	0	0
126	11021646	10809614	10863918	21030728	2183004	21281199	8359207	0	8511747
127	7854299	7976331	80193475	7240782	78604004	6996107	9546581	10299824	93202106
128	18604117	19969962	20129824	26108794	2630191	2624852	24793591	2485827	2487993
129	13756692	14071536	15070674	16975292	17302034	1710129	17806027	17815483	1854066
130	9349498	9638105	10261995	0	11596112	11357539	12749011	12171189	12511659
131	36951355	3548697	35215448	11435029	11955802	11144897	0	0	0
132	53419604	5569551	56104315	15521583	16397164	14785424	0	0	0
133	25790288	27837075	26713767	5514697	5770019	57629106	18091276	2117259	2040989
134	11757804	12721249	12032072	18566592	19510206	19194143	1458285	13148972	0
135	28955195	30055925	29609897	7881207	8193976	78746475	3624357	40209618	43321438
136	15094165	16031982	15796765	29776167	3012701	28973804	44206173	3764694	43504486
137	81700574	86032715	82063617	26128623	26701565	25938254	17529929	18216359	18692285

138	5867309	6518961	6763941	12531184	12934805	12265458	90517395	9713765	8728355
139	6063781	68905493	64551996	14320176	14696035	14818678	8389715	7962602	0
140	34946367	35285005	33494182	65951704	6816262	69612974	21843767	25230162	15498092
141	73237634	782257	7649895	100850476	10561278	10729072	1208119	11074424	0
142	2388262	2467156	0	62729443	64523926	64554434	123557625	0	12416779
143	0	8877844	11104932	0	0	0	626971	58123944	6111373
144	9689968	10163148	9971863	16442316	16492045	16209845	0	9152416	93354645
145	4030114	39141834	44206173	8197514	7886332	7553794	7971297	8075818	8100926
146	29952808	31649057	29000958	77109106	8015686	7998907	0	0	0
147	2912482	2899882	28321238	27798635	28588794	28871907	31714648	32357148	3272416
148	20467549	21499636	2080741	28006394	29231294	28133918	28510999	0	30687747
149	2701122	27646704	26962715	32123457	3186536	31486448	31585903	31483093	33750146
150	8576333	8998503	9198209	9353831	9676453	101713545	10961514	11481249	11640531
151	3753101	39651318	41024182	9397091	10616193	101936566	0	0	0
152	17276711	16601569	17052477	4301117	45495747	44258647	0	0	0
153	18804554	19528816	19386953	4171946	42473926	4193454	10555787	11654077	17054003
154	14704883	0	19342106	30935167	26633532	26786072	58807935	58064453	5839669
155	0	0	0	18731945	0	14887931	11635162	11115305	11189135
156	72890454	73500615	7314123	81325625	8295902	8319272	11471028	924394	12630335
157	39812097	40438428	40347817	49068853	5122394	51369155	0	0	0
158	53301566	0	53436293	0	0	0	36332043	37567618	36878137
159	3194285	33124426	32380334	7182237	76879385	7711643	0	0	0
160	0	0	0	0	0	0	65436725	66184174	6083916
161	43642993	0	44847754	41940034	4220149	0	12505251	103605354	11147643
162	1147957	11453639	11914005	15247316	15947476	15250366	12095834	11810278	12359119
163	9422992	9655342	9307214	79775815	0	85146455	13556041	132266455	0
164	33172935	32228708	31615193	23453066	24955588	24227666	37231726	36587395	3773877
165	20510261	21932546	21385232	15634768	16995732	16037474	25030334	25008367	2652889
166	23284663	2354215	23853943	5524002	56631797	0	115930605	0	9182924
167	0	25096536	2551053	62599175	6311537	0	13362528	15559108	18853977
168	11794719	11937496	12014377	29331665	30679814	29858232	8237175	9915118	7291425
169	0	0	0	0	0	0	91875006	9294279	94520056
170	0	0	0	83195465	8865641	0	11016154	11037814	11157406
171	0	0	0	0	0	0	696743	67346533	696987
172	47482736	45460052	45844455	12753282	12734977	12958601	0	0	0
173	0	0	0	0	0	0	19750304	19253633	19436987
174	0	3178848	31345195	28211104	28075037	28369136	24037297	0	22716298
175	6034005	61313564	60690894	81236543	8437704	8385139	7590404	76331154	8118193
176	0	0	0	84462555	0	87481086	57223956	58194116	60113074
177	12137904	12255055	12202276	0	25230832	25303717	0	0	0
178	35170295	34533594	34775522	3441339	3450156	35325276	7138885	7901586	0
179	3152855	3178848	0	0	28327034	28039648	24037297	23680353	22716298
180	0	0	0	0	0	0	9018181	8786625	87054736
181	3855303	38760483	39099124	0	0	0	1568114	16161642	15909646
182	10915782	10672938	10978019	0	0	0	27661348	2689102	0
183	31599023	32959988	32165862	2143801	23411577	22300474	68829224	6595658	67442935

184	12146782	12196815	11910344	2452634	25706392	25726528	8084635	14369294	0
185	1346534	14064214	13388154	18184631	19805219	2007247	14948947	15315043	0
186	79755376	81681655	7952901	15506299	16833246	17157395	46494274	46921387	6083306
187	11692212	12211154	11494215	2312602	2555019	2633608	0	0	0
188	10821512	10612532	11037814	1764952	17811212	16909089	0	0	0
189	8619441	8623712	8518764	10290367	10781547	10580193	64269487	6396258	6392017
190	68841425	7201121	7124241	66080444	63609295	55585674	74698667	81736875	0
191	25333888	27139966	24711525	24742033	0	20318365	2508952	2638001	25455005
192	50119546	5074435	5134719	8947951	9217032	8910091	31697868	31880917	37250336
193	48737534	0	48822954	5439159	54704604	53519062	0	0	0
194	0	0	0	0	62846594	8816828	5541941	54062104	5698142
195	39223596	3847981	39118342	37111523	35947642	36764036	10888325	101408775	113856064
196	13037617	12618131	12856399	13184362	13619711	12665724	0	0	0
197	17640062	17695282	17435963	15837952	16182998	16133574	0	0	0
198	9336685	943065	9628952	15460872	16791328	1643591	0	0	0
199	77029785	7627929	7415898	46930542	5123218	5203149	17015868	17067122	16755023
200	19342106	18945501	22057323	3545036	3953844	43016357	0	36721936	36904678
201	0	0	0	0	0	0	882415	87063885	89718085
202	20324161	20851646	20765613	46847256	5168461	4970769	13759132	9732069	1726756
203	11169304	11500621	12150443	16364825	17210509	16961259	19494648	13240496	1348456
204	44677217	44427354	4599913	8689609	9108576	90101875	29104685	27304708	3273514
205	0	0	0	0	0	0	22625994	0	2149872
206	0	45833774	47332637	9572757	9744761	9645152	3639611	3404699	36030014
207	0	0	0	0	0	0	10667446	1089107	11445096
208	10143928	10751954	10629312	13241716	13847911	13458933	0	20348873	16992987
209	13290529	13741743	13382969	0	2776965	27565857	0	0	0
210	0	0	0	0	0	0	17922567	17666909	18056498
211	0	0	0	0	0	0	16581433	16908479	17196476
212	0	0	0	0	0	0	29216648	3088117	30505005
213	12904907	14826915	12264238	15009963	16992987	15925204	11319708	11558892	1172272
214	87784795	0	9229205	11116128	116272295	11463585	12542044	12523435	0
215	68332856	68355737	6871543	78514614	8098851	8270245	9962832	10225202	9858617
216	29213293	29986672	29592507	5041517	53828413	0	2288104	24528477	25748798
217	6213484	64831445	63667563	11041658	0	11231266	42406197	45518017	44816334
218	10838596	11033848	10985035	11581467	11674823	13015347	15490465	16625365	16162863
219	34007327	33467337	3382123	3113042	32030408	308711	808555	85245605	78982306
220	0	0	0	0	0	0	56836505	5920698	5751379
221	9836133	9927291	10079557	0	0	0	0	0	0
222	0	0	0	12203222	10525279	13606592	1159062	10999069	11336488
223	77242427	76213086	78548784	80518384	0	79511924	0	0	0
224	20434905	2069819	20213722	17219052	1795094	18175784	10830359	9976134	13515068
225	73457294	71214954	7301188	5607075	5968901	5673888	0	0	0
226	0	0	0	0	0	0	15815681	15877612	15430974
227	53451636	54843413	54661284	49377896	49885244	4813866	0	0	0
228	0	0	0	0	0	0	18831401	1872981	18535474
229	0	0	0	0	0	0	30918389	30715205	30391514

230	35056805	38391336	39831317	25825067	27527417	25465073	617361	64521484	6629705
231	20739985	20726257	21420315	28641572	27804126	22690977	0	0	0
232	5253426	5303917	51308447	52521143	54584707	5337903	11766957	108181564	12657792
233	5652929	5861818	58113574	9691585	10040476	10091606	4908746	4121638	41490955
234	18719713	19247928	0	0	37458156	36404957	9716815	10973747	9671053
235	4971837	51923794	49963955	58125166	61102446	59123696	0	0	0
236	40963165	41262143	41747223	40038773	46649866	44047528	70339374	75287775	6885058
237	0	0	0	0	0	0	70159375	6708111	67737036
238	27605212	27315386	2775928	2586595	2757562	26455364	10410508	10331735	10721659
239	42483687	4330405	43043813	81075767	8637837	8376566	25931847	28341984	28647064
240	3014806	2927858	30437888	2764945	27621994	24918979	7026615	662879	7025395
241	5922681	5868407	59537993	124680625	12895785	0	4515192	4405363	4234518
242	12581217	13206937	13330189	38664077	40628796	39433186	0	0	0
243	20165825	20226841	16230286	1848788	22301388	19738712	12211764	12268204	12385355
244	6697494	6895461	6764002	13191927	13278723	13471808	23216629	20470905	1928109
245	19844758	20644436	0	37677875	38092875	0	11684585	10781547	1068087
246	23614456	24067195	2388018	2473288	26866614	25916592	38470657	42253654	47440025
247	0	0	0	0	0	0	31613362	31147808	29623015
248	58180083	60334253	58055303	10844332	11147856	10863613	31087708	29928402	40057076
249	0	0	0	0	0	0	10071014	94944116	9467565
250	0	19311598	17603148	0	0	0	13689574	13265208	13886351
251	0	0	0	0	0	0	26607905	2659204	26908713
252	17945753	18194698	18479644	5718033	58776514	59622197	0	0	0
253	53184418	54696242	54295645	0	0	0	45066498	43952954	43226862
254	0	6396532	6319652	0	0	0	0	0	0
255	0	6516032	65827534	46318853	47512637	4745589	14747594	14463869	15391313
256	80787466	81637417	79954595	82092295	8728019	8319516	11165948	13545576	11013408
257	15205214	1760925	16221133	1859466	19271938	18393306	29566272	29255393	30578223
258	0	0	0	0	0	0	15488634	1585229	16159202
259	0	0	0	0	0	0	20246061	17325525	1697102
260	17025568	18211266	17195498	0	35350387	36048594	10320875	943309	9484954
261	12682198	14332684	1359439	18158394	20190231	19101093	77240295	84150366	7984568
262	6116316	66358374	6240575	0	11412789	11683151	4567056	4518243	4878238
263	4335103	46299634	4503599	9082705	0	9619129	16861559	17943129	17717125
264	4158004	41680103	4030236	43671978	45603745	46233125	10607651	10122572	10372739
265	13775424	13926866	139295205	0	0	0	1181882	12023225	12197121
266	16748922	22514944	23064089	21538687	2132513	20043793	8393986	8678931	871127
267	8775673	8873512	9069648	0	19851104	19117568	0	0	0
268	4370523	4368845	4478674	21151235	0	21990815	0	0	0
269	5191922	50548184	5003687	41017163	42113623	3891638	0	0	0
270	0	0	0	0	0	0	12469436	12003303	12807343
271	0	153455515	13698116	0	0	0	6946379	6830753	68560754
272	0	0	0	0	0	0	2147401	2077202	2172143
273	40835642	40922285	40581204	45381953	4554334	4600462	22054272	21517331	20934627
274	0	0	0	0	0	0	5185759	4757121	54206714
275	0	0	0	0	0	0	2662438	2454861	25366838

276	0	0	0	0	0	0	17221187	16370012	17539386
277	18088225	17695282	1780023	13224937	1356144	13704828	0	0	0
278	18174563	17788331	18467745	14080077	14325363	14232313	0	0	0
279	4465525	44231797	4453871	33676926	31576143	30879949	0	0	0
280	80968376	10430704	9826644	0	0	0	11124152	11305065	11546383
281	0	0	0	0	0	0	2321541	22570774	22767551
282	0	0	0	0	0	0	5639658	58056826	55956045
283	7267354	70973022	73157705	54703994	54592026	5721267	0	0	0
284	22811177	23175444	0	54860806	53504414	53620044	0	0	0
285	50618657	48104795	48690854	37352537	36921152	37910225	0	0	0
286	14640357	15201492	0	25636498	29360922	28625129	9027333	8270734	86063225
287	18966858	20512395	2088734	24592239	2452756	23778284	0	0	0
288	16405707	18818589	17470133	17500336	20615208	19706373	0	31606344	3322327
289	59518164	6051181	6066374	11643338	11884291	116096875	3715881	47592564	4719596
290	15340975	15734224	15703716	3792609	4225091	0	11379504	0	12111698
291	16896886	17451217	16848683	18947333	20892832	2014996	2288104	0	22850533
292	59225287	675967	6971091	19040077	22432573	21679024	19874778	19264922	19081567
293	0	0	0	0	0	0	11605569	12264238	12116274
294	0	0	0	0	0	0	2360744	22756262	22472234
295	26902002	28699536	27278777	55835537	5730267	5834696	21508179	25138638	22545452
296	21621059	22453928	20428194	3029755	27380978	30575174	90276385	8759778	94141754
297	1336863	1568114	14622511	13258801	13679811	1359439	12472913	12504946	12324644
298	41872305	42500772	44938364	6306015	6109848	600368	11406046	11713873	11519231
299	108791725	12020174	121818665	0	33070732	3261311	674167	7263968	6434759
300	36809495	37172234	37369316	38863296	38812651	3876445	0	0	0
301	20368397	21383706	21545398	39934739	40501577	4007538	14796407	16138762	1348456
302	1632608	16395333	17263593	37871174	38144832	36883323	0	0	0
303	27621992	27402334	27975276	33977432	37215557	34835623	16230286	14491326	2071497
304	67386194	69265485	7109597	3556324	4128655	37738464	12540336	1230939	12640098
305	6650115	6656217	65498354	59948633	6070157	5978572	0	0	0
306	3174973	30895508	33085986	100279976	8758862	11239167	12575725	12520505	12368881
307	0	0	0	0	0	0	12282238	12124816	12336237
308	0	35178043	34756453	42488566	42898566	42518742	5384061	54023663	51158957
309	0	0	0	0	0	0	0	0	0
310	0	0	0	0	0	0	6824347	706292	69256335
311	19340826	19057406	0	17951549	0	17795988	0	0	0
312	13051346	1556521	14527936	0	0	0	1702319	16507604	17262678
313	0	0	0	0	0	0	93861084	8821709	810355
314	42260977	38075273	44654336	49861753	59826294	58697495	0	535935	611595
315	0	0	0	0	0	0	7128817	7378678	6976582
316	71178345	0	7106211	71155156	6966301	67648257	0	0	0
317	1770718	18374696	18284392	40270327	42009897	40103752	0	0	0
318	78539935	8222226	80010425	19233804	21008762	20127689	0	0	0
319	9799492	1064426	10241554	16983834	17339558	17355422	19677696	1943363	16291302
320	6928379	82585304	7181596	12197121	12889653	13783539	691343	73911865	7292645
321	16382826	24558985	1693197	57031757	6166288	606012	3697576	38836754	41002827

322	53080963	5892936	5658939	11857871	11782211	11794109	9518513	9457497	9488005
323	25721646	28603743	27258032	5711596	59853145	60014224	11257472	13911673	16840446
324	7382949	79043317	79870087	11171439	11525028	11422216	0	0	0
325	45932928	45990894	4635699	14250313	14299126	14634714	24527256	2501874	24328342
326	31917526	32262268	33278186	64341484	8569713	6687366	14963591	15553312	14816237
327	12824061	13529407	13637101	20614293	23317307	21258012	64856465	66801655	6390614
328	7237426	7174274	7583997	58862244	5968901	5824598	65876044	6467097	7144376
329	6113509	65952313	72624426	46003094	4593903	45118362	0	6082696	60579846
330	12050681	15977068	14967252	0	0	0	68505835	72413916	7120885
331	3893133	39965552	36902542	57629715	56012787	54609417	16387401	1606066	16769058
332	13410426	13848827	14480953	31779326	35360361	32968225	82615814	8697846	8362258
333	55326355	57852423	54905347	1450536	16397164	15053894	0	0	0
334	2022379	23228833	21254962	32247013	3557239	29287733	10855681	11107373	10832495
335	11185168	12007665	12170579	19289327	20222875	1914655	0	0	0
336	11461571	0	12299933	22680603	22845957	22891414	0	0	0
337	74065015	75848213	7904393	20500559	21097723	20458885	3700627	31026691	3413851
338	29856099	30380227	3154594	40648933	4144855	41551973	25504734	22789516	23460693
339	90586045	9223104	9530838	19834934	20874008	0	53541637	5393824	51223022
340	3117008	32060916	31523972	30422632	30852795	30636188	76596576	74091864	7537625
341	7547693	81789044	77121313	7590709	8537984	8000127	1842595	17908533	1810226
342	1693197	0	15162503	16565874	18945501	21416655	6688586	6453674	67505176
343	4991118	52635547	5259894	13657236	14401022	1352056	0	7718538	69863443
344	16865463	17409116	17752637	44388306	47219453	46048857	1647435	14491326	15070979
345	12090647	11783126	12097358	21266555	22244033	21471265	15009963	1223373	15254027
346	0	0	0	0	0	0	984678	98672205	10127759
347	70699365	7729826	7864977	48065442	50277274	50576254	8189887	780213	8454392
348	94855646	1035901	98065094	7474168	8094397	8359817	9571597	99407446	102345374
349	56863965	600429	5776395	48388824	5446603	0	13985198	13841504	14348243
350	28173884	29824065	29760608	65517266	66790674	67181787	0	22057323	24681017
351	5730938	70235645	66300104	17413387	17613215	19657255	0	0	0
352	26036184	2785843	27607043	4349533	45159243	4464823	20623446	17633656	24436953
353	0	9854102	12721859	1208119	12172714	14521834	66641797	71303424	6415539
354	0	0	0	0	0	0	98464746	9804374	97354254
355	54142645	6180627	6010697	87899805	93037366	8477273	0	0	0
356	47162402	5348672	5233047	11596721	1312487	1227339	0	0	0
357	28952145	32176846	2893689	9238144	9973388	9250042	0	0	0
358	4060927	44254984	4162519	101607074	10639684	10451449	0	0	0
359	55808386	5617143	5959138	1128798	11845668	10593312	68380756	7587658	75873535
360	0	0	0	0	0	0	939282	857917	87179816
361	20928526	18853977	0	11867633	10494771	1223373	6281304	60229004	55573474
362	0	31415059	31956882	65055376	6915413	6621285	23125105	21904783	1848788
363	19244786	20398906	2064236	59389727	6215711	60074937	0	0	0
364	23123274	24464104	25804934	4176827	4336354	42342437	0	0	0
365	11013408	9213432	10616803	8725304	8420223	0	14431531	14541055	14926066
366	0	24711525	17969244	39324883	35358837	0	15930391	15815071	14647222
367	0	0	0	0	0	0	28248628	28848416	27784905

368	9854102	11867633	10555787	14003197	17694672	123862705	20095656	20501108	2119547
369	3795812	4121333	38885565	14173127	1429943	14503834	0	0	0
370	25312534	29214514	29360953	96036304	10033794	10299824	0	0	0
371	8129481	89666223	101790125	47427823	52336566	48468146	66254346	60936786	6662654
372	0	0	0	9304956	0	108608675	74518976	7586133	7641963
373	71459015	7062615	7249629	19672814	20431854	2047487	11196456	8572763	7138885
374	65467236	77557574	6779195	13554424	14473937	13211208	0	0	0
375	11699229	13488832	1276335	27080476	26864478	2670248	10342231	8725304	0
376	48928818	54115186	5287046	18432356	19619425	1861632	0	0	0
377	10372739	13942181	14521834	8231073	924089	7983958	10643955	10366332	9370244
378	46799356	49099664	5343486	17917686	19854948	1884696	4859842	49729653	49913926
379	48355267	54209766	5150065	11670247	13078804	12018038	39109192	39302917	39448745
380	0	0	0	2602337	0	29531797	9055828	9269537	9347851
381	0	0	0	0	0	0	10686025	10768123	10645847
382	0	0	0	0	0	0	13968052	14528546	14808854
383	31869019	36109333	37106033	0	0	0	0	0	0
384	5109733	5324571	5451118	7709477	8305452	79867646	0	0	0
385	28786576	30466137	30843521	0	0	0	22405115	22896295	21605804
386	6924291	7313055	7516849	8734608	8792543	8679267	52473854	46799355	50155243
387	441299	41786884	47177655	14501699	14412615	14988912	0	0	0
388	609734	60845264	63682513	1223556	1174316	12877145	0	0	0
389	22621722	23115953	2773182	0	4365703	40545204	11053373	10090539	90535706
390	55235747	5676786	62198296	15842925	17480871	15698927	0	32399555	3606052
391	14453191	15854731	16660754	24428716	25350974	24763694	6531775	61077126	6547028
392	28921637	35911032	3806795	7021429	83711053	77676556	0	0	0
393	0	0	0	0	0	0	6564113	6446657	68457025
394	26328451	2696912	25535242	36945255	44328205	40453682	16447808	16347742	17299897
395	17865517	18661777	19189566	21648515	22280032	22374608	7497355	7076038	7450982
396	0	0	0	0	0	0	11010662	10863308	10826698
397	0	0	0	0	0	0	40763032	4077127	4325249
398	26386417	24272209	26883698	63737427	6627265	68121436	0	0	0
399	0	7718538	8328699	13057447	123862705	12874399	11857566	12122681	11938412
400	19830236	17999752	16504858	29470781	35511375	3624357	12176069	12759994	1266786
401	41149265	40767914	43550247	16492959	17610165	16461232	0	0	0
402	4405058	58371063	5612872	17883212	17691316	17432302	0	0	0
403	2094622	21502688	2155943	7786815	80391777	8006839	58941563	58605972	5970426
404	24772541	0	29318241	0	0	0	26908105	27213794	2738098
405	37903207	39709283	39337085	13423849	13303647	13403104	0	0	0
406	21169539	22280032	23570523	5807208	65583167	60241205	0	0	0
407	43412964	38165577	41338413	0	0	0	9831221	96323083	85025946
408	11522283	11972886	12094308	37754329	38519165	37469382	13393036	11959158	12752367
409	44468237	0	4484501	10517987	11139406	10552096	35938488	3322327	35816456
410	31882444	3388621	3101327	4321039	43724756	0	20196333	22789516	21935291
411	38013037	39254715	40005212	13190768	14595664	13249038	34832876	35707542	34653184
412	18018057	21883427	22395963	69152606	75495233	69927515	16332487	16704075	1692953
413	39782504	48181372	46799356	101970123	10933782	101841986	37981309	3891882	37882156

414	4309568	4578832	44596367	9505608	10702806	9922593	0	27243692	33802925
415	27002373	29133667	28581472	36036113	39384067	37452603	18152292	25077621	21386147
416	5687006	68997015	65607574	10776055	11741025	10778801	0	0	0
417	630571	66974335	6196186	13656626	12948534	14404988	0	15559108	18609913
418	0	0	0	0	0	0	19545596	19701492	20186875
419	0	0	0	0	0	0	13022363	13659371	13497678
420	0	0	0	0	0	0	4100618	4124628	4189183
421	59301556	6005511	596585	31124316	31691766	3286938	24234072	24043093	23922585
422	8572763	9701561	1003715	11196456	9762578	10769343	706292	71837317	7210884
423	82823267	909018	91100104	13288088	13269174	12994296	0	0	0
424	1456546	15448059	16284895	3761582	38586587	37359858	12569319	1928109	18152292
425	9279452	9543225	9654854	25783822	27310201	26542436	0	1708451	17938736
426	72551816	0	75958647	9786496	9661139	9748086	26328451	23796282	21874275
427	2579883	28286458	28525642	45008535	4729511	4550734	37524906	29287733	34443592
428	19689899	21459366	23054938	42284164	3889777	4609767	12446371	11556146	10903579
429	13222191	16584178	19293294	44816334	5412129	50856926	63609296	59786633	55689404
430	17963142	23054938	2155089	76331154	8734456	86063225	87552014	79455176	8245412
431	3461749	37863547	4242755	7218206	7739893	7941247	17150103	1946719	18542795
432	64371994	6537876	7324984	1446997	13435747	13945232	9079502	9456277	9192077
433	65915704	7318577	7560506	81984296	8685338	89248267	14879999	14349768	14368989
434	15687852	17578741	17734332	11508248	11527163	11569569	15012404	14660035	13804895
435	78295874	8246937	7911044	7313086	8145956	7812198	79815173	8023618	81636505
436	89788257	8680762	8764659	8718287	8720117	88689966	9262856	84214435	86288983
437	10710157	1233227	12165697	81660913	8802489	82512085	11910039	98141364	9927931
438	8758862	9988337	9942575	8795472	8829031	8423274	21609465	2577839	26132285
439	6240118	69967175	70726825	58255133	615622	60268665	7011666	66843146	6560147
440	13238971	14251838	13889402	3091961	29817659	0	26694548	29562305	29104685
441	4659587	4846418	46526616	15816992	0	15538302	0	0	0
442	350385	37286945	38153372	17223323	17050037	15793715	0	0	0
443	36346382	0	38338557	75195034	76104785	7337431	17115019	1693197	0
444	6604994	5393824	52290806	7443965	78649765	7419559	19197986	0	19436742
445	0	0	0	16044186	16523163	14210652	30158646	0	30229334
446	22103085	19683797	19448885	4220179	0	43815668	0	0	0
447	210231	214014	23173918	6093069	6244999	6085747	0	0	0
448	8660627	10302266	10915172	6670891	7303933	72090533	13606592	13536424	13957436
449	0	0	0	0	0	0	16129303	15916663	17313627
450	0	0	0	0	0	0	14747899	14978235	15532566
451	23050056	23998552	22772126	2043399	21610686	21154285	18566715	0	18858951
452	120659355	12859145	117883125	18667879	18774657	1927499	25327788	2499647	24566917
453	20287857	2416238	20013285	9620105	101570465	0	4829425	4881289	4435871
454	727312	500027	45853607	2174187	2287128	21691533	8902251	9073096	92744484
455	2219461	2670065	24452206	0	22054272	21145132	33846855	3498145	33907568
456	27768433	30138907	0	0	0	0	22288574	22491147	22506401
457	15751614	17565928	17532979	5433454	54899854	53106895	0	0	0
458	6310896	6963464	666601	18195614	18120564	16887733	0	0	0
459	18514728	0	18132157	7202311	7346004	72060635	0	0	0

460	38336423	40099786	4541124	94324805	100841327	10112505	0	0	0
461	29937555	3192973	340897	7117529	69558365	8221921	14105094	14359531	14412006
462	80733466	8983097	81947687	1942753	1863737	17969244	37051423	37896497	40094602
463	22759314	24634644	23543372	42436703	43998715	40502493	9616535	98702705	10362092
464	40760348	42703742	41822484	0	0	0	0	0	0
465	0	0	0	0	0	0	0	0	0
466	11740842	12047326	11826661	78447803	8055591	79246504	13249099	13497252	13352338
467	0	0	0	0	0	0	0	0	0
468	0	0	0	58613996	60939594	59779465	0	0	0
469	0	0	0	56459215	0	57214715	0	0	0
470	0	0	0	54973687	5608143	55178394	0	0	0
471	21728752	22388428	0	20477037	19969566	20089248	0	0	0
472	0	0	0	37102035	37686387	37706582	0	0	0
473	21728752	22388428	0	0	0	0	20085404	19638096	19218121
474	10965815	11380725	10989307	1089107	10335519	10697344	46021402	5507009	44334305
475	13173378	1302755	13584626	11362725	10767513	11350217	41536716	46341736	41731967
476	0	9644816	9775086	9272008	8393376	90520447	0	36161197	32545993
477	12672435	12369186	12183696	11200728	10369382	10653413	33995126	36948306	0
478	6583638	8304292	76300644	5524399	6719704	56751086	0	0	0
479	0	16047237	15131995	0	0	0	0	44662876	45130566
480	99441003	9644816	9775086	0	0	0	0	36161197	34452747
481	31148724	23979332	19982777	1864042	24345428	22667484	13386538	13996241	14080077
482	0	0	0	0	0	0	38876414	39517998	39661997
483	0	0	0	12477795	13942181	1378964	3602818	37662803	38120425
484	5335859	34260544	3340632	33650383	43382454	3435207	12114962	12489998	12422788
485	18274324	13698116	11196456	1333202	11867633	0	29616914	3010016	30310364
486	14311328	15659784	16028932	64033356	66562476	6124187	0	0	0
487	0	0	0	0	0	0	713919	7714267	7563252
488	0	0	0	0	0	0	8072431	88482513	8456833
489	9075536	17050952	17636707	104246025	0	30843643	0	32097525	3484935
490	532091	6240118	63954034	50875232	5162573	49972192	0	0	0
491	32637212	35662085	35741711	5543924	5769897	5623458	0	0	0
492	6507063	7209664	7227663	14811355	15954492	14253058	0	0	0
493	12965923	9671053	0	0	16687906	14887931	7749351	7763385	7644708
494	265115	21935291	1882347	2431492	0	42436703	35930557	36206655	36636208
495	895686	10141182	97628827	19863794	22276982	21273267	2991681	30214873	29023838
496	32189047	32668024	3256735	58425977	638198	5792869	50347443	51845386	5010643
497	18548897	18243816	0	49178986	3837913	3798253	72917303	69363116	70464453
498	5174776	53056555	5125048	10658293	11004255	10373959	0	0	0
499	20827239	22596096	22570774	11768177	11489944	11128118	43434624	42226504	0
500	55297983	6347597	62031416	10669918	10986622	10999679	0	49448066	48257944
501	16560382	19005298	18637676	31549294	33199475	32413284	23377712	23275815	22740093
502	3973674	59377826	5514026	18798759	19415936	18922621	36795764	34718167	30059586
503	7895515	7708257	75028457	40951267	0	39318171	22421895	22545209	0
504	55097546	47867138	0	0	0	0	18212394	18346934	18423204
505	4953898	52669104	5052439	15391313	15760156	15142063	97277985	98827795	10441077

506	0	0	0	0	10153081	88808945	14302482	13980011	14615189
507	4064588	47443076	4476752	623127	70339374	6343235	12813383	13222191	13951334
508	35084262	36182553	3413546	21682074	22511894	20165825	7247493	80294147	79174506
509	2226783	2533694	25462022	72395615	8896149	82127686	72960016	7771012	7590404
510	29937555	32228708	32698532	13023889	13454662	12541251	44871246	41902814	43705838
511	5530805	60277814	59335114	28083884	28646453	2728305	5892936	61836774	6140966
512	2105971	23857298	25492531	11515265	1168184	0	0	0	0
513	30620935	34440543	31124316	14066959	1509203	14763763	0	0	0
514	17450607	18490932	17316371	500027	56989048	58361908	75357947	77392834	7679487
515	19799728	20855305	19955929	11494825	11673907	11307505	33739468	35901575	35908591
516	13984892	14042857	15028268	38623196	42070606	4326042	7031802	7322238	7309425
517	47290536	50768454	5216267	17246509	1810592	1770413	55469745	57318536	5349282
518	9667698	10367552	10593312	15212231	15057555	14327498	14274719	14880914	13299376
519	18927197	2011091	2056853	60238153	6442386	62730664	278386	25651172	28198596
520	15318094	1670011	1568419	56104313	53389095	55463642	643903	7487287	67953644
521	19590137	23019854	21740344	31218586	32753752	30831746	12590979	1293572	12559861
522	4271677	4993894	47597446	68939663	70985225	0	19876913	20372668	19314344
523	97470184	0	9734815	12061055	13734421	12711182	91749927	9156993	86563556
524	95740375	10575312	10254978	1302694	13683778	13523916	82060565	85309674	8511137
525	0	0	0	0	0	0	77792487	7673996	7911044
526	0	0	0	0	0	0	15640869	14994404	15341891
527	2606913	2753657	2681658	7637081	7977246	7591624	57236163	6059205	58718854
528	4711664	46994608	4458142	12944873	13536729	12827417	10764462	11422826	10901138
529	63450653	65491644	59225287	16082017	17350236	16329436	13892148	14368989	14368684
530	25235347	25609072	23289849	8852522	85398145	825853	31149028	33721772	33330354
531	27480435	29363088	2961783	58161777	5834055	58260317	1693258	18677947	18250223
532	31574312	32888904	3364825	65446797	6432898	6566981	19115737	21692446	2068385
533	8474313	9113152	8898528	15462672	15595778	15260525	38463945	4151109	40890557
534	6356048	6465572	5914902	15685106	16660144	15481617	13251173	13399138	13949503
535	5006677	5059456	5139387	14330853	14606342	1378537	10913951	11147032	11562858
536	40874692	4245806	458231	11320013	0	11312997	85038153	8563916	8878759
537	21520381	23601031	22341049	52711816	5503043	50985062	22132373	21146963	0
538	32979206	32094475	33070732	26847088	25138638	25138638	70272253	64701483	6696823
539	29190106	30852795	32436163	12804841	1225173	12036648	0	0	0
540	24681017	29214514	32375146	11319098	10776055	10865748	0	0	0
541	43983463	50097278	46567496	6067137	7070852	6808788	1852327	18021108	19413801
542	3052941	30773474	32167694	96487823	9472141	92765845	0	0	0
543	2544982	29644678	32164642	10290367	9905355	10285486	0	0	0
544	11244963	12162341	12282848	15582294	16463062	15641785	11206829	11029272	1121171
545	83119196	8866556	90740106	24583391	23164766	2022379	26477026	25252432	2616432
546	2610391	28601606	28109207	0	25105078	24700542	2641845	27749517	26656414
547	68709937	74843276	7572191	67199175	65156665	63304214	6455138	0	61038076
548	12098519	11989909	12047997	10568601	10730507	11197615	0	10926033	11740109
549	13921588	14006431	13925371	13239855	13264688	12979347	12874979	13568366	13157392
550	8455643	8290717	0	0	8586522	8419796	7950796	81295425	8316008
551	0	12798678	12931571	12879555	12821864	12997042	12893375	12902039	13177894

552	8995696	8933063	8970283	9072241	9121268	9150647	8539692	8676491	8639729
553	0	11494733	11563651	12184917	12153585	0	11890728	11976639	12150565
554	4704769	46147705	4694976	8839892	8837451	0	8318448	8776191	0
555	44363896	4420068	4422295	9764347	9877776	9472141	9127156	9272222	9201534
556	38506658	38625637	39417932	9219626	9525408	9090119	0	8723534	8632102
557	29935417	30177043	30516902	75972686	7822174	74299624	6860255	7083116	6989975
558	0	10012286	9929243	11700235	11727388	11680558	11372609	11376362	11587509
559	77332427	78855693	7630522	9576265	9580353	9612417	9204494	9339736	9340407
560	79080234	7920562	7867417	86426875	8746049	8722223	81855854	8205843	8267927
561	5913712	6055422	5929576	6830967	68607734	6865502	63979966	64201763	64477246
562	0	0	0	11982648	12067522	12020265	11890728	11976639	12150565
563	5255928	5657109	59624945	16856006	0	16282454	14887931	10799851	12111698
564	20760732	28171136	2598676	7832333	7242002	6838685	0	0	0
565	9703392	11617772	11583909	9616444	87411676	8593509	3353171	34530847	34870706
566	8094397	93086176	10184504	12715757	0	97882043	50878284	48883057	50893536
567	28531134	32634467	29711795	87802185	8905301	9027333	12937551	13789946	13195344
568	767064	8056262	7920196	1509264	15761986	15078911	13974519	14041637	14266177
569	11548824	11752618	11321234	2680163	0	2592239	24110515	25267686	24860098
570	1212146	12372542	12788672	3117008	3157309	30922964	28912483	29821929	29846946
571	10717784	10785208	11094559	28796248	29683118	28861536	26167063	27279387	2813941
572	9373905	1003654	10016099	26606074	27462131	0	2299392	23508896	24215464
573	9396786	9676545	9763188	0	26697292	26929155	24189531	24931487	24721897
574	35578494	3756457	36838477	977173	9637495	8801574	9879423	10950867	10582329
575	20837002	21948716	0	0	0	0	0	0	0
576	14972438	16769668	0	39035056	28341983	27927072	0	0	0
577	14027909	14874507	15355009	14332074	15878528	15068539	11371267	11839565	12272781
578	10255558	9355143	9340621	30612393	25516023	2475149	27069889	28762049	29579572
579	73500006	7248409	7305154	13202971	12617521	1164523	10834325	1246132	11246489
580	9329973	9048994	9537123	22247693	23824045	2196824	15122538	16661058	15213147
581	11090288	12180035	11848718	3007118	32286064	30975742	20104808	19456512	20592632
582	13878114	14185635	13864081	37289385	38139644	34111362	2337344	2558924	2378957
583	7461355	75962006	7307899	22128103	23169343	20210671	12046715	13708185	12505862
584	8739337	8627373	816304	24798167	27406301	23982688	14138043	15806833	14661866
585	11451199	11949089	11271201	32463315	3387645	31431838	19873862	2191363	20170706
586	5754124	5749853	62471344	6086357	62483545	5040846	6860346	7398508	7132783
587	0	28088462	29217563	791501	51601324	4655224	0	0	0
588	10002005	0	9502039	0	0	0	0	0	0
589	8053516	6802686	69216675	7169393	55890755	4878238	6628729	69062607	72777573
590	7231629	16770277	1768552	24134923	42711277	4139943	7810062	35401547	3911743
591	3908082	63838104	51107092	21966104	1585229	16430723	3264118	18900961	15531345
592	9784971	10013964	9927016	33773333	2641967	26182012	31590908	0	30554793
593	71150885	7536405	0	73448145	7319493	7627929	8544696	82640216	806694
594	2469627	55257407	54702466	14688408	14423293	13918995	17968908	9423358	0
595	7472033	20570361	19906201	4874272	45402087	45460052	0	33042358	34676064
596	19753966	3514528	29071124	759559	7734707	79494836	37628635	29309088	29098584
597	2227088	3647238	38678113	11516791	9872407	10198843	17721824	9210077	92509576

598	1299033	11226964	12898805	34199528	3609103	36335094	64097424	6860346	7239561
599	12017733	14335735	14271667	14564545	24681017	26053879	0	101301996	1122971
600	4253738	4572242	4836442	15162503	1435709	14842169	24467766	25973032	26432483
601	17248033	61644574	6181237	6620248	10406297	9939524	0	11878616	12836265
602	39621726	13341477	13801539	15160673	32582602	293579	11681534	2886001	29632168
603	8860638	30908625	30181924	32469114	42781445	409113	0	6455413	66372104
604	4300812	15720801	15474906	15721411	19076686	17401794	0	31376008	31847358
605	18164496	21956647	21990205	58605972	50429813	46799355	11655298	98037634	1100395
606	5426163	13572118	14324752	15840087	1166506	11527468	10612532	33403268	40847235
607	43117035	61638477	6261168	57208704	57468024	0	19878439	11777634	11924073
608	18280426	3207922	33092087	23832892	21801056	19570917	16743125	94776324	9745493
609	46759695	13695066	14118213	9367498	8273784	77520966	6883624	2766684	28854214
610	2075219	0	3189312	299223	40697746	4011809	0	19233804	20014504
611	19831456	2950434	31347028	0	4502989	42863815	0	1814436	18738962
612	44064614	17855144	19518749	19462919	20239044	20406837	0	3588876	36543464
613	8765025	34532373	36979424	38983804	39983856	39190646	0	72025854	7448023
614	18854893	9522174	9596919	9927016	14064214	12856094	1458285	17263287	18096768
615	4343737	20139587	20805273	2146028	33775467	37146606	116174675	37789106	3859452
616	1659028	25614563	31304315	3383343	26755564	24497969	13158124	95831903	9034655
617	6604689	9200619	98492206	31917526	16181473	1784111	22208948	17285254	16593026
618	4483189	5282622	51094893	47353994	4499145	0	3917936	40438123	37043796
619	5272036	6880329	69108677	6700972	55491406	55999365	4830188	4519219	4150438
620	9870729	0	15206893	15957421	11523808	11792705	9484405	7290815	7166525
621	80126355	10656769	10387688	10384027	8073652	9060282	7202037	60872723	6143407
622	3561205	39602505	23189172	24571187	22399014	22278506	8115143	43473978	2859825
623	4941115	15095386	16217777	18874114	11068322	1121171	0	38672925	39967688
624	38812957	14570952	14552343	14834846	2583727	23012225	8301242	34120513	35025383
625	0	0	0	2634584	16484723	16604314	27057898	25902406	0
626	11575671	12132748	13174904	26300995	24909827	22478334	30782627	33924957	12685249
627	24208142	31560583	30395175	65049274	43232965	41225534	77084705	6910685	6897871
628	17844162	16395029	12975076	51039974	41695358	0	17047291	8608763	63749634
629	6151034	16425537	1640113	0	0	0	0	0	0
630	5359655	6730687	9161569	10720531	6791093	11501536	0	0	0
631	15427924	18326189	19558714	8328699	52443348	47409515	6730687	4761697	51985724
632	75617267	8381783	92805505	9252178	7403085	7333526	6993361	67023145	668523
633	15179893	17118679	18033921	18041244	14460818	14610002	13277106	13033651	12904297
634	66004175	7850638	80965326	9130145	48721362	5157081	74323724	6091848	5902393
635	5688532	6690111	6574486	8170972	7299357	70803094	6844482	66867554	6298083
636	72764764	8447985	84601886	12338068	99712524	97290186	9949897	8934589	8786625
637	9113366	10867274	1158879	14102959	10962155	11424352	11905464	11075034	10476161
638	9196348	10380975	10103658	14650579	10920054	99444055	12713011	10878562	1058294
639	91063495	9987117	9874542	14019061	11067407	10787039	11903022	9973388	97134595
640	75659973	8730185	9359871	11763296	83479193	9067909	982817	95276654	7855824
641	81804297	96570197	9889186	12600437	9322041	93732947	10320265	9748849	87969977
642	18206292	0	44480743	47171555	99639305	9875457	2934875	12990024	12317322
643	6158051	72053925	7248714	99456256	7186782	6517131	77209784	7087326	6363675

644	49728128	4688173	536057	62163214	62205927	64317084	5750463	62096094	57611414
645	6787737	6301744	6461606	7889078	9541699	9149366	0	8840929	7795418
646	5954257	6382285	6426827	77884015	8082804	79137897	71150885	7704809	73716614
647	47342398	53572144	5645516	6999158	7161156	6514385	5724531	59481555	6500961
648	0	0	0	88869965	23404863	22878906	0	0	0
649	2206922	26819326	28289204	4330527	40371003	41169707	65688726	68263604	64533687
650	31987695	42967545	448804	7491558	6829228	664618	86044916	799189	7666369
651	1903245	39364542	39520135	42891275	9460548	8926657	58728004	1729227	16284285
652	14495292	32024304	34327664	3681407	4112486	42772293	0	13262766	11803567
653	29799353	7060784	7564472	0	10330027	8740558	0	26716514	25126433
654	42433655	5532026	50225412	9457802	82463275	7435423	5359045	5201013	5075015
655	82579205	11347166	21032253	2590744	19296344	57782257	0	0	0
656	30062637	37249724	41451294	54622534	47298774	4405912	41533667	35323752	34656846
657	14055671	20511785	24281055	33013071	22490537	2100083	25402532	16708346	16414249
658	37665244	3885811	45926825	49227798	102241644	992366	14503529	36042215	3458698
659	20849205	23039684	23994585	11129949	11049103	100606415	77847406	76178613	72706793
660	624927	982756	11487502	11964343	8886691	7827757	8192328	5145184	5063422
661	11046661	27057593	29141293	3659136	79717545	7355492	0	12485116	11742245
662	28973499	65512994	81310065	98763727	19454987	18500084	0	35862524	34214172
663	73307806	81572437	8685033	16875836	15959069	15561244	25163044	23018328	22696162
664	15824528	18430221	2005386	0	3650899	3538019	0	0	0
665	19973623	23794758	23516829	26152725	22068306	21829429	24822878	2390184	2246003
666	0	2105971	20464803	0	0	0	776308	6336828	6557096
667	4533985	53284453	54661587	6320171	55401714	54627725	4225762	3944478	36609055
668	66162817	74538193	8079204	40325547	37894666	37465723	24759514	23831703	24233127
669	70300933	8330347	90731875	5296168	45541816	4624838	15189747	14459415	14228987
670	70006836	57367346	61455426	12987279	9039536	104856186	39373086	41773457	4288426
671	13375342	14351904	14988912	38284558	38864212	3203956	9778197	9814838	10923379
672	36898577	38463945	37881243	17453048	16623839	16121067	11651453	11694012	0
673	41709395	4511287	4443498	4806788	4453688	4607174	3420685	35802422	31890679
674	6599838	7296581	7112526	81031836	70956855	71395864	5029802	50805674	47614834
675	10422649	10990404	11257595	13006499	10745608	10701768	66283325	6610241	6412366
676	1310138	12394203	13285648	13039752	12497319	12470472	85608655	8900115	8980351
677	102348425	8770151	9384278	13014737	11483232	11647975	25498022	29939995	26644514
678	0	0	0	28097918	29018042	34193123	0	0	0
679	19856472	17582402	18892113	24873218	2109327	22777313	4986145	5943823	5187132
680	15543243	15913307	15455686	20626495	1738654	0	4696349	5015799	4910851
681	8321377	87582526	8239005	28058258	20275653	20519717	27562197	29607761	2740569
682	2196885	23123274	21800752	6032968	5311757	50978958	7107095	7701575	7246853
683	15543243	15913307	15455686	20626495	1738654	1806992	46410684	48448315	4910851
684	0	25199654	20592937	28128426	2899821	36177976	0	0	0
685	8321377	87582526	0	28058258	0	18170598	27840125	29759692	26767463
686	19515393	19073636	21654922	39718436	33244626	3612764	0	0	0
687	14480953	0	9854712	67505176	63996747	14396751	0	0	0
688	7282273	69253284	48437637	29818573	28168088	7245663	0	0	0
689	64321353	55697334	5452125	20038911	15612192	15599073	0	22957434	22702172

690	20522158	2067348	21571636	41832645	35523578	41344516	0	20104809	17938736
691	17993651	20211586	21596652	6964989	63197437	64069965	1598012	17212645	16751973
692	14363193	16941122	17237051	43168896	39568947	3923336	8089821	8084329	72581714
693	1348151	14640816	1670011	33375813	31575836	3276565	67737036	7287459	7051022
694	40874692	43379404	59557825	26538956	2780199	25599309	20125858	18631575	13078193
695	32173795	33607672	41231638	1757264	17581792	16205879	13571204	13890928	12241663
696	11230015	13887267	17923482	8816828	8200565	8725304	66882806	6276422	3927302
697	12731011	15830629	25858627	6867363	583314	75232864	10499347	10089624	6392048
698	0	0	0	0	0	0	60781195	6549469	44865146
699	26170725	23680352	7321018	56732776	7104411	1087002	0	0	0
700	5141858	56604644	17376473	14225906	1498342	22588164	0	70168524	45257173
701	1632181	14817763	16425537	219658	18396357	18121784	69820734	799006	7643183
702	117272964	12798129	21855971	0	13728624	15040471	8844285	7849722	66007227
703	15573447	1765135	19323497	25486428	25463853	2510874	34844165	39575354	3566178
704	28115308	33978347	4796751	7299052	7919891	8581611	11435884	12145928	8481209
705	29080278	33228152	4249406	67822455	7571489	7412542	14400016	15308851	12224975
706	108730705	1068087	16562823	0	0	0	18211478	18220021	1221878
707	0	0	0	41695356	39370034	4384831	0	0	0
708	0	0	0	0	11537231	16556721	0	0	0
709	33508523	30661206	12684639	8099584	85050354	13136769	0	0	0
710	5873716	53917495	21513364	14093501	14561494	22667485	0	0	0
711	7760334	8437796	12320434	10911297	11529299	11332156	0	9035784	67258364
712	15979204	15418771	33042969	28352356	29731626	34124785	1763152	18231919	10541753
713	842602	7519015	13918385	12749927	12088512	14335735	73420685	79699243	55420935
